# STING agonist-boosted mRNA immunization via intelligent design of nanovaccines for enhancing cancer immunotherapy

**DOI:** 10.1093/nsr/nwad214

**Published:** 2023-08-11

**Authors:** Lei Zhou, Wenzhe Yi, Zehong Zhang, Xiaoting Shan, Zitong Zhao, Xiangshi Sun, Jue Wang, Hao Wang, Hualiang Jiang, Mingyue Zheng, Dangge Wang, Yaping Li

**Affiliations:** State Key Laboratory of Drug Research & Center of Pharmaceutics, Shanghai Institute of Materia Medica, Chinese Academy of Sciences, Shanghai 201203, China; China State Institute of Pharmaceutical Industry, Shanghai 201203, China; State Key Laboratory of Drug Research & Center of Pharmaceutics, Shanghai Institute of Materia Medica, Chinese Academy of Sciences, Shanghai 201203, China; Univerisity of Chinese Academy of Sciences, Beijing 100049, China; Drug Discovery and Design Center, State Key Laboratory of Drug Research, Shanghai Institute of Materia Medica, Chinese Academy of Sciences, Shanghai 201203, China; Univerisity of Chinese Academy of Sciences, Beijing 100049, China; State Key Laboratory of Drug Research & Center of Pharmaceutics, Shanghai Institute of Materia Medica, Chinese Academy of Sciences, Shanghai 201203, China; Univerisity of Chinese Academy of Sciences, Beijing 100049, China; State Key Laboratory of Drug Research & Center of Pharmaceutics, Shanghai Institute of Materia Medica, Chinese Academy of Sciences, Shanghai 201203, China; State Key Laboratory of Drug Research & Center of Pharmaceutics, Shanghai Institute of Materia Medica, Chinese Academy of Sciences, Shanghai 201203, China; State Key Laboratory of Drug Research & Center of Pharmaceutics, Shanghai Institute of Materia Medica, Chinese Academy of Sciences, Shanghai 201203, China; Univerisity of Chinese Academy of Sciences, Beijing 100049, China; China State Institute of Pharmaceutical Industry, Shanghai 201203, China; Drug Discovery and Design Center, State Key Laboratory of Drug Research, Shanghai Institute of Materia Medica, Chinese Academy of Sciences, Shanghai 201203, China; Univerisity of Chinese Academy of Sciences, Beijing 100049, China; Drug Discovery and Design Center, State Key Laboratory of Drug Research, Shanghai Institute of Materia Medica, Chinese Academy of Sciences, Shanghai 201203, China; Univerisity of Chinese Academy of Sciences, Beijing 100049, China; Precision Research Center for Refractory Diseases, Shanghai General Hospital, Shanghai Jiao Tong University School of Medicine, Shanghai 201620, China; State Key Laboratory of Drug Research & Center of Pharmaceutics, Shanghai Institute of Materia Medica, Chinese Academy of Sciences, Shanghai 201203, China; Univerisity of Chinese Academy of Sciences, Beijing 100049, China; Shandong Laboratory of Yantai Drug Discovery, Bohai Rim Advanced Research Institute for Drug Discovery, Yantai 264000, China

**Keywords:** vaccine, mRNA, STING, cancer immunotherapy, nanoparticles

## Abstract

Messenger RNA (mRNA) vaccine is revolutionizing the methodology of immunization in cancer. However, mRNA immunization is drastically limited by multistage biological barriers including poor lymphatic transport, rapid clearance, catalytic hydrolysis, insufficient cellular entry and endosome entrapment. Herein, we design a mRNA nanovaccine based on intelligent design to overcome these obstacles. Highly efficient nanovaccines are carried out with machine learning techniques from datasets of various nanocarriers, ensuring successful delivery of mRNA antigen and cyclic guanosine monophosphate-adenosine monophosphate (cGAMP) to targets. It activates stimulator of interferon genes (STING), promotes mRNA-encoded antigen presentation and boosts antitumour immunity *in vivo*, thus inhibiting tumour growth and ensuring long-term survival of tumour-bearing mice. This work provides a feasible and safe strategy to facilitate STING agonist-synergized mRNA immunization, with great translational potential for enhancing cancer immunotherapy.

## Teaser

This paper provides a rational design option to develop mRNA nanovaccine with machine learning techniques, leading to effective stimulator of interferon genes (STING) agonist-synergized mRNA immunization.

## INTRODUCTION

Messenger RNA (mRNA) vaccines are revolutionizing the therapy of a variety of diseases [1–3]. mRNA vaccines can be flexibly developed in a short period of time, allowing transient expression of multiple antigens for safe and efficient immunization [4]. A diversity of mRNA vaccines is being explored in clinic and two have been authorized by the US Food and Drug Administration (FDA) to combat COVID-19 [5]. However, clinical translation of mRNA vaccines is still hampered by multistage delivery barriers before initiating strong immunity [1,3,6]. First, mRNA suffers rapid clearance into the circulation and shows poor lymphatic transport through intradermal administration [7]. Second, catalytic hydrolysis in interstitial space causes insufficient cellular targeting of mRNA [6]. Third, the phospholipid bilayer of cell membranes and endosomes limits entry of mRNA into the cytoplasm [8]. Finally, vaccination with mRNA alone can barely induce strong immune responses in the absence of adjuvants [9]. It remains challenging to improve cytosolic delivery of mRNA and promote its *in vivo* vaccination efficacy.

**Figure 1.**
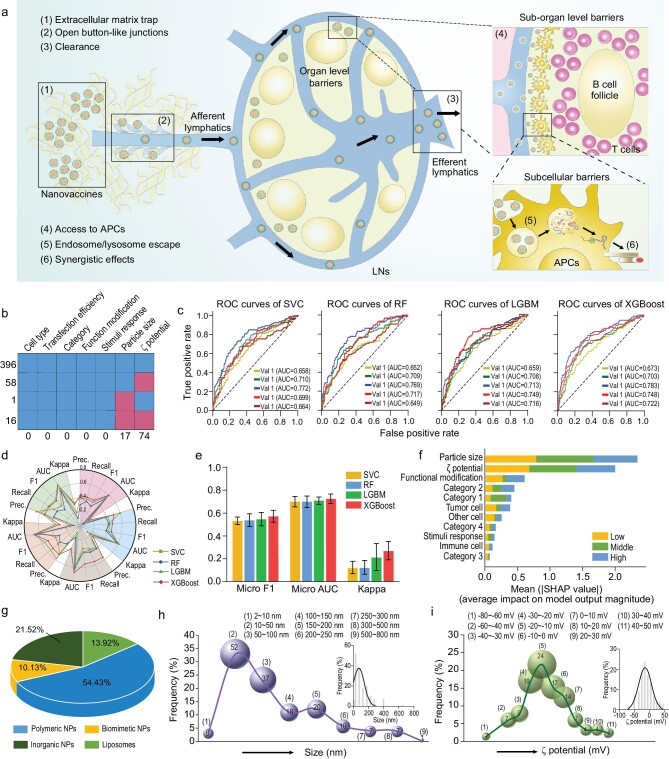
Identifying key parameters of nanovaccines from the databases of existing nanocarriers. (a) A series of biological barriers at organ, sub-organ and subcellular level hamper the performance of nanovaccines. A synergistic combination with adjuvants may improve the performance of a nanovaccine. (b–f) The nanoparticles (*n* = 471) for RNA delivery were sorted out from 262 references. The cell types, size, ζ potential and category of nanoparticles were used for machine learning modeling. (b) Analysis of missing values in RNA delivery data (*n* = 471). We use mice (multivariate imputation by chained equations) in R to fill these missing values. (c) The 5-fold cross-validation ROC curves for four machine learning models. In 5-fold cross-validation, the original sample was randomly partitioned into 5 equal-sized subsets (val set 1 to 5). Each time, a single subset was retained as the validation set to evaluate models, and the remaining 4 subsets were used for training models. The micro average strategy was used to plot the ROC curves. Macro (d) and micro (e) metrics for model evaluation. In (d), the radar map was shown to display the macro metric results in each cross-validation. Each sector with a different color was a separate cross-validation result. In (e), the bar plot indicated the mean values and standard deviation of micro metric results in 5-fold cross-validation. In particular, micro-averaged precision, recall and accuracy areall equal to the F1 score, so only micro F1 results were shown in this plot. (f) The interpretability of XGBoost model was analyzed by SHAP (SHapley Additive exPlanations) value. The different colors represented the feature importance of different classes (low, middle and high). (g–i) The database of nanoparticles (*n* = 158) for LN delivery was summarized from 86 references. (g) The pie chart of nanoparticles classification commonly used for LN delivery (*n* = 158). The size (h) and ζ potential distribution (i) of these nanoparticles were shown as bubble charts. Gaussian fittings were also performed here. (h) The particle size from 2 nm to 800 nm was divided into 9 intervals (*n* = 158). The bubble size was connected with the counts of nanoparticles in corresponding interval, and the *n* number was labeled in bubbles. (i) The distribution of ζ potential was divided into 11 intervals (*n* = 110), and the *n* number of nanoparticles in each interval was labeled accordingly.

Nanoparticles are promising to protect mRNA from degradation and deliver mRNA to lymph nodes through lymphatic vessels (Fig. 1a) [10,11]. Particles with 10–100 nm diameters show preferential access to lymphatics through the open button-like junctions between lymphatic endothelial cells [7,12]. However, strong nanoparticle-biological interactions including interstitial matrix trap and membrane deformation happen after administration, resulting in failed access to lymphatics and potential *in vivo* cytotoxicity [13–15]. Besides, most of the nanoparticles are entrapped in endosomes after cellular endocytosis, leading to degradation of mRNA and insufficient expression of encoded antigens [13,16]. Therefore, nanoparticles for mRNA delivery face the difficulty that a single platform should meet the criteria of stable loading, slow clearance, efficient lymphatic transport, high cellular entry and rapid endosome escape for overcoming the organ level, sub-organ level and subcellular level barriers *in vivo*. In the past decades, a huge number of nanocarriers have been reported to promote transfection efficacy of nucleic acid drugs (Supplementary Table 1) or deliver drugs to lymph nodes (Supplementary Table 2) [12,13]. These studies provide valuable features including size, surface charge, modification, responsiveness, components and cytotoxicity to achieve the goal of lymph node drainage or cytosolic access. Machine learning techniques provide powerful tools for exploring the physicochemical characteristics and biological features of nanoparticles, and facilitate the design of nanocarriers with high efficiency. Commonly, machine learning models were trained, selected and optimized with high quality and massive datasets from computations and high-throughput experimental data, and in turn guide the rational design, screening and optimization of nanocarriers [17–22]. By leveraging existing nanocarriers’ databases, machine learning may provide insights into intelligent design of nanovaccines with high delivery efficiency.

Adjuvants are also key components affecting the effectiveness of mRNA vaccines. Previous studies have suggested that type I interferon (IFN-I)-associated adjuvant effects will promote advantageous adaptive immunity [23]. Stimulator of interferon genes (STING) is critical for initiating IFN-I and has emerged as a promising target to initiate host innate and adaptive immunity [24]. Certain STING agonists, including cyclic guanosine monophosphate-adenosine monophosphate (cGAMP) and structure-mimicking cyclic dinucleotides (CDNs) are being tested to treat cancers in clinic [25]. However, most of them exhibit poor metabolic stability, off-target cytotoxicity and insufficient cytosolic delivery, resulting in poor access to STING and weak immune responses [26]. Recently, activation of STING has been used to improve mRNA vaccination, providing an attractive combinational strategy to boost host immunity [6]. Given that mRNA and CDNs perform corporate demands for effective cytosolic delivery, new approaches that overcome the multiple delivery barriers are highly desirable for STING activation-synergized mRNA vaccination.

Herein, tailored design of nanovaccines based on machine learning techniques is proposed to deliver mRNA antigen and cGAMP for STING activation-promoted mRNA immunization. A machine learning model based on a database of existing nanocarriers is optimized to screen nanovaccines with high efficiency for cytosolic delivery of mRNA and cGAMP. To prepare nanovaccines for screening, phenylboronic acid (PBA)-grafted oligoethylenimine (OEI) with various grafting ratios are synthesized to condense mRNA and cGAMP into polymeric nanocomplexes. Furthermore, to avoid interstitial matrix trap and severe cytotoxicity [15], the nanocomplexes are encapsulated by anionic lipids to obtain the nanovaccine. As a result, this approach facilitates lymphatic transport and cytosolic delivery of mRNA and cGAMP, thus efficiently activating STING and enhancing mRNA vaccination in targets. Using tumour antigens-encoded mRNA, the nanovaccines boost strong adaptive immunity and induce a T cell-inflamed tumour microenvironment (TME). Moreover, therapeutic efficacy is augmented when cooperating with anti-programmed cell death protein ligand 1 (PD-L1) therapy. This study presents an intelligent design strategy to develop a highly efficient mRNA nanovaccine, which is promising for STING activation-synergized mRNA vaccines against cancer.

## RESULTS AND DISCUSSIONS

### Designing nanovaccine with machine learning techniques

Machine learning techniques are important to accelerate the screening and optimization of nanomedicine. For example, the structural parameters of spherical nucleic acid nanoparticles from a library were organized via the features of core, antigen and oligonucleotide categories. By combining high-throughput experimental evaluations and machine learning–based data assay, the nanostructure-immune activation correlations were established with extremely high efficiency [17]. In order to prepare nanovaccines with the most effective efficacy, we collected the physicochemical properties of existing nanocarriers from two aspects: (1) nanocarriers for cytosolic delivery of RNA (Supplementary Table 1), (2) nanocarriers for delivering drugs to lymph nodes (Supplementary Table 2). The procedure and inclusion criteria for searching literature were provided (Fig. S1 in the online supplementary file). First, we determined the key parameters of nanocarriers that associate with effective cytosolic delivery of RNA. A dataset of nanocarriers reported from 2010 to 2021 (search topic, ‘RNA delivery or RNA vaccine’, Web of Science) was gathered. We deleted the data without transfection efficiency values and analyzed the missing values in the remaining data (Fig. 1b). It was found that there were 17 missing values for particle size and 74 missing values for ζ potential. In order to reduce information loss, we used multivariate imputation by chained equations algorithm (MICE) to impute those missing values [27]. According to the data, we divided the transfection efficiency into three levels: high (>60%), moderate (40%–60%) and low (<40%). Four different machine learning models were used in this study for classification tasks, which were support vector classification (SVC), random forests (RF), light gradient boosting machine (LightGBM) and extreme gradient boosting machine (XGBoost). We evaluated the performance of those models through 5-fold cross-validation (Fig. 1c–e). For the validation sets, the four models predicted with micro-averaged AUC (area under the receiver operating characteristic curve) all greater than 0.65 (Fig. 1c). The macro-averaged metrics for the 5-fold cross-validation are presented (Fig. 1d). The XGBoost model was always found to produce the highest metrics on each validation set. Nevertheless, macro-averaged metrics cannot be regarded as the sole approach for evaluation models, especially in the case of class imbalance. For this reason, we also used micro-averaged metrics to evaluate the model, and found that the mean F1 score (0.571) and AUC (0.726) of XGBoost were slightly better than other models, while the kappa value (0.271) was significantly better than others (Fig. 1e). To identify the principal features driving model prediction, Shapley additive explanations (SHAP) values were calculated for feature importance analysis [28]. The ranking of the most important features of the XGBoost model was summarized (Fig. 1f). We found that particle size and ζ potential were the hallmark features, which were far more important than others. The machine learning model predicts the transfection efficiency division according to the size, ζ potential, modification, category, stimuli response and cell types, helping to screen nanoparticles with high RNA transfection efficiency.

Despite lacking adequate *in vitro* cytotoxicity data of these nanocarriers for machine learning, cytotoxicity could be a major obstacle for the application of positively charged nanocarriers. It remains challenging to achieve the balance among transfection efficacy, surface charge and cytotoxicity for nanocarrier-mediated RNA delivery. Next, we determined the key parameters for effective delivery of drugs to lymph nodes by statistics. Machine learning technique was not used in this process due to the lack of a dataset for quantified lymph nodes targeting. The data were summarized with nanocarriers reported from 2010–2021 (search topic, ‘lymph node delivery and nanoparticles’, Web of Science). Polymeric nanoparticles were the most commonly used category (>50%) for lymphatic delivery, and 21.52% of nanocarriers were inorganic nanoparticles (Fig. 1g). It was interesting to find that the sizes of nanoparticles ranged from 2 to 800 nm, which was beyond the most suitable diameter (10–100 nm) for passing through the interstices of endothelial cells. According to the statistics, 80.38% of nanocarriers were distributed within 10–200 nm, indicating that a diameter within 10–200 nm might facilitate the access of drugs to lymph nodes (Fig. 1h). Besides, 70.91% of the nanocarriers were negatively charged, and 54.55% of nanocarriers were within −30 mV (Fig. 1i), suggesting an appropriate range of surface charges for effective lymphatic transport. Based on the above data, we identified the key parameters for designing a nanovaccine with high efficiency to deliver mRNA and cGAMP.

### Designing nanocomplexes for highly efficient cytosolic delivery of mRNA and cGAMP

**Figure 2.**
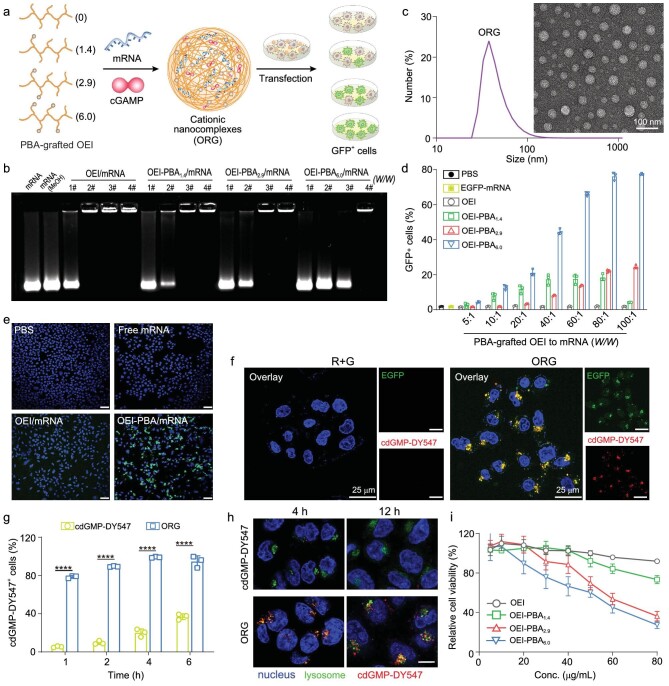
Design of ORG nanocomplexes and its transfection efficiency and cGAMP delivery capability in DC2.4 cells. (a) Schematic illustration of ORG nanocomplexes self-assembly through complexing various cationic polymers (OEI-PBA) with mRNA and 2’3’-cGAMP. The formulated ORG nanocomplexes were screened via EGFP-mRNA transfection efficiency in DC2.4 cells. (b) Agarose gel electrophoresis assay of mRNA condensation ability with OEI or OEI-PBA at various weight ratios (OEI-PBA/mRNA, 1# 0.125, 2# 0.25, 3# 0.5, 4# 1). The stability and integrity of mRNA in solvent (MeOH) was also determined as the control group. (c) Size distribution and TEM morphology of ORG nanocomplexes. Scale bar, 100 nm. (d) Quantification of GFP^+^ DC2.4 cells after treatment with PBS, naked EGFP-mRNA, and OEI-PBA/EGFP-mRNA at varying weight ratios (5 : 1, 10 : 1, 20 : 1, 40 : 1, 60 : 1, 80 : 1, 100 : 1) (*n* = 3). (e) Confocal images of DC2.4 cells with treatment of PBS, naked EGFP-mRNA, OEI/EGFP-mRNA and OEI-PBA_6.0_/EGFP-mRNA for 24 h. DAPI (blue), GFP (green). Scale bars, 50 μm. (f) Confocal examination of EGFP-mRNA and cdGMP-DY547 (replacing cGAMP) delivered into DC2.4 cells. DAPI (blue), GFP (green), DY547 (red). Scale bars, 25 μm. (g) Flow cytometry analysis of cellular uptake of cdGMP-DY547 in DC2.4 cells after incubation with free R + G (EGFP-mRNA + cdGMP-DY547) and ORG nanoparticles (replacing cGAMP with cdGMP-DY547) (*n* = 3) at different time points (1, 2, 4 and 6 h). (h) Confocal fluorescence images of cdGMP-DY547 uptake in DC2.4 cells after treatment with the same formulation in panel g for 4 h and 12 h. The nucleus and lysosomes were stained with DAPI (blue) and lysotracker (green), respectively. Scale bar, 10 μm. (i) DC2.4 cells were incubated with OEI-PBA (*n* = 6) at various concentrations (from 5 μg/mL to 80 μg/mL) for 24 h. Cell viability was examined by CCK-8 assay and normalized with the untreated group.

The nanovaccines were endowed with two desired features, high targeting capability to lymph nodes and efficient mRNA transfection in DCs. Accordingly, we first tried to screen nanocomplexes with efficient mRNA transfection via machine learning technique, and then optimize the nanocomplexes for targeting lymph nodes. We used OEI as modular chains on the basis of their superiority in gene transfection. PBA-grafted OEIs (OEI-PBA) with PBA grafting ratio at 1.4, 2.9 and 6.0 were synthesized and employed to condense mRNA and cGAMP. The efficacy of nanocomplexes for cytosolic delivery of drugs was screened by a green fluorescence protein (GFP) reporter with mRNA encoding enhanced GFP (EGFP-mRNA) (Fig. 2a and Fig. S2). First, we evaluated the capability of OEI and its derivatives for mRNA condensation (Fig. 2b). It was found that condensation capacity was decreased following increased grafting ratio of OEI-PBA. But no shift band was found in all groups with an OEI or OEI-PBA to mRNA weight ratio ≥ 1 : 1, indicating that all of the cationic polymers were efficient in condensing mRNA. Accordingly, the cationic OEI-PBA/mRNA/cGAMP nanocomplexes (referred as ORG) were self-assembled by mixing mRNA, cGAMP and OEI-PBA at indicated weight ratios. As characterized by dynamic light scattering (DLS) and transmission electron microscopy (TEM), ORG nanocomplexes exhibited a uniform spherical morphology with an average diameter of 20–40 nm at various OEI-PBA_6.0_ to mRNA ratios (Fig. 2c and Fig. S3). The surface charge of ORG at 40 : 1 was 18.6 ± 3.6 mV. In addition, we prepared a series of OEI-PBA–based nanocomplexes at different weight ratios, and the particle size and ζ potential were determined by DLS. Next, the features of these nanoparticles were screened by the XGBoost model developed in Fig. 1. According to the predictive results, certain samples with appropriate diameters and surface charges were determined to be promising (Supplementary Table 3). To verify screening accuracy, we next evaluated the transfection efficiency of mRNA nanocomplexes in DC2.4 cells. Free OEI-based nanocomplexes failed to achieve obvious GFP expression even at a ratio of 100 : 1, while OEI-PBA significantly improved transfection efficacy with increased weight ratios. When treated by OEI-PBA_6.0_ nanocomplexes, over 40% of cells expressed GFP at ratios ≥40 : 1 (Fig. 2d, Fig. S4). The expression of GFP in cells was also detected by confocal laser scanning microscopy (CLSM). DC2.4 cells were treated with OEI-PBA_6.0_ nanocomplexes at 0.5 μg/mL of mRNA for 24 h. Compared to cells treated by PBS, free mRNA and OEI/mRNA complexes, bright green fluorescence in cytoplasm was observed after treatment by OEI-PBA_6.0_/mRNA nanocomplexes (Fig. 2e). In order to further verify the accuracy of the XGBoost model, PBA-grafted polyamidoamine (PAMAM, G2) nanocomplexes were prepared, tested and predicted (Fig. S5). According to the results, 1/28 of the nanocomplexes fell into the range of moderate transfection efficiency (40%–60%; Supplementary Table 4). The other nanocomplexes were predicted to show low RNA transfection capability (<40%). Similarly, experimental transfection efficiencies of PAMAM-PBA/mRNA were determined. It was found PMAMA-PBA_6.6_/mRNA (100 : 1, w/w) showed the highest transfection efficiency of ∼20% (Fig. S6). It suggests that the machine learning model is helpful in accelerating the screening of nanocarriers with high RNA transfection efficiency. In following studies, we used OEI-PBA with high transfection efficiency to prepare the nanovaccines.

Next, we detected the efficacy of ORG nanocomplexes for cytosolic delivery of cGAMP. cdGMP-DY547 (replacing cGAMP) was used to prepare the ORG and to treat DC2.4 cells. After incubation for 24 h, the ORG enabled obvious expression of GFP and cytosolic dispersion of cdGMP as examined by CLSM, while free mRNA and cdGMP treatment resulted in weak transfection as well as poor intracellular entry of the drugs (Fig. 2f). The ORG led to highly efficient intracellular delivery of cGAMP within 1 h since ∼80% of cells were detected as cdGMP positive at that time (Fig. 2g). Moreover, the nanocomplexes significantly improved intracellular signals of cdGMP, which was 68.8-fold higher than free cdGMP at 4 h (Fig. S7). The intracellular localization of ORG was imaged with cdGMP-DY547 to replace cGAMP. In ORG-treated cells, the signals from cdGMP were well co-localized with lysosomes at 4 h as orange dots were presented in cytoplasm. The overlap between cdGMP and lysosomes was reduced at 12 h (Fig. 2h, Fig. S8). Pearson correlation coefficient (PCC) between cdGMP-DY547 and lysotracker green signals was 0.78, showing strong correlation of these signals (Fig. S9). Regarding GFP expression and localization of cdGMP-DY547 in cells, the nanocomplexes derived from OEI-PBA_6.0_ excellently performed cytosolic delivery of mRNA and cGAMP. Unfortunately, the cell viability results showed that the toxicity of OEI-PBA in DC2.4 cells was elevated following increased grafting ratio of PBA (Fig. 2i). OEI-PBA_6.0_ resulted in the highest cytotoxicity at the same concentrations compared to free OEI. Although OEI-PBA_6.0_ was optimized as the most effective carrier for mRNA transfection and cGAMP delivery, it remains challenging to avoid its cytotoxicity towards DCs for effective antigen presentation and STING activation.

### Encapsulation of ORG with lipids for highly efficient lymphatic transport

**Figure 3.**
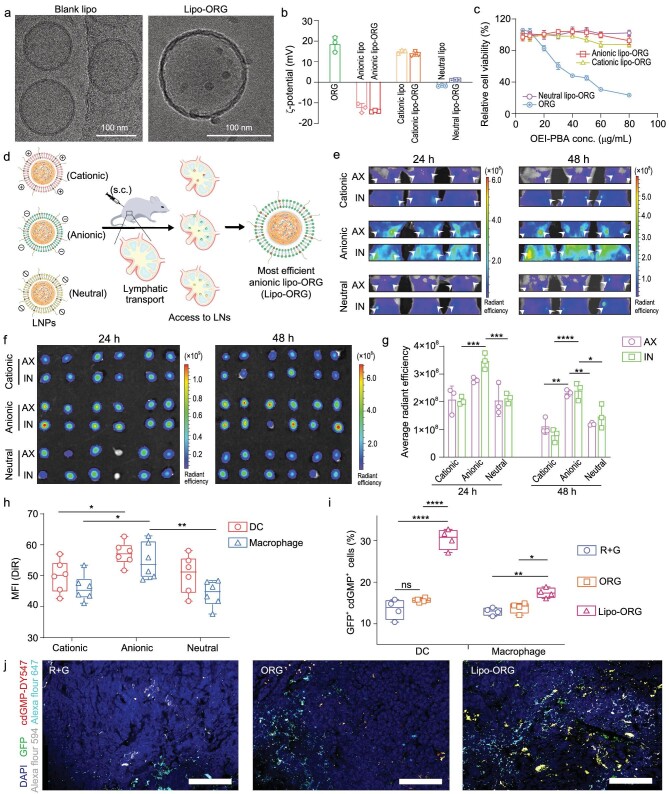
Preparation of the nanovaccine for efficient LNs delivery. (a) Representative cryo-TEM images of liposomes and Lipo-ORG. Scale bars, 100 nm. (b) Zeta potential of ORG nanocomplexes, liposomes and Lipo-ORG (*n* = 3), prepared by different lipids to regulate the surface charges (referred as cationic Lipo-ORG, anionic Lipo-ORG and neutral Lipo-ORG, respectively), were measured in 50 mM HEPES. (c) Cell viability of DC2.4 cells after treatment with ORG nanoparticles and various LNPs (anionic Lipo-ORG, cationic Lipo-ORG, neutral Lipo-ORG) for 24 h (*n* = 6), as measured by CCK-8 assay and normalized with the untreated group. (d) Scheme of three kinds of LNPs accumulation in LNs via s.c. administration and the most efficient LNP was optimized as anionic Lipo-ORG (referred to as Lipo-ORG in the following experiment). (e and f) Representative living images (e) and isolated LNs (f) of DiR fluorescence signals at 24 h and 48 h post administration. DiR was encapsulated into nanoparticles and s.c. injected into mice (*n* = 6) at tailbase. Mice were anesthetized at indicated time points for live imaging (e) and LNs were harvested for *ex vivo* imaging (f). (g) Quantification of fluorescence intensity in excised LNs (*n* = 6) in panel f. (h) The cellular uptake of LNPs (DiR) in DCs (CD45^+^CD11c^+^MHCII^+^) and macrophages (CD45^+^CD11b^+^F4/80^+^) from LNs (*n* = 6) were assessed by flow cytometry at 24 h. (i) Percentage of GFP^+^cdGMP-DY547^+^ cells among DCs and macrophages in LNs (*n* = 4) were measured at 24 h after mice vaccinated with mRNA + cdGMP-DY547, ORG nanoparticles and Lipo-ORG. (j) Fluorescence signals of transfected GFP protein (green) and cdGMP-DY547 (red) in LN sections from mice treated with various treatments. The cell nucleus was stained with DAPI (blue). The DCs were stained with CD11c-Alexa Fluor 594 (silver), and the macrophages were stained with F4/80-Alexa Fluor 647 (wathet). Scale bars, 50 μm.

Lymph nodes are referred to as the critical secondary lymphoid organs where plenary antigen presenting cells (APCs) reside. Antigens are transported to lymph nodes and processed by APCs to elicit robust antitumour immunity [29,30]. A diversity of nanoparticles has been applied to promote lymphatic transport of antigens to lymph nodes, and transport efficacy is closely associated with the physicochemical properties of nanoparticles including size, surface charge and chemical modification [12,31,32]. Recently, a study on selective organ targeting lipid nanoparticles has revealed that internal charge is important for tuning organ tropism of delivered mRNA and gene editing drugs through systemic administration [33]. The extremely high ζ potential of ORG leads to strong nanoparticle-biological interactions, which go against efficient transportation of drugs to lymph nodes. Therefore, we considered that if the surface characters of ORG could be changed, lymphatic transport of drugs may be promoted. We encapsulated the ORG nanocomplexes with different lipids to change the surface characteristics, and explored the transport efficacy of these nanovaccines to lymph nodes. The lipid, 1,2-dioleoyl-snglycero-3-phosphoethanolamine (DOPE), was selected as the base component to fabricate the nanoparticles [6]. Then, the lipids of 1,2-dioleoyl-3-trimethylammonium-propane (DOTAP) and 1,2-distearoyl-sn-glycero-3-phospho-(1’-rac-glycerol) (DSPG) were used to provide positive or negative internal charges, respectively. Other lipids including 1,2-distearoyl-sn-glycero-3-phosphocholine (DSPC), 1,2-dihexadecanoyl-rac-glycero-3-phosphocholine (DPPC) and 1,2-distearoyl-sn-glycero-3-phosphoethanolamine-N-methoxy (polyethylene glycol) (DSPE-PEG) were used to stabilize the nanovaccines. The obtained nanovaccines with various surface charges (referred to as anionic Lipo-ORG, cationic Lipo-ORG, neutral Lipo-ORG) were first determined by DLS and cryo-TEM, showing homogeneous size at around 100 nm and consistent surface charges (Fig. 3a and b). The loading capacity (LC) and encapsulation efficiency (EE) of cGAMP within ORG was 2.16 ± 0.02 wt% and 90.6 ± 0.72%. The LC and EE of cGAMP within Lipo-ORG was 0.21 ± 0.003 wt% and 94.85 ± 1.11%, respectively. Besides, it did not show any significant difference between the release profile of ORG and Lipo-ORG (Fig. S10). Less than 8% of total cGAMP amounts was released from the nanocarriers within 48 h, indicating that cGAMP were stably loaded in the nanoparticles. As expected, the encapsulation of ORG with lipids, especially anionic Lipo-ORG, reducing the cytotoxicity in DC2.4 cells when compared with ORG *in vitro*, which is helpful to maintain the viability of APCs for antigen presentation (Fig. 3c, Fig. S11).

To evaluate the lymphatic transport *in vivo*, DiR-loaded Lipo-ORG were administered subcutaneously, and the mice were examined by an *in vivo* imaging system (IVIS) at 24 h and 48 h, respectively (Fig. 3d). Given the key parameters identified for effective lymph node delivery in Fig. 1, all Lipo-ORG were predicted to be effective for lymphatic drain (Fig. S12). According to the *in vivo* results, higher accumulation of anionic Lipo-ORG in axillary and inguinal lymph nodes was achieved than that with cationic or neutral ones (Fig. 3e). Additionally, the axillary and inguinal lymph nodes were harvested for imaging *ex vivo*. Similar trends in lymph nodes were observed, with anionic Lipo-ORG being most effectively accumulated (Fig. 3f and g). The results were probably due to the fact that the anionic surface minimized electrostatic interactions with the negatively charged glycosaminoglycans in interstitial matrix, leading to faster draining and high accumulation of nanovaccines in lymph nodes [34,35]. Afterwards, we further assessed APCs’ uptake on Lipo-ORG *ex vivo* by flow cytometry. Consistent with the *in vivo* distributions, anionic Lipo-ORG conducted the highest uptake efficacy in both DCs and macrophages in contrast to the cationic or neutral ones (Fig. 3h, Fig. S13). Together, these findings suggested that anionic LNPs could serve as an optimal delivery vehicle for highly efficient *in vivo* lymphatic transport.

In order to determine whether the nanovaccines would affect the cytosolic transfection of mRNA and delivery of cGAMP, we next examined the transfection efficiency *in vivo* by co-delivering EGFP-mRNA and cdGMP-DY547 via anionic Lipo-ORG. In contrast, anionic Lipo-ORG showed superior *in vivo* performance than ORG nanocomplexes. Anionic Lipo-ORG induced a 2.0-fold higher percentage of GFP^+^cdGMP^+^ DCs and 1.3-fold of GFP^+^cdGMP^+^ macrophages than ORG nanocomplexes, whereas no obvious differences were detected between free drugs and ORG (Fig. 3i). Moreover, we investigated the transfection of mRNA and delivery of cdGMP in lymph nodes *ex vivo*. The co-localization of GFP and cdGMP-DY547 with DCs was confirmed in lymph nodes after treatment with Lipo-ORG for 24 h, as obvious yellow signals were observed. The fluorescent signals in the Lipo-ORG group were higher than that in both ORG and R + G groups (Fig. 3j, Fig. S14). But the co-localization of GFP and cdGMP-DY547 with macrophages was much weaker than that with DCs. Although ORG also led to GFP expression and delivery of cdGMP-DY547 in lymph nodes, the efficacy was much weaker than anionic Lipo-ORG. Hence, with a two-step optimal screening, we have developed a nanovaccine for efficient lymphatic transport and cytosolic delivery of mRNA and cGAMP.

### Potent antitumour immune responses induced by STING agonist and mRNA antigen

**Figure 4.**
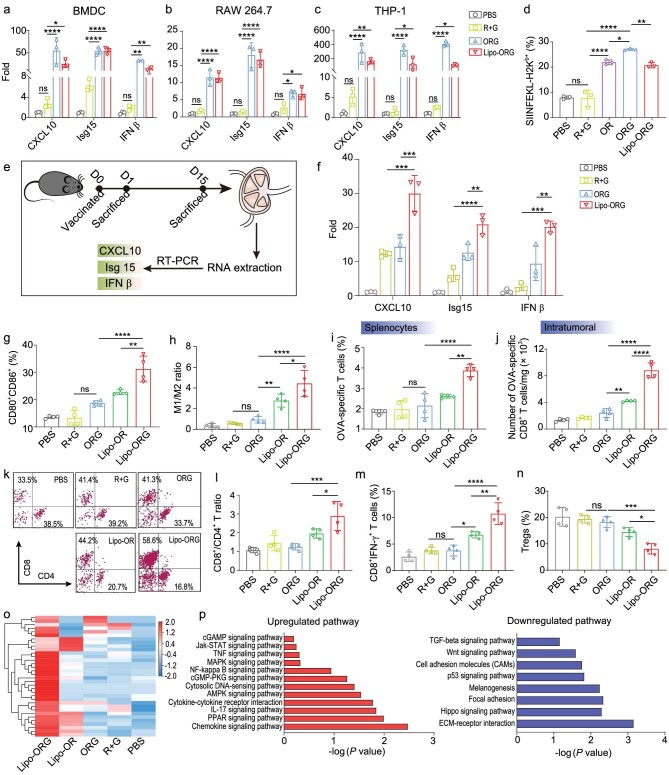
Promoted STING activation and robust tumour-specific T cell responses stimulated by LNP vaccines. (a–c) mRNA expression profile of STING-activated genes (e.g. CXCL10, Isg 15 and IFN-β) in BMDCs (a), RAW 264.7 (b) and THP-1 cells (c) after treatment with PBS, OVA-mRNA + cGAMP, ORG nanoparticles and Lipo-ORG (*n* = 4) for 24 h. (d) Quantification of antigen-presentation efficiency (CD11c^+^ SIINFEKL-H2K^b+^) following with the same treatments in panels a–c (*n* = 3). (e) Schematic illustration of the timeline for analyzing STING-activating genes expression in LNs. Mice (*n* = 3) were immunized on day 0, and LNs were harvested on day 1 and day 15, followed by RNA extraction for Real-Time PCR determination of indicated genes. (f) Quantitative assay of stimulated genes (e.g. CXCL10, Isg 15 and IFN-β) expression in LNs on day 1 after single-dose vaccination. (g) The percentage of CD80^+^ CD86^+^ DCs from LNs on day 7 after mice (*n* = 4) were immunizedwith indicated formulations. (h) M1 to M2 macrophage ratios in tumours of mice with various treatments. (i and j) Production of OVA-specific CD8^+^ T cells in splenocytes (i) and within TME normalized by tumour weight (j) on day 7 after vaccination, as measured by flow cytometry analysis of CD8^+^ OVA-tetramer^+^ T cells (*n* = 4). (k–n) Analysis of tumour-infiltrating lymphocytes on day 7 after single-dose immunization with indicated formulations. (k) Representative plots of intratumoural CD8^+^ and CD4^+^ T cells. (l) The ratio of CD8^+^ T cells to CD4^+^ T cells in TME according to the dot plots in k (*n* = 4). (m) The frequency of CD8^+^ IFN γ^+^ T cells among intratumoural lymphocytes was assessed by flow cytometry (*n* = 4). (n) The frequency of Tregs (gated on CD4^+^) in TME (*n* = 4). (o) Clustering heat map of selected gene expression. (p) Representative upregulated and downregulated pathways in TME as determined by KEGG pathway analysis.

Given the importance of the STING pathway in antitumour-specific T cell immunity, we investigated the capacity for STING activation after treatment by various cGAMP formulations for 24 h in bone marrow–derived DCs (BMDCs), RAW 264.7 and THP-1 cells. The mRNA transcription level of STING-related genes was detected by real time polymerase chain reaction (RT-PCR) (Fig. 4a–c). Compared to free mRNA + cGAMP, ORG nanocomplexes dramatically increased the expression of CXCL-10, Isg15 and IFN-β in BMDCs, showing 20-, 8.5- and 17.3-fold high levels, respectively. Consequently, a similar tendency of these genes’ expression was observed in RAW 264.7 and THP-1 cells, implying the activation of APCs. Comparable elevation of CXCL-10, Isg15 and IFN-β were achieved with anionic Lipo-ORG treatment. We evaluated the marker proteins of STING pathways including TANK binding kinase 1 (TBK1), phosphorylated TBK1 (p-TBK1), interferon regulatory factor 3 (IRF3) and phosphorylated IRF3 (p-IRF3) via Western blotting in BMDCs (Fig. S15). The expression of IRF3 in ORG and Lipo-ORG groups were downregulated when compared with PBS or R + G group. While the expression of p-TBK1 and p-IRF3 were upregulated after treatment by ORG and Lipo-ORG *in vitro*. As reported, the activation of STING will lead to a downstream TBK1-IRF3 cascade. After STING activation, TBK1 is recruited, phosphorylated and in turn promote the phosphorylation of STING. Then, the complex of STING-TBK1 triggers the phosphorylation of IRF3 and downstream IFN-I response [36,37]. In combination with the enhanced expression of CXCL10, Isg15 and IFN-β mRNA, the obvious enhancement of p-IRF3 suggested that the ORG and Lipo-ORG group enabled activation of STING pathways *in vitro*. The IFN-I in BMDCs *in vitro* was examined. The levels of secreted IFN-α and IFN-β were significantly enhanced in ORG and Lipo-ORG groups (Fig S16).

After that, the EGFP-mRNA transfection efficiency of anionic Lipo-ORG in BMDCs was evaluated (Fig. S17). Commercial SM-102 and MC3 (DLin-MC3-DMA) lipid nanoparticles were set as a positive control. ORG and anionic Lipo-ORG groups significantly improved the population of GFP^+^ cells to 40.1 ± 2.2% and 42.76 ± 4.51%, respectively. The SM-102 and DLin-MC3-DMA LNPs triggered successful transfection of mRNA in cells, but was weaker than ORG or Lipo-ORG. To validate the ability of anionic Lipo-ORG for presenting antigens, OVA-encoded mRNA was used. We treated BMDCs with various formulations for 24 h and detected the frequency of SIINFEKL-H-2K^b+^ (Fig. 4d). BMDCs treated with free OVA-encoded mRNA and cGAMP failed to induce effective antigen presentation, while OR nanocomplexes, ORG nanocomplexes and anionic Lipo-ORG significantly upregulated antigen-presenting efficiency. Besides, we noted that ORG nanocomplexes induced higher presentation efficacy than OR nanocomplexes, indicating that STING activation could facilitate the antigen presentation in BMDCs.

To test the immune stimulatory effect via delivery of mRNA and cGAMP, we immunized C57BL/6 mice with the anionic Lipo-ORG loading OVA-encoded mRNA and cGAMP at identical doses (mRNA: 0.5 mg/kg, cGAMP: 0.5 mg/kg). Then we harvested lymph nodes at indicated time points after mice received a single injection and measured STING-activated genes (Fig. 4e). Lipo-ORG significantly improved the levels of CXCL-10, Isg15 and IFN-β and exhibited the highest fold-change following injection for 1 day (Fig. 4f). Since the STING pathway was involved in early innate immunity activation, gene expression decreased as the time passed (Fig. S18a). The normalized levels of IFN-α and IFN-β were enhanced by Lipo-ORG, but not obviously improved in other groups (Fig. S18b and c). Additionally, we also evaluated the immune responses *in vivo* (Fig. S19). It was found that anionic Lipo-ORG effectively promoted the expression of CD80, CD86 on DCs (Fig. 4g). The population of CD80^+^CD86^+^ DCs with treated anionic Lipo-ORG was higher than that with PBS and ORG, respectively (Fig. 4g). Anionic Lipo-ORG showed higher performance than Lipo-OR, indicating that the addition of cGAMP was helpful for DCs maturation. This could be associated with cGAMP-mediated STING activation via Lipo-ORG. The population of MDSCs in tumours was significantly reduced in Lipo-ORG treated mice in contrast to the PBS group (Figs S20a and S21). The M1/M2 macrophage ratio was barely changed in R + G and ORG groups *in vivo*, while it was significantly enhanced in Lipo-ORG groups (Fig. 4h, Fig. S20b and c), suggesting that Lipo-ORG reversed the immunosuppressive macrophage TME. We investigated its ability to elicit epitope-specific T cells in C57BL/6 mice. Epitope-specific T cell responses and immune cellular compositions in splenocytes and tumour infiltrated lymphocytes were carried out on day 7. Typically, the highest frequency of OVA-specific CD8^+^ T cells were generated in the anionic Lipo-ORG group, which presented a 1.8-fold increase than ORG nanocomplexes, and 1.5-fold higher than that of the Lipo-OR group (Fig. 4i). We proceeded to examine whether the tumour-specific CD8^+^ T cells were able to infiltrate into tumour tissues. Consequently, most effective elicitation of OVA-specific CD8^+^ T cells in tumours were triggered by anionic Lipo-ORG, while mice vaccinated with free mRNA + cGAMP or ORG nanocomplexes presented no significant difference (Fig. 4j). Remarkably, Lipo-ORG triggered more OVA-specific T cells than that of Lipo-OR, which was associated with the activation of STING pathway by cGAMP to potentiate T cell responses (Fig. 4j). In the meanwhile, it showed that anionic Lipo-ORG induced notable elevation of CD8^+^ T cells (Fig. 4k). The population of CD8^+^ T cells increased from 33.5% in the PBS group to 58.6% after treatment. The CD8^+^ to CD4^+^ T cell ratio was also increased in the anionic Lipo-ORG treated group (Fig. 4l). In addition, the frequency of IFN γ^+^CD8^+^ T cells was dramatically increased due to effective cytosolic delivery of mRNA and cGAMP by anionic Lipo-ORG (Fig. 4m, Fig. S20d). The frequency of Tregs (gated on CD4^+^) was 20.3 ± 3.6% in PBS treated mice, while reduced to 8.1 ± 2.0% with Lipo-ORG treatment (Fig. 4n, Fig. S20e). To further elucidate changes of the whole mRNA expression and immune-related signaling pathways in tumours, RNA-Sequencing was employed on day 7 after various treatments (Fig. 4o, Fig. S22). In selected genes, we discovered around a 2-fold increase of gene expression in mice treated with anionic Lipo-ORG versus the PBS group. Moreover, these changed genes were further analyzed by Kyoto Encyclopedia of Genes and Genomes (KEGG) pathway to determine the upregulated and downregulated pathways (Fig. 4p).

### Antitumour study in tumour models and combinatorial regimen with PD-L1 blockade

**Figure 5.**
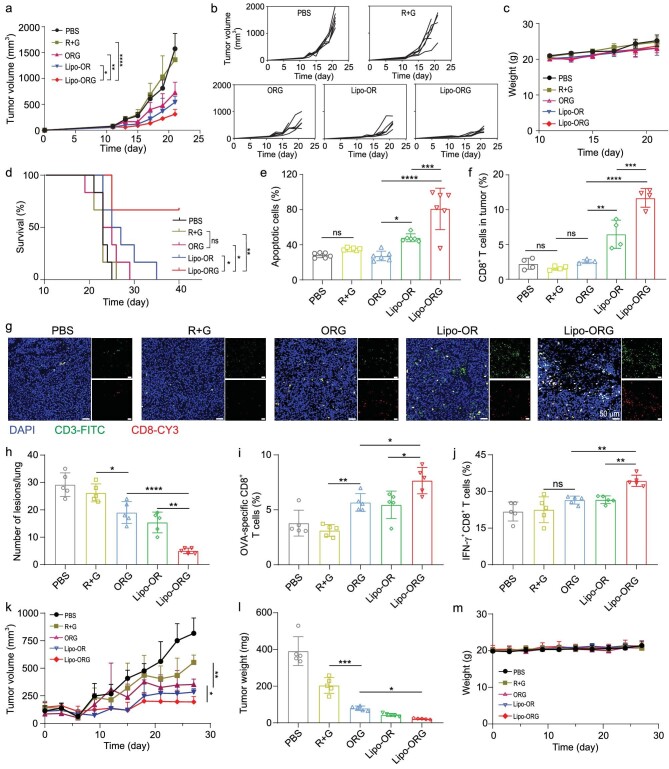
STING-activated LNP vaccine inhibited tumour growth and prolonged survival. (a) Average tumour volume was measured in B16-OVA tumour-bearing C57BL/6 mice (*n* = 6) with treatment of LNP vaccines and other control formulations on day 11 and day 17 after tumour inoculation. (b) Individual tumour growth curves in mice with the same treatment in a (*n* = 6). (c) Body weight of mice was measured during the treatment (*n* = 6). (d) Survival curves of B16-OVA tumour-bearing mice (*n* = 6) with different vaccination. (e) Quantification of apoptotic cells frequency in tumours (*n* = 6) as measured by image j. (f) Quantitative analysis of CD8^+^ T cells frequency (f) in B16-OVA tumours (*n* = 4), and (g) immunofluorescent staining of CD3^+^ T cells and CD8^+^ T cells, scale bars, 50 μm. (h) Anti-metastasis study on lung metastatic B16-OVA tumour model. The number of metastatic lesions were counted at the end of the study (*n* = 5). (i) OVA-tetramer positive CD8^+^ T cells, and (j) IFN-γ^+^ CD8^+^ T cells in LNs of mice from the anti-metastasis study (*n* = 5). (k) Average growth kinetics of subcutaneously inoculated MC38-OVA tumours after various treatments (*n* = 5). (l) Average weight of collected MC38-OVA tumour tissues in control and treated groups at the end of antitumour study (*n* = 5). (m) Body weight of MC38-OVA tumour-bearing mice during the study (*n* = 5).

Inspired by robust immune responses after vaccination, we evaluated the *in vivo* therapeutic effects of the anionic Lipo-ORG using a B16-OVA tumour-bearing mouse model. The mice were vaccinated when tumour volume reached ∼50 mm^3^, with subcutaneous injections twice at an interval of 5 days. The tumour volume, body weight and survival of each mouse were monitored during the process (Fig. 5a–d). Lipo-ORG significantly delayed the growth of tumours in mice in contrast to other groups (Fig. 5a and b). Mouse body weight was negligibly affected by treated suspensions (Fig. 5c), and no obvious injuries were found in major organs during the study (Fig. S23). In PBS and mRNA + cGAMP groups, all mice died within 25 days, and ORG nanocomplexes did not exhibit any obvious superiority in the benefit of mouse survival. In contrast, mice receiving anionic Lipo-ORG showed the most durable therapeutic efficacy and significantly slowed tumour progression with 67% of mice surviving over 40 days (Fig. 5d). Consequently, antitumour performance was further evaluated by hematoxylin-eosin (H&E) assay and terminal deoxynucleotidyl transferase dUTP nick-end labeling (TUNEL) staining at the end of the study (Fig. 5e and f, Fig. S24). Shrunken cell nucleus and leaked cytoplasm were observed in tumours post Lipo-ORG administration, suggesting effective apoptosis and necrosis of tumour cells upon immunization with T cells. The infiltration of CD8^+^ T cells in tumours was also evaluated by immunofluorescence staining (Fig. 5g). Few CD8^+^ T cells were observed in PBS, free drugs and ORG nanocomplexes groups, while plenary signals were observed after Lipo-ORG treatment. The results suggested that mRNA- and cGAMP-loaded Lipo-ORG effectively altered the immune TME to improve antitumour effects. We also evaluated the anti-metastasis efficiency of Lipo-ORG in lung metastatic B16-OVA mouse model. The results showed that Lipo-ORG did not induce significant decrease of body weight when compared to the PBS group (Fig. S25a). Lipo-ORG significantly reduced the growth of lung metastatic lesions with an average number of 5.0 ± 1.0 per lung. However, the average number of metastatic lesions in the PBS group was as high as 29.2 ± 4.3 per lung (Fig. 5h). The results indicated that Lipo-ORG could not only inhibit the growth of subcutaneous tumour, but also suppress the lung metastasis in a mouse model. To clarify the mechanism of anti-metastasis effect via immune responses, we detected the populations of OVA-specific CD8^+^ and IFN-γ^+^CD8^+^ T cells in spleen and draining lymph nodes of mice (Fig. 5i and j, Fig. S25b and c). The populations of OVA epitope–specific CD8^+^ and IFN-γ^+^CD8^+^ T cells in spleen and lymph nodes was improved by Lipo-ORG treatment, indicating that Lipo-ORG mobilized the functions of CD8^+^ T cells to inhibit tumour metastasis. To further expand the use of Lipo-ORG in other types of tumours, a subcutaneously inoculated MC38-OVA tumour model was established in mice. After various treatments, the growth of MC38-OVA tumours was monitored (Fig. 5k, Fig. S25d). Compared with other groups, most effective growth inhibition of MC38 tumours was observed the in Lipo-ORG treated group within 27 days. Besides, the significant differences of tumour weight among Lipo-ORG and other groups also showed the superior antitumour capability of Lipo-ORG in an MC38 tumour model (Fig. 5l). The suspensions did not induce body weight loss when compared to the PBS treated group (Fig. 5m). To find the key factors associated with good antitumour effect, we examined the immune responses in MC38 tumours. The population of epitope-specific T cells was elevated by Lipo-ORG treatment (Fig. S25e). Besides, the frequency of IFN-γ^+^ CD8^+^ T cells was also increased in the Lipo-ORG group (Fig. S25f). Compared to the PBS group, the CD8^+^ to CD4^+^ T cell ratio was enhanced by Lipo-OR and could be higher with Lipo-ORG (Fig. S25g). Consistent with the results in the B16-OVA tumour model, the immunosuppressive factors in tumours including macrophages and MDSCs were suppressed after treatment by Lipo-ORG (Fig. S25h and i).

**Figure 6.**
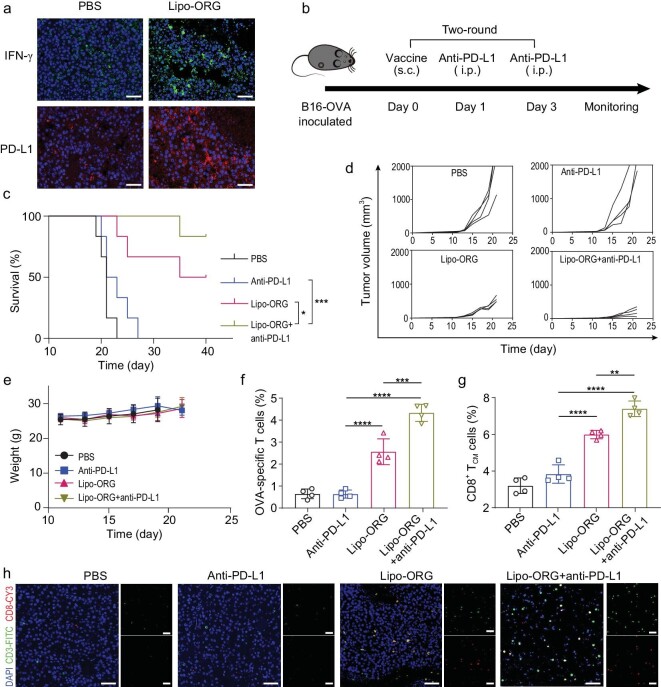
STING-activated LNP vaccine combined with PD-L1 blockade for improved cancer immunotherapy and long-term memory effect. (a) Immunofluorescent analysis of IFN-γ (green) and PD-L1 (red) expression in B16-OVA tumours from mice treated with PBS or Lipo-ORG. Scale bars, 50 μm. (b) Schematic illustration of treatment schedule on B16-OVA tumour-bearing mice when combined with anti-PD-L1 antibody. Mice were treated with anti-PD-L1 antibody on day 1 and day 3 after every vaccination, and the treatment regimen was performed twice. (c) Survival curves of tumour-bearing mice with treatment of PBS, anti-PD-L1 antibody, Lipo-ORG, and Lipo-ORG + anti-PD-L1 (*n* = 6). (d) Individual tumour growth curve in mice (*n* = 4) with the same regimen. (e) Body weight of mice during the treatment (*n* = 6). (f and g) Frequency of OVA-specific T cells (OVA-tetramer^+^ CD8^+^ T cells) (f) and central memory T cells (T_CM_, CD44^+^ CD127^+^ CD8^+^ T cells) (g) in splenocytes were determined on day 7 post vaccination (*n* = 4). (h) Representative immunofluorescent images of CD3^+^ T cells (green) and CD8^+^ T cells (red) in B16-OVA tumours. Scale bars, 50 μm.

Since the population of IFN-γ^+^CD8^+^ T cells was elevated with Lipo-ORG treatment, we tested the secretion of IFN-γ in tumours. Increased signals of IFN-γ were found in the Lipo-ORG group compared to PBS group (Fig. 6a). It has been reported that the expression of PD-L1 was a dynamic process associated with the level of IFN-γ. We confirmed that PD-L1 was overexpressed in tumour tissues after treatment by Lipo-ORG in contrast to the PBS group. The immune activation of Lipo-ORG in established tumours could be hampered by the PD-1/PD-L1 pathway. Therefore, we considered the combinatorial regimen of anionic Lipo-ORG with PD-L1 inhibitor to further enhance the antitumour efficacy. The B16-OVA bearing mice were vaccinated on day 0 and treated by anti–PD-L1 antibodies on day 1 and day 3, respectively. The treatments went through a two round process and mouse survival was monitored (Fig. 6b). It was found that anti–PD-L1 alone had minimal effect for prolonging mouse survival, while the combination regimen significantly extended the survival duration (Fig. 6c). The combinatorial regimen also inhibited the growth of tumours in mice (Fig. 6d). No obvious weight loss was observed during the treatments (Fig. 6e). The results of H&E assay and TUNEL determination in tumour sections further confirmed the therapeutic efficacy of a combination regimen (Fig. S26). We further examined the OVA-specific and central memory T cells in splenocytes on day 21 post administration. As expected, the combination therapy induced a remarkable increase of OVA-specific CD8^+^ T cells, showing 6.8- and 1.7-fold to the anti–PD-L1 group and Lipo-ORG group, respectively (Fig. 6f, Fig. S27a). Additionally, the proportion of central memory T cells in CD8^+^ T cells were also generated (Fig. 6g, Fig. S27b), verifying activation of immune memory effect for long-lasting tumour inhibition. As we detected the infiltrated lymphocytes in tumour tissues via immunofluorescence assay, an increased number of CD8^+^ T cells was detected in tumour sections that had received the combinatorial treatment (Fig. 6h). Altogether, the results indicated that Lipo-ORG–mediated immune activation cooperating with PD-L1 blockade was a potent strategy for improving antitumour performance.

## CONCLUSION

We established a nanovaccine based on machine learning techniques to overcome multistage barriers for delivering mRNA and other nucleic acid drugs to lymph nodes. We have shown that the nanovaccine could activate potent antitumour immunity and long-term memory effects through delivering mRNA antigen and STING agonist, cGAMP (Fig. S28a). Key parameters of the nanovaccine were optimized according to dataset analysis. We found that modification of PBA moiety on OEI dramatically promoted transfection efficiency, and negatively charged Lipo-ORG was preferred for lymphatic delivery. In practice, we found that the anionic Lipo-ORG was able to suppress tumour growth, metastasis and extend survival in the B16-OVA tumour model, in which the efficacy is further amplified by synergizing with PD-L1 blockade. In particular, the utilization of STING agonist, cGAMP, showed great potential in tumour immunotherapy by enhancing tumour-specific CD8^+^ T cell responses. Despite the observed robust immune effects, clinical translation has been limited due to insufficient cytosol delivery. Therefore, the nanovaccine provided a facile and efficient platform for delivering nucleotide drugs, but also a promising therapeutic strategy for clinical translation. In addition to delivering OVA-encoded mRNA to treat modeling tumours, other kinds of neoantigens-encoded mRNA could also be applied for personalized cancer immunotherapy. Moreover, beyond vaccination against cancer, the nanovaccines are able to cure various diseases by formulating with indicated nucleotide drugs. In future studies, the nanovaccine designs may be optimized through machine learning based on a nanovaccine library (Fig. S28b). This study provides a feasible and flexible strategy for highly efficient cytosolic delivery of mRNA, which is promising for clinical translation of STING activation-promoted mRNA vaccination.

## Supplementary Material

nwad214_Supplemental_File
