# Supplementary material for: STING agonist-boosted mRNA immunization via intelligent design of nanovaccines for enhancing cancer immunotherapy
Source: Natl Sci Rev. 2023 Aug 11;10(10):nwad214. doi: 10.1093/nsr/nwad214 (PMC10484175; doi:10.1093/nsr/nwad214)
Supplement: nwad214_Supplemental_File [file nwad214_supplemental_file.zip › Supplementary data.pdf]

Supplementary Information for

STING agonist-boosted mRNA immunization via intelligent design of nanovaccines for enhancing cancer immunotherapy

Lei Zhou[1,3,]†, Wenzhe Yi[1,5,]†, Zehong Zhang[4,5,]† , Xiaoting Shan[1,5], Zitong Zhao[1], Xiangshi Sun[1], Jue Wang[1,5], Hao Wang[3], Hualiang Jiang[4,5], Mingyue Zheng[4,5,]*, Dangge Wang[2,]* and Yaping Li[1,5,6,]*

[1]State Key Laboratory of Drug Research & Center of Pharmaceutics, Shanghai Institute of Materia Medica, Chinese Academy of Sciences, Shanghai 201203, China;

[2]Precision Research Center for Refractory Diseases, Shanghai General Hospital, Shanghai Jiao Tong University School of Medicine, Shanghai, 201620, China;

[3]China State Institute of Pharmaceutical Industry, Shanghai 201203, China;

[4]Drug Discovery and Design Center, State Key Laboratory of Drug Research, Shanghai Institute of Materia Medica, Chinese Academy of Sciences, Shanghai 201203, China;

[5]Univerisity of Chinese Academy of Sciences, Beijing 100049, China;

[6]Shandong Laboratory of Yantai Drug Discovery, Bohai Rim Advanced Research Institute for Drug Discovery, Shandong 264000, China

***Corresponding authors.** E-mails: myzheng@simm.ac.cn; dgwang@shsmu.edu.cn; ypli@simm.ac.cn

†Equally contributed to this work.

Methods

Materials, cells and animals. OEI (Mn = 1800 Da, Mw = 2000 Da) were obtained from Sigma-Aldrich (Shanghai, China). 4-(bromomethyl)-phenylboronic acid were purchased from J&K Scientific Ltd (Beijing, China). EGFP-mRNA and OVA-mRNA were purchased from Trilink BioTechnologies (San Diego, USA). cGAMP was obtained from InvivoGen (Toulouse, France), while cdGMP-DY547 was purchased from BIOLOG life science institute (Bremen, Germany). Anti-PD-L1 was obtained from Bioxcell (USA). DAPI, lysotracker (green) were obtained from Beyotime Co., Ltd (Jiangsu, China). DiR was purchased from Meilun Biotech. Co., Ltd (Dalian, China). Cell counting kit-8 (CCK-8), Trizol reagent, Hifair® Ⅱ 1st Strand cDNA Synthesis SuperMix and Hieff UNICON® qPCR SYBR Green Master Mix were all obtained from Yeasen (Shanghai, China). All the lipids, like DOPE, DOTAP, DSPG, DSPE-PEG, DPPC and DSPC, were all ordered from A.V.T. Pharmaceutical Co., Ltd. (Shanghai, China). The SM-102 and DLin-MC3-DMA lipids were gained from Csnpharm Co., Ltd. The primers of CXCL 10, Isg 15, GAPDH, IFN-β (mouse, human) were all custom ordered from Sangon Biotech (Shanghai, China). SIINFEKL-H2K^b-PE antibody was purchased from Dakewe Biotech (Beijing, China). OVA-tetramer-PE antibody was ordered from MBL Beijing Biotech Co., Ltd. (Beijing, China). Other antibodies for flow cytometry analysis, including anti-CD45-APC, anti-CD11c-FITC, anti-CD80-PE, anti-CD86-PE-Cy7, anti-MHC II-PercP Cy5.5, anti-CD3-PercP Cy5.5, anti-CD4-FITC, anti-CD8-PE, anti-CD8-FITC, anti-IFN-γ-FITC, anti-Foxp3-PE, anti-CD44-PerCP Cy5.5, anti-CD127-PE and fixation-permeabilization concentrate buffer were all purchased from BD Biosciences (USA).

Murine B16-OVA melanoma cells, MC38-OVA colorectal cancer cells, RAW 264.7 cells and THP-1 cells were obtained from the cell bank of the Chinese Academy of Sciences (Shanghai, China). DC2.4 cells were purchased from ShunRan Biotech (Shanghai, China). C57BL/6 mice (female, 4-6 weeks old) were purchased from Shanghai Experimental Animal Center (Shanghai, China). All animal procedures were carried out under the guidelines approved by the Institutional Animal Care and Use Committee (IACUC) of the Shanghai Institute of Material Medica, Chinese Academy of Sciences.

Data collection and processing. We collected two datasets from previous publications: cytosolic delivery dataset and LN- delivering dataset. Then we deleted the data without transfection efficiency values of the cytosolic delivery dataset. Finally, the cytosolic delivery dataset covered 471 transfection efficiency measurements of nanocarriers and the LN-delivering dataset covered 158 particles which were able to deliver drugs to LNs.

Because our data sets were small, it was not reasonable to simply apply the delete strategy to control the missing value problems, which would lose a lot of useful information. MICE (Multivariate Imputation by Chained Equations) in R was a simple and easy-to-use method, which had been proved to be a good missing data handling strategy. We used MICE to impute missing feature values in our data sets through an iterative series of predictive models. In each iteration, each specified variable in the dataset is imputed using the other variables in the dataset. Some missing feature values had constraints (positive or negative). In every imputing processing, those interpolations that failed to meet the constraints were removed and re-imputed until all interpolation values met the constraints. In the data, there

are some features that cannot be simply considered as numerical values, such as cell type and category, which are processed by one-hot coding.

Machine learning techniques for data analysis. We cast the task of predicting the transfection efficiency values of nanocarriers as a classification problem, and built 4 machine learning models for this task (SVC, RF, LightGBM and XGBoost). These models received input features (particle size, ζ potential, stimuli response, and so on) of the nanocarriers and produced the predicted transfection efficiency classes of particles as output. To select the hyperparameters of these models, we performed a random search using five-fold cross-validation for performance evaluation.

The SVC (support-vector classification) model represented instances as points in space, and outputted the hyperplane that best separates the tags. The new instances were then mapped to the same space and the category was predicted based on which side of the hyperplane they fell on. RF (random forest) was to establish a decision tree forest in a random way. There were many decision trees in the forest, and there was no correlation between each decision tree in the random forest. After the forest was obtained, when a new input sample enters, each decision tree in the forest was asked to make a judgment respectively to see which category this sample should belong to, and then see which category was selected the most, and predict this sample to be that category. XGBoost and lightGBM were both algorithms that promote weak learners to strong learners, and they work in a similar way: First, a base learner was trained from the initial training set, and then the distribution of training samples was adjusted according to the performance of the base learner, so that the samples classified incorrectly by the previous base learner receive more attention, and then

the next base learner was trained based on the adjusted sample distribution. Finally, the weighted summation of all base learners was performed.

Performance evaluation metrics. When performing classification predictions, there were four types of outcomes that could occur. True positives (TP) were when we predicted an observation belonged to a class and it actually did belong to that class; true negatives (TN) were when we predicted an observation did not belong to a class and it actually did not belong to that class; false positives (FP) occurred when we predicted an observation belongs to a class but in reality it did not; false negatives (FN) occurred when we predict an observation did not belong to a class but in fact it did.

The models were scored on the validation set using precision, recall, F1, AUC and kappa. Precision was defined as the fraction of true positives among all examples which were predicted to belong in a certain class.

$$Precision = \frac{TP}{TP + FP}$$

Recall was defined as the fraction of examples that were predicted to belong to a class with respect to all of the examples that truly belong in the class.

$$Recall = \frac{TP}{TP + FN}$$

F1 score was the harmonic mean of precision and recall.

$$F1 = \frac{2 * Precision * Recall}{Precision + Recall}$$

AUC (Area Under the ROC Curve) measured the entire two-dimensional area underneath the entire ROC curve. One way of interpreting AUC was the probability that the model ranked a random positive example more highly than a random negative example. If AUC was greater

than 0.5, the model was considered to have certain predictive ability. The closer the AUC was to 1, the stronger the predictive ability was.

The Kappa coefficient (κ) was used to measure the consistency between predicted classification and real classification, and the definition of κ was:

$$\kappa = \frac{p_o - p_e}{1 - p_e} = 1 - \frac{1 - p_o}{1 - p_e}$$

where p_o was the observed agreement, and p_e was the expected agreement. If κ was 0 or less, it indicated that the classifier was useless. If κ value was greater than 0, it indicated that the model had certain classification performance. The closer κ was to 1, the closer the model prediction result was to the real value.

Multi-classification was more complicated than binary classification. It required calculating mean evaluation metric values of different categories and get the final evaluation metric values of the whole. According to averaging methods, it can be divided into macro average and micro average. Macro averaging reduced multiclass predictions down to multiple sets of binary predictions, calculated the corresponding metric for each of the binary cases, and then averaged the results together. As an example, consider macro-average precision for triple classification tasks.

$$macro\ P = \frac{P_{class\ 1} + P_{class\ 2+} + P_{class\ 3}}{3}$$

Micro averaging treated the entire set of data as an aggregate result, and calculated 1 metric rather than k metrics that get averaged together. As an example, consider micro-average precision for triple classification tasks.

$$micro\ P = \frac{TP_{class\ 1} + TP_{class\ 2+} + TP_{class\ 3}}{(TP_{class\ 1} + TP_{class\ 2+} + TP_{class\ 3}) + (FP_{class\ 1} + FP_{class\ 2+} + FP_{class\ 3})}$$

In particular, precision, recall and F1 were equal under the micro averaging method.

$$micro\ P = micro\ R = micro\ F1$$

The macro average method was greatly influenced by the unbalanced category, while the micro average method was not. Therefore, we selected the model mainly according to the micro average metrics.

Computing feature-importance estimates. The interpretability of machine learning model has obvious practical significance, which can assist experts to make decisions and help the further development of the subject. We used SHAP to fairly identify the principal features driving model prediction. SHAP was a method to explain individual predictions, which was based on the game theoretically optimal Shapley Values. The SHAP value can be calculated by the following formula：

$$g(z') = \varphi_0 + \sum_{j=1}^{M} \varphi_j z'_j$$

where g was the explanation model, M was the number of input features, z indicated whether the corresponding feature exists (1 or 0) and $\varphi_j \in \mathbb{R}$ was the feature attribution for a feature j, the Shapley values. As the input of the tree model was structured data, all features exist for sample x, so the formula can be simplified as:

$$g(z') = \varphi_0 + \sum_{j=1}^{M} \varphi_j$$

By averaging the samples, we used SHAP values to estimate the contribution of each feature to the overall model prediction. And we used SHAP values to find important features, the idea was simple: features with large absolute Shapley values were important.

One-class SVM for lymph node delivery. Since all the collected data of lymph node delivery was positive, one-class SVM was used for verifying whether our nanocarriers could

deliver drugs to lymph nodes. The question of whether nanocarriers can deliver drugs to lymph nodes is whether the characteristics of nanocarriers are within the range of lymph node delivery data in literature. We selected the upper bounds on the fraction of training errors of 5%, 10%, 20% and 40% for experiments, that is, the most marginal 5%, 10%, 20% and 40% of all the data were outliers. As long as our nanocarrier was within the region of the model, we believed that the carrier was highly likely to have lymph node delivery capability.

Synthesis of OEI-PBA and PAMAM-PBA. The cationic polymer OEI-PBA was synthesized by reacting OEI with PBA according to described protocols. Briefly, the solution of OEI and PBA were mixed together at various molar ratio (1:2, 1:4 and 1:8) in methanol and kept stirring at 50℃ oil bath for 24 h. Then the reaction was precipitated in diethyl ether for three times and dried under vacuum evaporation. Finally, the products were characterized by ^{1}H-NMR spectra. A similar method was used to synthesize PAMAM-PBA by incubating PAMAM (G2) with different molar ratios with PBA (1:2, 1:4 and 1:8), and characterized by ^{1}H-NMR spectra.

Preparation of OEI-PBA/mRNA nanocomplexes. Firstly, to determine mRNA complexation ability of cationic materials, naked mRNA was incubated with OEI or OEI-PBA at different polymer to mRNA weight ratios (1: 0.125, 0.25, 0.5, 1) for 15 minutes. Considering the organic solvent (methanol) in OEI-PBA solution, naked mRNA with methanol was set as control group to test the stability of mRNA in organic solvent. All these samples were added with loading buffer and run in 1% agarose gel at 80 V for 1 h. After that, the gel was imaged and analyzed. For transfection evaluation, DC2.4 cells were seeded at a density of 8×10^4 per well in the 24-well plate for overnight attachment. Free EGFP-mRNA

(0.5 μg/mL), as well as OEI or OEI-PBA complexed with EGFP-mRNA at varying weight ratios (5:1, 10:1, 20:1, 40:1, 60:1, 80:1, 100:1) were inoculation with cells in serum-reduced Opti-medium for 4 h, then fresh complete medium was added for further incubation. After 24 h, cells were collected for analyzing GFP protein expression using flow cytometry. The same methods were applied to investigate mRNA transfection efficiency of PAMAM-PBA/mRNA nanocomplexes.

Cell culture and cytotoxicity in vitro. BMDCs were derived from bone marrow monocytes, flushed from the femurs of C57BL/6 mice, with 20 ng/mL GM-CSF and 10 ng/mL IL-4 stimulation. The medium was half replaced every two days with sufficient cytokines supplement. On day 6, the non-adherent and loosely adherent cells BMDCs were collected for further experimental use. All the cell lines were cultured in RPMI 1640 with high-glucose, supplementing with 10% FBS and 100 U/mL of penicillin and 100 μg/mL of streptomycin. Then cells were maintained at a humidified atmosphere with 5% CO_2 at 37 ℃. For cell cytotoxicity assay, DC2.4 cells were seeded in 96-well plate at a density of 5×10^3 per well. Cells were treated with various formulations at a series of concentrations in the next day. After 24 h, CCK-8 kit was added into the medium and the absorbance was measured at 450 nm after 2 h incubation.

Preparation and characterization of Lipo-ORG. Firstly, the cationic nanocomplexes of OEI-PBA/mRNA/cGAMP (ORG) was fabricated. In brief, the OEI-PBA solution was mixed with mRNA and cGAMP for 15 minutes, following with ultrasonication to prepare the uniform nanoparticles. To get liposomes with different surface charges (cationic liposomes, DOPE, DOTAP, DSPE-PEG (molar ratio 1:2.13:0.54); anionic liposomes, DOPE, DSPG,

DSPE-PEG (molar ratio 1:1.86:0.54); neutral liposomes, DOPE, DSPC, DPPC (molar ratio 1:1.88:2.03)), the lipid compositions were selected to mix together to form thin lipid films under rotoevaporation for reagent removal. Then the ORG nanoparticles solution was added to hydrate the film. The resulting mixture was further dispersed under ultrasonication to obtain the mRNA-loaded lipid nanoparticles. The size distribution and surface charge of ORG, as well as three kinds of Lipo-ORG were all determined by DLS measurements. TEM and cryo-TEM were also performed to image the morphology. The content of cGAMP was measured by ELISA kit according to manufacture protocol (Cayman Chemical Company, USA). We also prepared positive controls with commercial SM-102 and DLin-MC3-DMA lipids. The LNPs were fabricated by lipid compositions including SM-102, DSPC, cholesterol and DSPE-PEG2000 (weight ratio 50:10:38.5:1.5). The weight ratio between LNPs and mRNA was 40:1, as same as that of Lipo-ORG. DLin-MC3-DMA (MC3) LNPs were fabricated in the same method by replacing SM-102 with DLin-MC3-DMA.

Cellular uptake and antigen presentation. To determine the ability of co-delivering EGFP-mRNA and cGAMP in vitro, DC2.4 cells were seeded in 24-well plate at a density of 8×10^4 per well and incubated overnight to allow cells to attach. Next day, cells were treated with formulations containing both EGFP-mRNA (0.5 μg/mL) and cdGMP-DY547 (1 μg/mL) for 24 h. Cells were then collected and analyzed by flow cytometry to detect EGFP expression and cdGMP-DY547 signals in cells. For confocal examination, cells were plated on the coverslips and operated as above protocols. The coverslips were mounted onto slides for CLSM observation after DAPI staining. To assess antigen presentation efficiency of the Lipo-ORG, BMDCs were prepared and plated at 24-well plate at an initial density of 5×10^5

per well. Meanwhile, OVA-mRNA and cGAMP in various formulations were added into medium and incubated with BMDCs for 24 h. Then, BMDCs were collected for analysis. We evaluated the secretion of type I IFN in BMDCs in vitro. The BMDCs were seeded in 12-well plate at a concentration of 8×10^5/mL overnight. Then, the BMDCs were treated with R+G, ORG and Lipo-ORG at equal level of 0.5 μg/mL cGAMP for 24 h. The secreted IFN-α and IFN-β in cell culture medium were determined by ELISA kits.

Lymphatic transport of Lipo-ORG and APCs' uptake in vivo. For biodistribution experiments, three kinds of Lipo-ORG encapsulating DiR (200 μg/mL) were injected subcutaneously. 24 h and 48 h post administration, mice were monitored by IVIS (Ex/Em: 748/780 nm). Then, lymph nodes were resected, likewise imaged ex vivo at the same time. To investigate the uptake of Lipo-ORG by APCs in vivo, the lymph nodes were further digested into single cells and stained with appropriate antibodies to identify the cell populations. For example, anti-CD45-APC, anti-CD11c-PE and anti-MHC II-Percp Cy5.5 were applied to recognize DCs. Anti-CD45-APC, anti-CD11b-PE, anti-F4/80-PE-Cy7 were used to detect the uptake in macrophages. To assess mRNA and cGAMP co-delivery in vivo, EGFP-mRNA (0.5 mg/kg) and cdGMP-DY547 (0.25 mg/kg) were employed in various formulations. Mice were injected subcutaneously and lymph nodes were harvested according to the above operations. The EGFP and DY547 signals in both DCs and macrophages were analyzed by flow cytometry, respectively. The co-localization of mRNA and cGAMP was also examined in the frozen section of lymph nodes though multiplex IHC CLSM.

Anti-tumor study in tumor models. C57BL/6 mice were subcutaneously injected with B16-OVA tumor cells (5×10^5 per mouse) at the right flank. The tumor-bearing mice were

randomly divided into five groups when tumor volume reached at about 50 mm^3: (1) PBS; (2) Free mRNA + cGAMP; (3) ORG; (4) Lipo-OR; (5) Lipo-ORG (OVA-mRNA: 0.5 mg/kg; cGAMP: 0.5 mg/kg). Mice were subcutaneously injected with the above formulations every 5 days for twice. Tumor size as well as body weight were measured every two days. For anti-metastasis study, 3×10^5 B16-OVA cells were intravenously injected into C57BL/6 mice via tail vein on day 1. Then, the mice were administrated with PBS, R+G, ORG, Lipo-OR and Lipo-ORG at equal dosages of OVA-mRNA and cGAMP (OVA-mRNA: 0.5 mg/kg, cGAMP: 0.5 mg/kg) for 3 times on day 2, day 5 and day 8, respectively. The body weight of mice was monitored, and lungs in control and treated groups were collected on day 22. Similarly, subcutaneously inoculated MC38-OVA tumor model was established to evaluate antitumor efficiency of Lipo-ORG. The growth of tumors and body weight of mice were monitored. For the combination therapy with immune checkpoint blockade, mice in desired groups were intraperitoneally injected with anti-PD-L1 antibody at a dose of 5 mg/kg on day 1 and day 3 after each Lipo-ORG vaccination. Tumor growth was monitored as indicated above, and mice in the therapeutic experiments were euthanized before the volume reached 2000 mm^3.

Immune responses in vivo. To investigate the immune response triggered by the Lipo-ORG, B16-OVA or MC38-OVA tumor-bearing mice were vaccinated with varying formulations. The immune cells were examined with exclusion of dead cells following two steps. Firstly, the cells were gated as a function of SSC-A and FSC-A values. Most of the cell fragments were excluded in this step. Then, the living cells were gated out by Zombie UV™ Fixable Viability Dye staining. The amine reactive fluorescent dye was non-permeant to living cells while permeant to dead cells very well. Hence, immune cells with exclusion of dead cells were

gated with low zombie UV^{TM} levels. Lymph nodes were excised on day 5 and processed into single cell suspension. To determine DC maturation, cells were stained with antibodies of CD45-APC, CD11c-FITC, MHC II-PercpCy5.5, CD80-PE and CD86-PE-CY7. Matured DCs were analyzed by flow cytometry and gated as $CD45^{+}CD11c^{+}MHC\ II^{+}CD80^{+}CD86^{+}$. To verify the production of antigen-specific $CD8^{+}$ T cells, splenocytes were stained with CD8-FITC and PE-tagged OVA-MHC tetramer, following with flow cytometric detection. Flow cytometer experiments of immune responses were performed with fluorescence-minus-one (FMO) controls, including the assay of $IFN\text{-}\gamma^{+}CD8^{+}$ T cells, Tregs and macrophages in vivo. For analysis, the corresponding FMO controls were prepared without the staining of CD3, CD4, CD8, IFN-γ, Foxp3, CD86 or CD206 biomarker, respectively. The populations of biomarker positive cells were gated carefully with FMO controls. To determine intratumoral infiltrating T lymphocytes, tumor tissues were harvested for analysis. Briefly, tumors were cut into small pieces and then operated in gentle MACS Dissociator (Miltenyi, German) with RPMI160 medium and given enzymes according to manufacturer instructions. The single cell suspensions were acquired by 70 μm and 30 μm smart strainer filtration. Cells were stained with different panels of antibodies for recognition of various T cell population. Antibodies of CD45-APC, CD3-percpcy5.5, CD4-FITC and CD8-PE were stained to quantify the subpopulation of $CD4^{+}$ and $CD8^{+}$ T cells. In parallel, the cytotoxic T lymphocytes and regulatory T cells were also detected. For intracellular markers staining, like IFN-γ and Foxp3, the cells were firstly stained with surface markers, following with IFN-γ or Foxp3 staining after fixed and permeabilized. The populations of MDSCs, M1, M2 macrophages in tumors were also examined by anti-CD45, anti-CD11b, anti-Gr-1,

anti-F4/80, anti-CD86 or anti-CD206 antibodies, respectively.

Quantitative for STING-relevant genes and proteins expression. To investigate STING activation, the expression of relevant genes and proteins was detected in vitro and in vivo after treatment. Immature BMDCs were seeded in 6-well plate at a concentration of 8×10^5 per well, and treated by different suspensions for 24 h (0.5 μg/mL of mRNA, 0.5 μg/mL of cGAMP). Proteins were collected via cell lysate, normalized to equal concentration and performed with western blotting technique. Anti-TBK1 (clone: D184), anti-p-TBK1 (S172) (clone: D52C2), anti-IRF-3 (clone: D83B9) and anti-p-IRF-3 (S396) (clone: D601M) antibodies were used to label the proteins, and GAPDH was set as control. For qPCR, BMDCs, THP-1 and RAW were maintained and stimulated with various treatments. For in vivo study, lymph nodes and tumors were collected at indicated time points after administration. Then total RNA was extracted by trizol, and reverse transcribed into cDNA, and RT-PCR was performed and calculated by $2^{-\Delta\Delta Ct}$. The primers used in the experiments were listed as follows: GAPDH (mouse and human), Isg-15 (mouse and human), CXCL 10 (mouse and human), IFN-β (mouse and human).

	F	R
mCXCL10	CAACTGCATCCATATCGATGAC	GATTCCGGATTCAGACATCTCT
mIFN-β	CTGGGTGGAATGAGACTATTGT	AAGTTCCTGAAGATCTCTGCTC
mIsg15	AGCGAGCCTCTGAGCATCCTG	GCGTGTCTACAGTCTGCGTCAG
hCXCL10	TGCCATTCTGATTTGCTGCCTTATC	TGATGCAGGTACAGCGTACAGTTC
hIFN-β	GAAGGAGGACGCCGCATTGAC	ACAATAGTCTCATTCCAGCCAGTGC
hIsg15	CCTGCTGGTGGTGGACAAATGC	TGCGTCAGCCGTACCTCGTAG

Statistical analysis. One-way analysis of variance (ANOVA) or two tailed Student's t-test analysis was used to calculate statistical differences between groups in Graphpad Prism 7.0. *P*

< 0.05 was considered as significant, as well as other significant differences shown in figures were as follows: *$P < 0.05$, ** $P < 0.01$, *** $P < 0.001$, **** $P < 0.0001$. All the results were given as mean ± standard deviation (SD).

Supplementary Table 1. The summarized information of nanoparticles (n=258) for RNA delivery.

NO.	Ref.	Cell type	Transfection efficiency	Size (nm)	Surface charge (mV)	Category**(a)**	Modify	Stimuli-responsive
1	1	PTEN-Cap8 cells (tumor cell）	65%	111.8	Positive	2		
2	2	HeLa cells （tumor cell)	75%	179	25	2		
3		HeLa cells （tumor cell)	55%	80.6	10	2		
4		HeLa cells （tumor cell)	35%	129.5	2.5	2		
5		HeLa cells （tumor cell)	45%	100.4	22	2		
6		HeLa cells （tumor cell)	70%	212.2	20	2		
7		HeLa cells （tumor cell)	55%	151.8	10	2		
8	3	Hepa1-6 cells (tumor cell)	N/A	209.68	42.03	1		
9	4	DC2.4 cells (immune cell)	30%	100	27.28	1		
10	5	N/A	N/A	37.9	-28	3		
11	6	HEK293T.17 cells	N/A	250	-4	2		√
12		HEK293T.17 cells (other cell)	N/A	200	7	2		√
13		HEK293T.17 cells (other cell)	N/A	100	1	2		√
14		HEK293T.17 cells (other cell)	N/A	200	6	2		√
15		HEK293T.17 cells (other cell)	N/A	250	5	2		√
16	7	DC2.4 cells (immune cell)	25%	180	1	1	√	
17	8	PBMCs (other cell)	N/A	90	40	1		
18		PBMCs (other cell)	N/A	110	40	1		
19		PBMCs (other cell)	N/A	100	27	1		
20		PBMCs (other cell)	N/A	110	45	1		
21		PBMCs (other cell)	N/A	150	40	1		
22	9	BHK cells (other cell)	N/A	91.9	15.6	1		
23		BHK cells (other cell)	N/A	97.23	13.1	1		

24	10	BHK cells (other cell)	45%	198	26	1
25		BHK cells (other cell)	60%	85	27	1
26		BHK cells (other cell)	75%	267	26	1
27		BHK cells (other cell)	45%	196	43	1
28	11	Vero cells (other cell)	5%	616.9	-21.8	1
29	12	DC2.4 cells (immune cell)	30%	100	27.28	1
30	13	N/A	N/A	156.9	-1	3
31	14	HEK293T.17 cells (other cell)	N/A	180	8	1
32		HEK293T.17 cells (other cell)	N/A	185	-12	1
33		HEK293T.17 cells (other cell)	N/A	130	22	1
34		HEK293T.17 cells (other cell)	N/A	180	8	1
35		HEK293T.17 cells (other cell)	N/A	200	32	1
36		HEK293T.17 cells (other cell)	N/A	180	12	1
37	15	BHK-21 cells (other cell)	N/A	26	N/A	3
38	16	HEK293T.17 cells (other cell)	N/A	180	38	1
39		HEK293T.17 cells (other cell)	N/A	260	55	1
40		HEK293T.17 cells (other cell)	N/A	140	40	1
41		HEK293T.17 cells (other cell)	N/A	120	60	1
42		HEK293T.17 cells (other cell)	N/A	150	20	1
43		HEK293T.17 cells (other cell)	N/A	220	10	1
44		HEK293T.17 cells (other cell)	N/A	140	0	1
45	17	BHK cells (other cell)	44.90%	126	26.5	2
46	18	DC (immune cell)	60%	300	-30	1
47	19	HEK293T.17 cells (other cell)	N/A	180	34	2
48		HEK293T.17 cells (other cell)	N/A	160	30	2
49		HEK293T.17 cells	N/A	200	22	2

		(other cell)						
50		HEK293T.17 cells (other cell)	N/A	150	26	2		
51		HEK293T.17 cells (other cell)	N/A	150	27	2		
52		HEK293T.17 cells (other cell)	N/A	160	29	2		
53		HEK293T.17 cells (other cell)	N/A	150	25	2		
54		HEK293T.17 cells (other cell)	N/A	120	25	2		
55	20	HEK293T.17 cells (other cell)	24.30%	75	N/A	1		
56	21	Hek293 cells (other cell)	42%	52	15	2		
57	22	HeLa cells (tumor cell)	N/A	99.9	4.4	1		
58		HeLa cells (tumor cell)	N/A	87.9	4.9	1		
59	23	DC (immune cell)	N/A	200	50	1		
60	24	DC2.4 (immune cell)	60%	110	25	2		
61	25	human skin explants (other cell)	22%	140	31	2		
62		human skin explants (other cell)	32%	230	40	2		
63		human skin explants (other cell)	29%	130	30	2		
64		human skin explants (other cell)	30%	50	34	2		
65		human skin explants (other cell)	26%	125	28	2		
66		human skin explants (other cell)	25%	100	32	2		
67		human skin explants (other cell)	30%	50	30	2		
68	26	N/A	N/A	140-150	Positive	1	√	
69	27	DC2.4 cells (immune cell)	N/A	250	-20	1		
70		DC2.4 cells (immune cell)	N/A	150	16	2		
71	28	CD172a+ blood cells (other cell)	42.8%	400	-60	2		
72	29	DC2.4 cells (immune cell)	32.0%	280	40	1		√
73		DC2.4 cells (immune	26.0%	250	41	1		√

		cell)					
74	30	DC (immune cell)	40%	169	25.5	1	
75		DC (immune cell)	60%	154	24.6	1	
76		DC (immune cell)	21%	152	36.5	1	
77		DC (immune cell)	40%	137	17	1	
78	31	DC2.4 cells (immune cell)	35%	117.77	Positive	2	
79	32	DC2.4 cells (immune cell)	N/A	234.7	Positive	2	
80	33	NIH 3T3 (other cell)	N/A	115	N/A	4	
81	34	Vero cells (other cell)	N/A	41.79	-38.8	4	
82		Vero cells (other cell)	N/A	43.25	53.3	4	
83	35	DC2.4 cells (immune cell)	5%	237.8	34.8	2	√
84		DC2.4 cells (immune cell)	15%	275.8	29.0	2	√
85		DC2.4 cells (immune cell)	57%	220.1	37.3	2	√
86	36	BHK cells (other cell)	85%	92	2.7	1	
87		BHK cells (other cell)	90%	80	2.4	1	
88		BHK cells (other cell)	20%	88	1.3	1	
89		BHK cells (other cell)	25%	72	1.8	1	
90		BHK cells (other cell)	3%	66	1.9	1	
91		BHK cells (other cell)	5%	102	1.5	1	
92	37	DC2.4 cells (immune cell)	N/A	150	16	1	
93	38	HeLa cells (tumor cell)	10%	90-100	Positive	1	
94		HeLa cells (tumor cell)	5%	100	Positive	1	
95		HeLa cells (tumor cell)	6%	70-80	Positive	1	
96		HeLa cells (tumor cell)	2.5%	70-80	Positive	1	
97	39	BMDCs (immune cell)	70%	150	45	2	
98	40	DC (immune cell)	70.2%	606.45	12.2	2	
99	41	HeLa cells (tumor cell)	N/A	100	Negative	1	
100	42	B16 cells (tumor cell)	60%	135	25	1	
101	43	BMDCs (immune cell)	80%	155	0.03	1	
102	44	HEK293 cells (other cell)	N/A	217	47	1	
103		HEK293 cells (other cell)	N/A	182	44	1	
104		HEK293 cells (other cell)	N/A	147	34	1	
105		HEK293 cells (other cell)	N/A	166	38	1	

106		HEK293 cells (other cell)	N/A	202	28	1	
107		HEK293 cells (other cell)	N/A	234	27	1	
108		HEK293 cells (other cell)	N/A	148	48	1	
109		HEK293 cells (other cell)	N/A	146	39	1	
110		HEK293 cells (other cell)	N/A	160	35	1	
111	45	HEK293 cells (other cell)	N/A	284.4	Positive	2	
112		3T3 cells (other cell)	10%	170	8.4	2	
113		3T3 cells (other cell)	55%	140	28.2	2	
114		3T3 cells (other cell)	35%	133	22.1	2	
115	46	3T3 cells (other cell)	20%	201	4.2	2	
116		3T3 cells (other cell)	55%	198	5.4	2	
117		3T3 cells (other cell)	45%	186	17.1	2	
118		3T3 cells (other cell)	30%	137	17.6	2	
119		BMDCs (immune cell)	60%	144.7	-26.56	2	
120	47	BMDCs (immune cell)	55%	172.3	-31.78	2	
121		BMDCs (immune cell)	55%	154.7	-26.09	2	
122		HeLa cells (tumor cell)	N/A	66	-0.694	1	
123	48	HeLa cells (tumor cell)	N/A	70	-1.54	1	
124		HeLa cells (tumor cell)	N/A	68	-0.997	1	
125		DC (immune cell)	32%	184	34	1	√
126	49	DC (immune cell)	22%	178	19	1	√
127		DC (immune cell)	5%	188	20	1	
128		ARPE-19 cells (other cell)	65%	246.8	37.2	1	
129		ARPE-19 cells (other cell)	80%	210.1	36.5	1	
130		ARPE-19 cells (other cell)	65%	202.4	27.5	1	
131	50	ARPE-19 cells (other cell)	70%	349.2	18.5	1	
132		ARPE-19 cells (other cell)	60%	251.6	28.8	1	
133		ARPE-19 cells (other cell)	40%	233.7	25.4	1	
134		ARPE-19 cells (other cell)	85%	261.7	23.1	1	
135	51	Hela-GFP cells (tumor	50%	200	Negative	2	√

		cell)						
136	52	T22 cells (tumor cell)	30%	250	40	3		
137		T22 cells (tumor cell)	50%	250	40	3		
138	53	HeLa cells (tumor cell)	94%	50	46.8	2		
139	54	Hela-GFP cells (tumor cell)	N/A	300	-10	4		
140		Hela-GFP cells (tumor cell)	N/A	300	-8	4		
141		Hela-GFP cells (tumor cell)	N/A	300	-10	4		
142	55	DC2.4 cells (immune cell)	N/A	220	-0.52	4		
143	56	SVR-bag4 cells (other cell)	45%	15	30.7	4	√	
144	57	HT-1080 cells (tumor cell)	80%	150	-10.5	2		
145	58	HeLa cells (tumor cell)	72%	225	Negative	1		
146	59	Hela-GFP cells (tumor cell)	30%	60	-40	1		√
147	60	HepG2 cells	20%	300-500	N/A	1		
148		HepG2 cells	22.4%	300-500	N/A	1		
149		HepG2 cells	9.9%	300-500	N/A	1		
150		HepG2 cells (tumor cell)	40%	300-500	N/A	1		
151	61	KB cells (tumor cell)	60%	25.3	-15.2	3		
152	62	M4A4-GFP cells (tumor cell)	25%	204.1	-26.25	2		
153	63	COS-7 cells (other cell)	50%	224	7	2		
154	64	N/A	N/A	92	N/A	1		
155		N/A	N/A	78	N/A	1		
156		N/A	N/A	122	N/A	1		
157		N/A	N/A	75	N/A	1		
158	65	HeLa cells (tumor cell)	83.3%	N/A	N/A	4	√	
159	66	LNCaP cells (tumor cell)	N/A	110	35	1		
160	67	SKOV3 cells (tumor cell)	75%	190.1	1.5	2		
161	68	GBM319-GFP cells (tumor cell)	37%	207	22	2		
162		GBM319-GFP cells (tumor cell)	83%	204	-0.8	2		√
163	69	Hela-GFP cells (tumor cell)	50%	500	Neutral	2		

164	70	HeLa cells (tumor cell)	80%	193	30.7	2	√	
165		HeLa cells (tumor cell)	65%	460	22.8	2		
166	71	U-118 MG cells (tumor cell)	35%	150	–12	4		
167	72	HeLa-Luc cells (tumor cell)	80%	148	N/A	3		
168	73	MDA-MB-231 cells (tumor cell)	40%	25	16	2		√
169	74	MCF-7 cells (tumor cell)	84.5%	25	Negative	2		
170	75	Hep 3B cells (tumor cell)	N/A	100	30	1		
171	76	HeLa cells (tumor cell)	85%	N/A	N/A	1		
172	77	HeLa cells (tumor cell)	70%	111	N/A	1		
173		HeLa cells (tumor cell)	70%	81	N/A	1		
174		HeLa cells (tumor cell)	5%	62	N/A	1		
175		HeLa cells (tumor cell)	60%	64	N/A	1		
176		HeLa cells (tumor cell)	50%	58	N/A	1		
177		HeLa cells (tumor cell)	80%	124	N/A	1		
178		HeLa cells (tumor cell)	90%	165	N/A	1		
179	78	PANC-1-GFP cells (tumor cell)	61.7%	130	Positive	4		
180	79	U-87 MG-GFP cells (tumor cell)	85%	N/A	18	4	√	√
181		U-87 MG-GFP cells (tumor cell)	70%	N/A	Positive	4	√	√
182		U-87 MG-GFP cells (tumor cell)	65%	N/A	Positive	4		√
183	80	Jurkat T cells (immune cell)	85%	156	50	3		
184	81	HeLa-Luc cells (tumor cell)	0%	98	-1.91	1		
185		HeLa-Luc cells (tumor cell)	15%	75	-0.88	1		
186		HeLa-Luc cells (tumor cell)	60%	124	1.2	1		
187		HeLa-Luc cells (tumor cell)	5%	106	-1.01	1		
188		HeLa-Luc cells (tumor cell)	5%	186	-1.09	1		
189		HeLa-Luc cells (tumor cell)	50%	101	-1.56	1		√
190		HeLa-Luc cells (tumor	25%	72	-0.98	1		√

		cell)						
191		HeLa-Luc cells (tumor cell)	90%	132	-1.07	1		√
192		HeLa-Luc cells (tumor cell)	5%	112	-0.96	1		√
193		HeLa-Luc cells (tumor cell)	5%	173	-1.14	1		√
194	82	bEnd.3 endothelial cells (other cell)	80%	67	-10.7	2		
195		bEnd.3 endothelial cells (other cell)	85%	74	10.4	2		
196		bEnd.3 endothelial cells (other cell)	90%	60	-13.2	2		
197	83	Hela-GFP cells (tumor cell)	31%	200	-6.94	4		
198		Hela-GFP cells (tumor cell)	85%	200	2.54	4		
199	84	SK-HEP-1-Luc cells (tumor cell)	40%	109	2.07	1		
200		SK-HEP-1-Luc cells (tumor cell)	65%	101	13.37	1		
201		SK-HEP-1-Luc cells (tumor cell)	90%	113	9.91	1		
202	85	human mesenchymal stem cells (other cell)	80%	60-80	Positive	2		√
203	86	SW480 cells (tumor cell)	70%	350	15	4	√	
204	87	143B-fluc cells (tumor cell)	90%	200	-6	4	√	
205	88	MCF7-GFP cells (tumor cell)	88%	23	N/A	4		√
206		MCF7-GFP cells (tumor cell)	76%	30	8	4	√	
207	89	N/A	N/A	190	52.1	1		
208	90	HeLa-Luc cells (tumor cell)	65%	100	18	1		
209		HeLa-Luc cells (tumor cell)	60%	100	22	1		
210		HeLa-Luc cells (tumor cell)	35%	100	15	1		
211		HeLa-Luc cells (tumor cell)	60%	100	Positive	1		
212		HeLa-Luc cells (tumor	90%	150	Positive	1		

		cell)						
213	91	911-Luc cells (other cell)	10%	N/A	Positive	2		
214		911-Luc cells (other cell)	65%	N/A	Positive	2		
215	92	MDA-MB-231-Luc cells (tumor cell)	15%	250	30	2		√
216	93	PC3 cells (tumor cell)	95%	400	20	2		
217		PC3 cells (tumor cell)	90%	N/A	N/A	2		
218		PC3 cells (tumor cell)	80%	N/A	N/A	2		
219	94	KB cells (tumor cell)	25%	229	18	1		
220		KB cells (tumor cell)	25%	230	16.5	1		
221	95	DU145-GFP cells (tumor cell)	50%	196	-5	4	√	√
222	96	HeLa-Luc cells (tumor cell)	75%	160	3.5	1		
223		HeLa-Luc cells (tumor cell)	75%	130	7.5	1		
224		HeLa-Luc cells (tumor cell)	75%	110	1.8	1		
225		HeLa-Luc cells (tumor cell)	93%	120	7	1		
226	97	M213 cells (tumor cell)	N/A	160	30	2		
227		N/A	N/A	125	21	2		
228		N/A	N/A	30	27.5	2		
229	98	Hela-GFP cells (tumor cell)	90%	60	N/A	1		
230	99	MDA-MB-435s-GFP cells (tumor cell)	58%	18.2	12	4		
231		MDA-MB-435s-GFP cells (tumor cell)	20%	50.7	40.7	2		
232	100	Hela-GFP cells (tumor cell)	90%	50	23	2		
233		N/A	N/A	100	13	2		
234		B16F10-luc cells (tumor cell)	0%	100	15	2		
235		B16F10-luc cells (tumor cell)	80%	50	12	2		
236		B16F10-luc cells (tumor cell)	90%	N/A	Positive	2		
237	101	FL83B cells (other cell)	90%	140	Positive	2		√
238	102	MDA-MB-231 cells (tumor cell)	N/A	79.6	24	2		

239		MDA-MB-231 cells (tumor cell)	41.2%	91	-14.7	2		√
240		MDA-MB-231 cells (tumor cell)	20%	93	-14	2		
241		HeLa cells (tumor cell)	50%	120	25	2		√
242	103	HeLa cells (tumor cell)	58%	120	25	2		√
243		HeLa cells (tumor cell)	65%	120	20	2		√
244		NIH 3T3-GFP cells (other cell)	40%	138.6	18	2		
245		NIH 3T3-GFP cells (other cell)	0%	81.8	11.3	2		
246		NIH 3T3-GFP cells (other cell)	35%	77.3	13.6	2		
247	104	NIH 3T3-GFP cells (other cell)	70%	74.5	13.8	2		
248		NIH 3T3-GFP cells (other cell)	60%	190.1	20.7	2		
249		NIH 3T3-GFP cells (other cell)	80%	78.2	14.9	2		
250		C4-2B prostate cells (tumor cell)	50%	200	Negative	4		
251	105	C4-2B prostate cells (tumor cell)	20%	150	-20	4		
252		C4-2B prostate cells (tumor cell)	50%	200	Negative	4		
253	106	CHO-GFP cells (other cell)	60%	150-180	Positive	2		
254	107	SKOV3 ovarian cancer cells (tumor cell)	80%	N/A	Positive	2	√	√
255	108	MCF-7 cells (tumor cell)	42.4%	195	Positive	2	√	
256	109	PC3 cells (tumor cell)	90%	120	5.96	1		
257	110	P53-null Hep3B liver cancer cells (tumor cell)	90%	125	Positive	1		√
258	111	B16F10 cell (tumor cell)	55%	90	Positive	2		√

(a) The category of these nanoparticles could be divided into 4 types, 1: liposomes, 2: polymeric NPs, 3: biomimetic NPs, 4: inorganic NPs.

Supplementary Table 2. The database of nanoparticles (n=158) for lymph node delivery.

NO.	Ref. number	Size (nm)	Surface charge (mV)	Category (b)
1	112	25	-19.9	1
2	113	18.7	-12.6	4
3		82.2	-20.4	4
4		125.3	-23	4
5		167.5	-20.2	4
6	114	14	N/A	4
7		23	N/A	4
8	115	49.9	-17.8	2
9		95.6	-34.4	2
10		243.3	-61.5	2
11		788.2	-78	2
12	116	258	27.6	2
13	117	5.1	-30	4
14	118	80.27	-9.34	4
15		88.52	0.02	1
16	119	246.8	-33.6	2
17		192	-39.3	2
18		191.3	-27.5	2
19	120	190.7	32.1	2
20		122.6	-45.7	2
21	121	77	4	4
22	122	30	-0.5	2
23		73	-3.1	2
24	123	93.9	-34.52	2
25		48.1	-46.87	2
26	124	5	N/A	2
27		30	N/A	2
28		500	N/A	2
29	125	170	10	2
30	126	157.7	18.1	1
31	127	20-30	N/A	1
32	128	18-20	-45	4
33	129	57	-3	2
34	130	100	45	2

35		100	35	2
36	131	44.2	4-5	2
37	132	330	-13	3
38		330	N/A	3
39	133	64.9	N/A	2
40		58.5	N/A	2
41	134	20	N/A	2
42		20	N/A	2
43		200	N/A	2
44		200	N/A	2
45	135	196.5	-25.2	2
46		141.4	-22.5	2
47		149.4	-18.1	2
48		149.7	-21.1	2
49		157.7	-26	2
50		268.5	-29.4	2
51	136	278	-25	2
52		154	-10	2
53	137	25	Negative	1
54		100	Negative	1
55	138	83.1	-10.14	2
56		87.1	-12.34	2
57		87.8	-20.51	2
58		91.3	-17.33	2
59	139	22.4	-38.3	4
60		30	-38.5	4
61		43.6	-44.1	4
62		58.1	-36.4	4
63		76.5	-35.2	4
64	140	62.85	-16.2	2
65		103.5	-8.86	2
66	141	203.2	-1.8	1
67		101.1	-2.2	1
68		29.8	0.2	1
69		29.6	2.4	1
70		24.8	14.5	1
71		33.5	-11.9	1

72	142	17.1	-13.1	2
73		40.9	-10.9	2
74		106.9	-6.8	2
75	143	10.2	26.6	4
76		21.5	28.2	4
77		32.7	23.7	4
78	144	29.1	-12	3
79		30.4	-7	3
80		32	-18	3
81		31.4	-16	3
82	145	340	N/A	2
83	146	30	Positive	2
84	147	52	Negative	2
85		47	Negative	2
86	148	155.58	Negative	2
87		210.45	Negative	2
88		213.9	Negative	2
89	149	20.2	-24.5	4
90	150	15.7	N/A	4
91	151	2-4	N/A	4
92		2	N/A	4
93		4	N/A	4
94	152	38.05	-9	4
95	153	110	-20.56	2
96	154	22.60	31.4	2
97	155	63.3	Positive	4
98	156	200	Negative	1
99	157	40	-17	4
100	158	162.6	5.5	2
101	159	87.95	-9.27	4
102	160	10-20	Positive	1
103	161	24.6	Negative	4
104	162	82	38	4
105	163	24.87	-48.9	2
106	164	63.0	-1.8	2
107	165	54.2	-20.7	2
108	166	25	Negative	2

109	167	30	Negative	2
110		60	Negative	2
111		100	Negative	2
112		200	Negative	2
113	168	4.8	-55.4	4
114	169	106	Negative	2
115	170	10.8	Negative	2
116	171	7	Negative	2
117	172	220	10	2
118	173	80.12	17.42	2
119	174	100	-25	3
120	175	< 45	Negative	2
121	176	41.9	-12	3
122	177	163	N/A	2
123	178	210	-29.5	1
124		218.3	-11.3	1
125		219.5	-13.2	1
126	179	279.8	N/A	2
127		296.4	N/A	2
128		313	N/A	2
129	180	60	-4	2
130		82	-4	2
131		100	-3	2
132		83	-16	3
133		103	-16	3
134		122	-15	3
135	181	280	20	4
136	182	20-50	N/A	2
137		20-50	N/A	2
138	183	220	-1.5	1
139		218	-1.62	1
140	184	156	-16.4	2
141	185	30	15	2
142	186	357	-20.3	2
143	187	148	-27	3
144	188	54	1.12	1

145		43	0.34	1
146	189	310	-45.6	4
147	190	64	12	4
148		73	11	4
149	191	30-40	N/A	2
150	192	34.3	-20.8	3
151	193	90	N/A	3
152	194	165.7	50	2
153		182	50	2
154	195	252	1.5	3
155	196	50.4	1.6	2
156		71.8	1.1	2
157		69.7	1.1	2
158	197	10.5	N/A	3

(b) The category of these nanoparticles could be divided into 4 types, including 1: liposomes, 2: polymeric NPs, 3: biomimetic NPs, 4: inorganic NPs.

Supplementary Table 3. Prediction of mRNA transfection efficiency when prepared from OEI or OEI-PBA at different weight ratios to EGFP mRNA. The predicted efficiencies were divided into three classes (<40% (class 0), 40%-60% (class 1) and >60% (class 2)). The applied cell line was DC2.4 cells. The category of all nanoparticles was polymeric nanoparticle. All nanoparticles were without stimuli-responsive property.

Polymer	Polymer to mRNA ratio (W/W)	Diameter (nm)	ζ potential (mV)	Modify	Predicted probability			Predicted class
					<40% (class 0)	40%-60% (class1)	>60% (class 2)	
OEI	5	178.5	26.4		0.273	0.167	0.560	**2**
	10	160.6	29.2		0.510	0.193	0.297	**0**
	20	97.9	31.1		0.684	0.062	0.254	**0**
	40	56.1	24.8		0.215	0.209	0.576	**2**
	60	37.1	22.8		0.150	0.373	0.476	**2**
	80	36.5	29.5		0.429	0.242	0.330	**0**
	100	22.1	20.2		0.285	0.562	0.154	**1**
OEI-PBA 1.4	5	71.5	26.9	√	0.844	0.023	0.133	**0**
	10	30.8	27.5	√	0.746	0.060	0.194	**0**
	20	42.6	26.9	√	0.915	0.041	0.044	**0**
	40	75.1	30.6	√	0.934	0.025	0.041	**0**
	60	57.7	28.9	√	0.714	0.136	0.150	**0**
	80	44.8	32.1	√	0.888	0.083	0.029	**0**
	100	33.1	33.2	√	0.726	0.130	0.144	**0**
OEI-PBA 2.9	5	87.6	25.6	√	0.364	0.428	0.207	**1**
	10	76.4	31.9	√	0.934	0.025	0.041	**0**
	20	92.1	32.5	√	0.698	0.223	0.079	**0**
	40	39.7	35.4	√	0.962	0.024	0.014	**0**
	60	109.3	38.8	√	0.640	0.176	0.184	**0**
	80	75.7	31.8	√	0.933	0.026	0.041	**0**
	100	76.9	34.1	√	0.961	0.015	0.024	**0**
OEI-PBA 6.0	5	74	25.1	√	0.525	0.077	0.398	**0**
	10	67.8	20.9	√	0.474	0.144	0.381	**0**
	20	36.9	27.7	√	0.870	0.070	0.060	**0**
	40	29.3	22.2	√	0.241	0.089	0.670	**2**
	60	33.7	18.5	√	0.324	0.316	0.360	**2**
	80	29.5	21.7	√	0.218	0.132	0.651	**2**
	100	23.5	22.3	√	0.240	0.144	0.616	**2**

Supplementary Table 4. Prediction of mRNA transfection efficiency when prepared from PAMAM (G2) or PAMAM-PBA at different weight ratios to EGFP-mRNA in DC2.4 cells. The predicted efficiencies were divided into three classes (<40% (class 0), 40%-60% (class 1) and >60% (class 2)). The category was polymeric nanoparticle. All nanoparticles were without stimuli-responsive property.

Polymer	Polymer to mRNA ratio (W/W)	Diameter (nm)	ζ potential (mV)	Modify	Predicted probability			Predicted class
					<40% (class 0)	40%-60% (class1)	>60% (class 2)	
PAMAM	5	378.4	37.9		0.484	0.367	0.148	**0**
	10	338.5	38.4		0.522	0.330	0.148	**0**
	20	385.6	34.3		0.692	0.178	0.129	**0**
	40	292.4	35.2		0.860	0.053	0.086	**0**
	60	278.4	32.7		0.958	0.026	0.015	**0**
	80	282.4	33.9		0.977	0.014	0.009	**0**
	100	288.9	36.1		0.857	0.058	0.085	**0**
PAMAM-PBA0.9	5	329.2	35.9	√	0.813	0.123	0.064	**0**
	10	343.2	30.3	√	0.722	0.126	0.152	**0**
	20	320.6	33.5	√	0.763	0.127	0.110	**0**
	40	302.6	29.4	√	0.875	0.038	0.087	**0**
	60	221.3	32.9	√	0.450	0.500	0.050	**1**
	80	224.7	30.6	√	0.637	0.301	0.062	**0**
	100	228.9	37	√	0.736	0.245	0.019	**0**
PAMAM-PBA3.6	5	317.1	32.4	√	0.819	0.087	0.094	**0**
	10	271.4	21.8	√	0.537	0.312	0.152	**0**
	20	346.9	29.5	√	0.718	0.128	0.154	**0**
	40	280.8	29.1	√	0.977	0.008	0.015	**0**
	60	251.3	29.8	√	0.818	0.095	0.088	**0**
	80	169.9	29.3	√	0.693	0.214	0.094	**0**
	100	194	41.7	√	0.593	0.071	0.336	**0**
PAMAM-PBA6.6	5	221.6	18	√	0.704	0.218	0.078	**0**
	10	249.3	10.3	√	0.593	0.191	0.216	**0**
	20	213.8	16.6	√	0.618	0.288	0.094	**0**
	40	168.9	27.1	√	0.697	0.190	0.114	**0**
	60	219.8	35.8	√	0.632	0.345	0.023	**0**
	80	203.8	37	√	0.873	0.088	0.039	**0**
	100	165.3	42.2	√	0.518	0.139	0.343	**0**

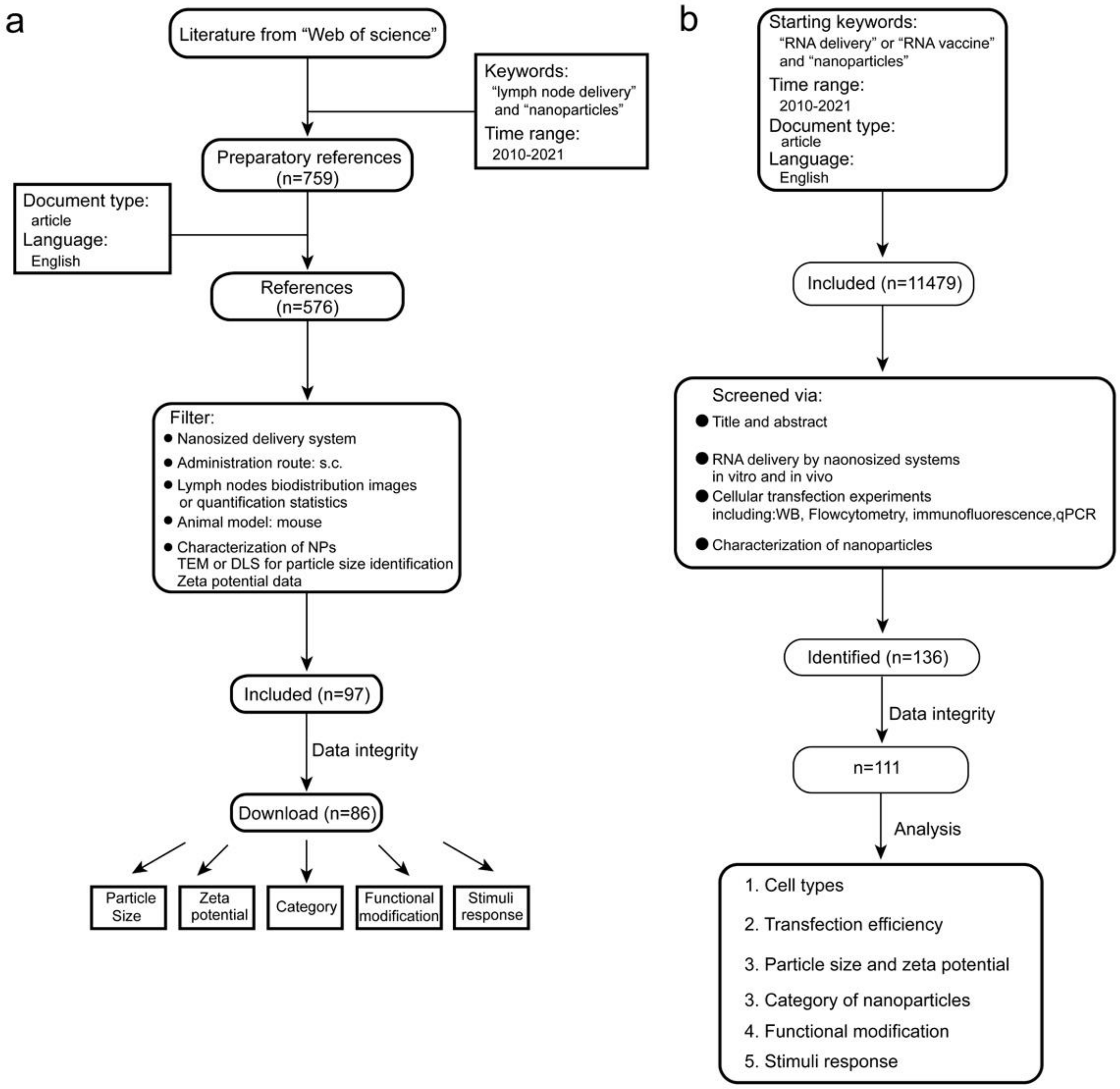

Supplementary Figure 1. The procedure and inclusion criteria for searching literatures. a, sorted references that related to delivery of drugs to lymph node. b, sorted references that related to cytosolic delivery of RNA.

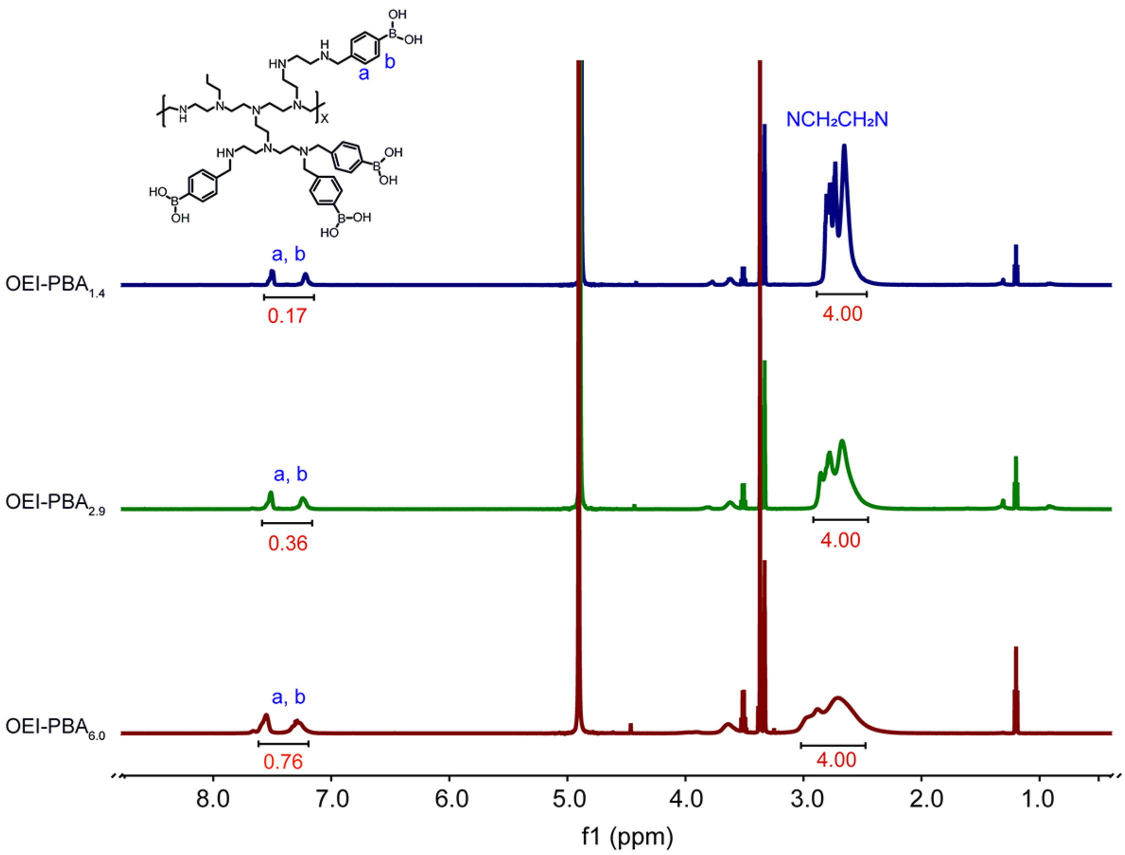

Supplementary Figure 2. ^{1}H-NMR spectrum of PBA-grafted OEI. The number of PBA conjugated on each OEI was confirmed via peak area.

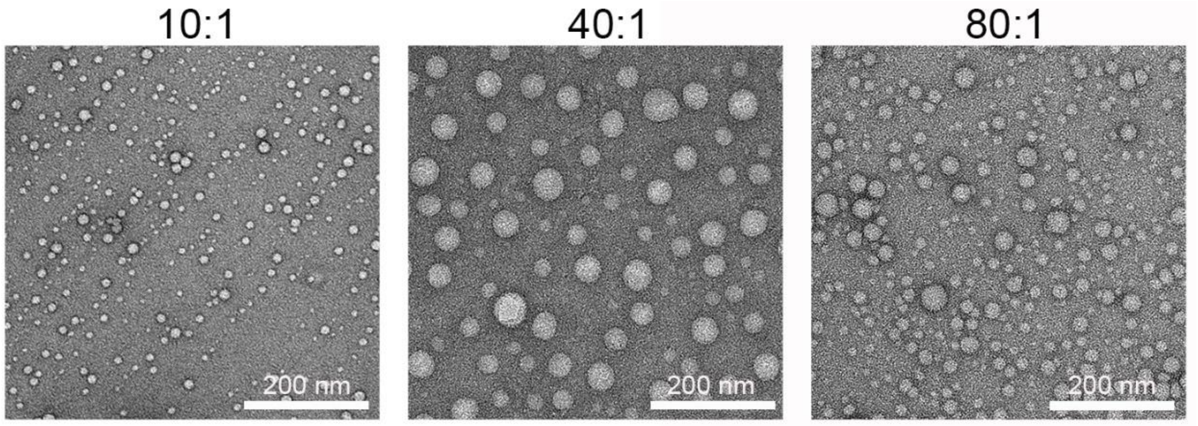

Supplementary Figure 3. Representative TEM images of ORG nanoparticles (OEI-PBA$_{6.0}$). The weight ratio of OEI-PBA$_{6.0}$ to mRNA in each formulation is 10:1, 40:1, 80:1, respectively. Scale bars, 200 nm.

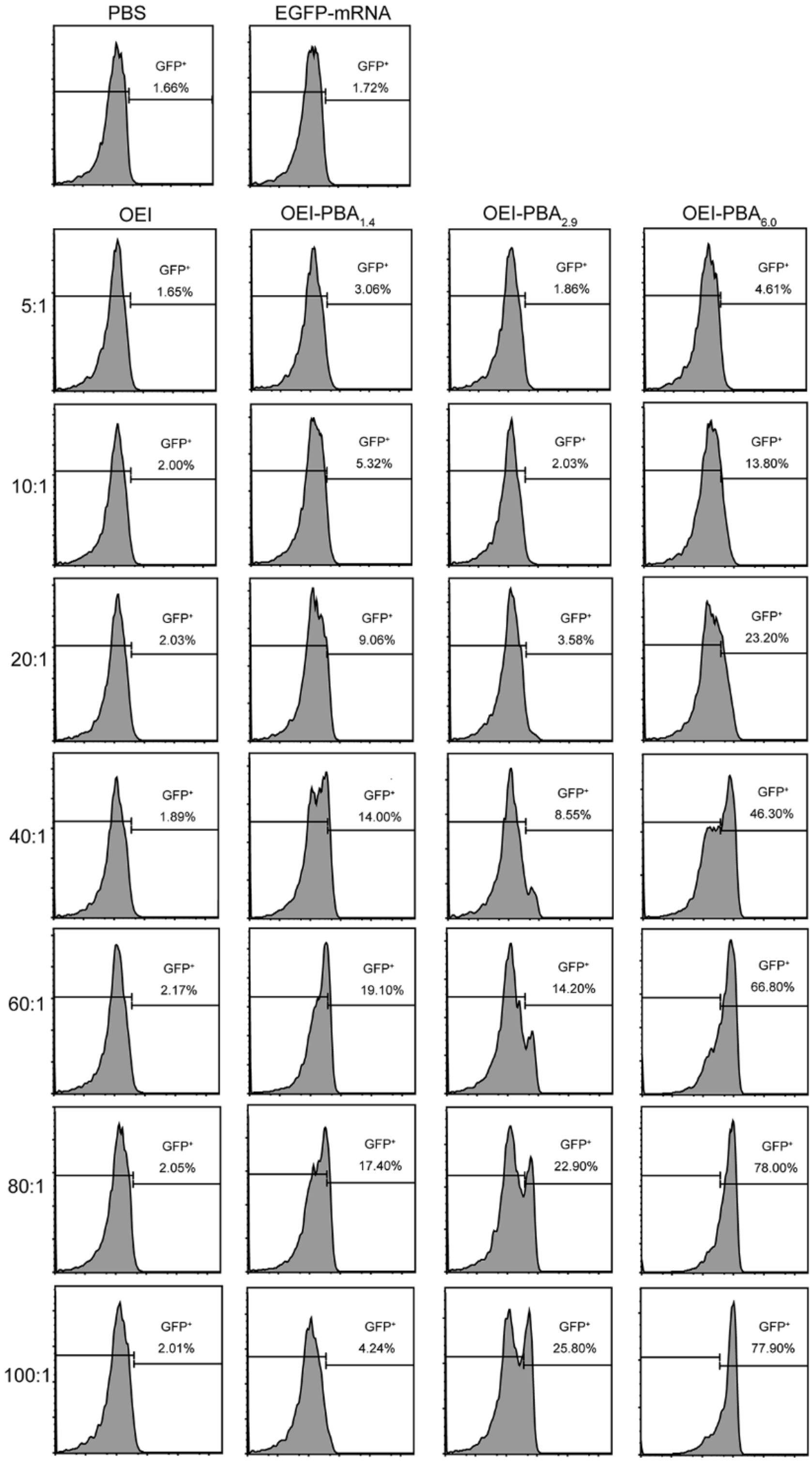

Supplementary Figure 4. The histogram analysis for the transfection efficiency in DC2.4 cells treated with PBS, free EGFP-mRNA, OEI/EGFP-mRNA, OEI-PBA$_{1.4}$/EGFP- mRNA, OEI-PBA$_{2.9}$/EGFP- mRNA and OEI-PBA$_{6.0}$/EGFP- mRNA at various ratios (5:1, 10:1, 20:1, 40:1, 60:1, 80:1, 100:1). Representative histogram images in each group were shown.

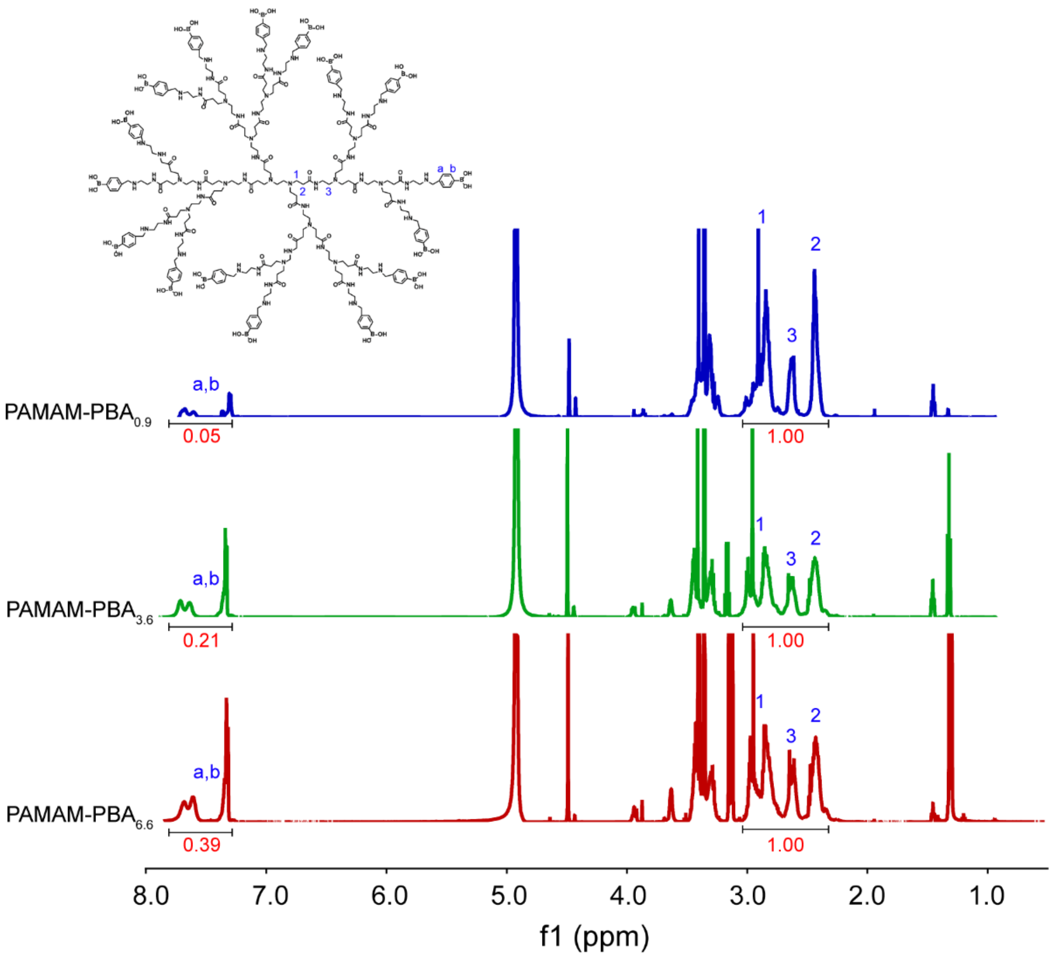

Supplementary Figure 5. ^{1}H-NMR spectrum of PBA-grafted PAMAM (G2). The number of PBA conjugated on each polymer was confirmed via peak area.

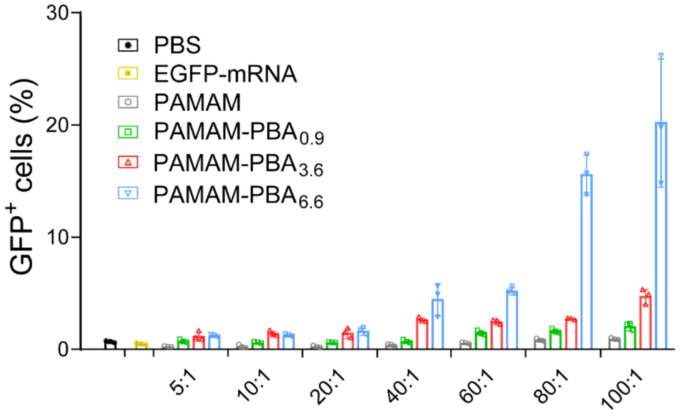

Supplementary Figure 6. Frequency of GFP^+ DC2.4 cells after treated by PBS, EGFP-mRNA, PAMAM/EGFP-mRNA, PAMAM-PBA/EGFP-mRNA nanocomplexes at different weight ratios (5:1, 10:1, 20:1, 40:1, 60:1, 80:1 and 100:1) (n = 3).

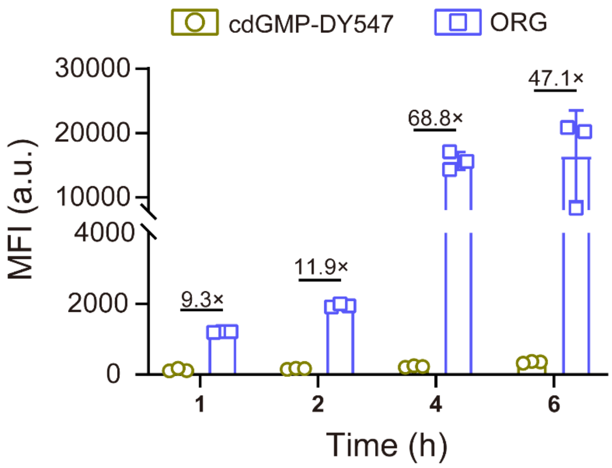

Supplementary Figure 7. Cellular uptake of cdGMP-DY547 in DC 2.4 cells. Flow cytometry determined the mean fluorescence intensity of DY547 in DC2.4 cells after incubation with cdGMP-DY547 or ORG for indicated time points.

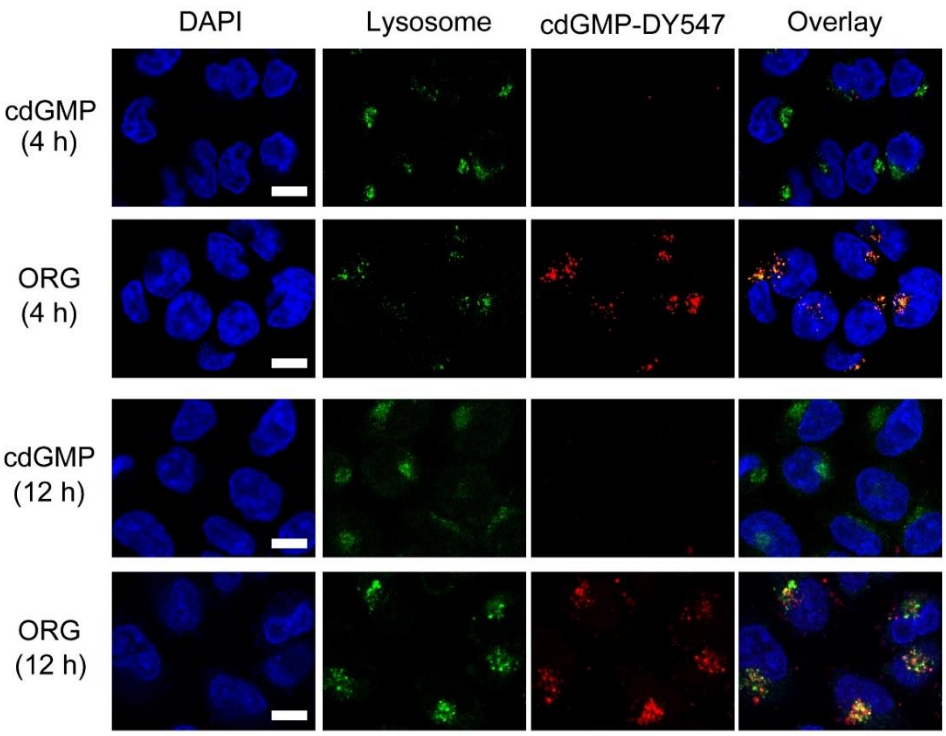

Supplementary Figure 8. Confocal images showing cellular uptake of cdGMP-DY547 (red) in DC2.4 cells, treating with cdGMP-DY547 or ORG for 4 h and 12 h. The nuclei and lysotracker were stained with DAPI (blue) and lysotracker (green) before observation, respectively. Scale bars, 10 μm.

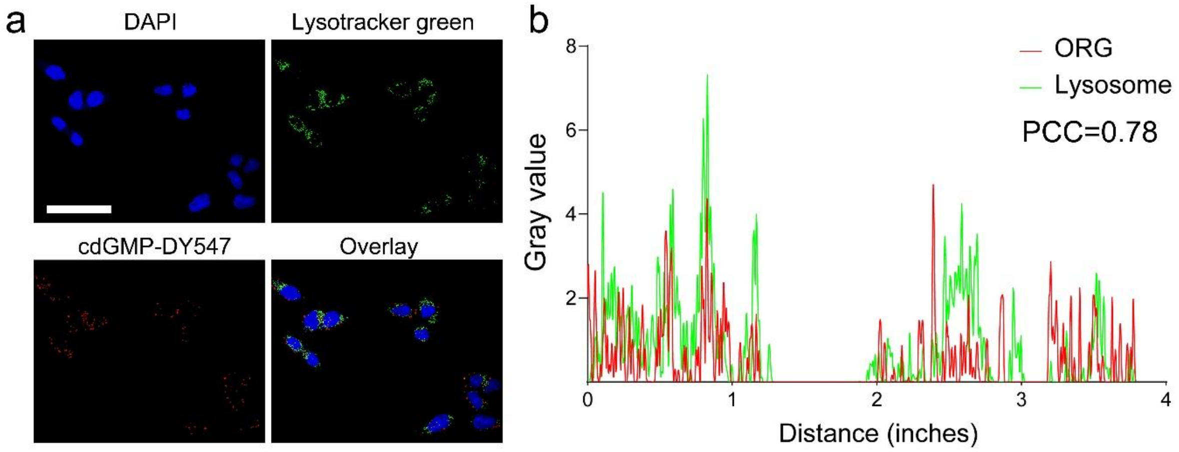

Supplementary Figure 9. (a) Confocal images showing cellular uptake of ORG in DC2.4 cells at 12 h (Scale bar = 50 μm). (b) Quantitative analysis of the gray value from lysotracker green and cdGMP-DY547 signals versus distance of the CLSM images. The pearson correlation coefficient (PCC) between the two signals was 0.78.

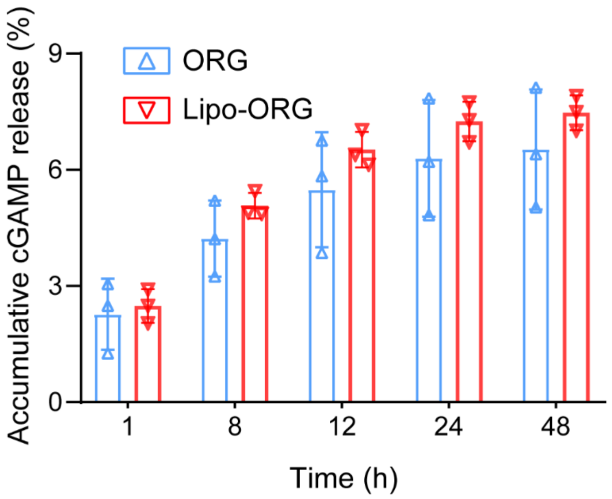

Supplementary Figure 10. Accumulative release of cGAMP from ORG or Lipo-ORG within 48 h. The concentration of released cGAMP was measured via an ELISA kit.

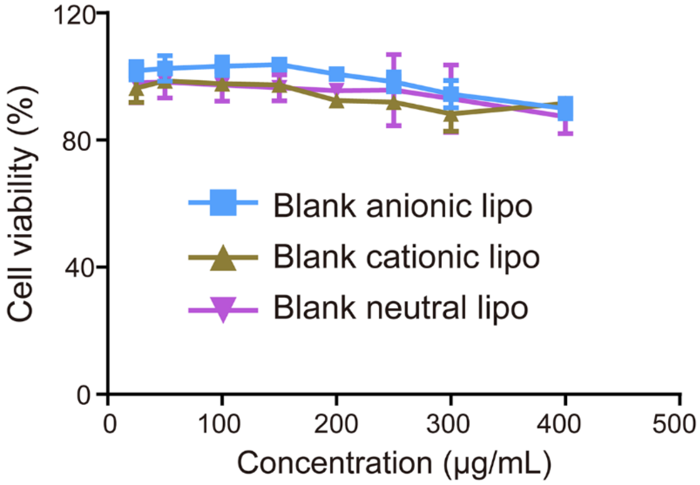

Supplementary Figure 11. Cell viability of DC 2.4 cells treated with different blank liposomes. DC 2.4 cells were incubated with anionic liposomes, cationic liposomes and neutral liposomes at a series concentration for 24 h, and the cell viability was measured by CCK8 assay.

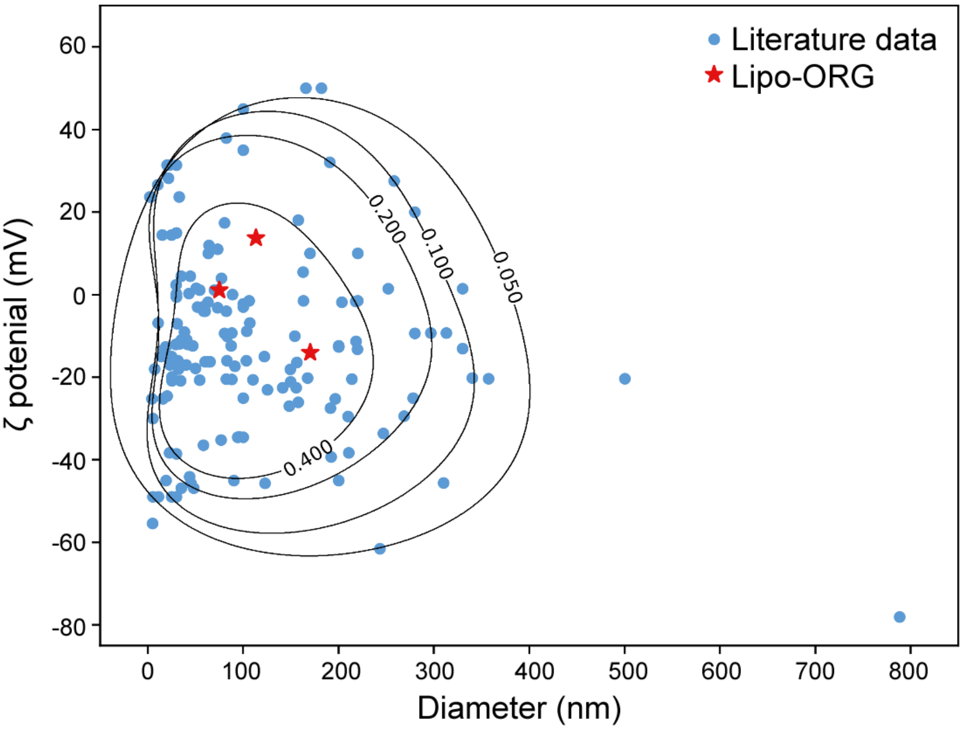

Supplementary Figure 12. Lymph node delivery data (n=158) were analyzed by one-class SVM. The contour plot indicated the upper bounds on the fraction of training errors, which is divided into four scenarios, 5%, 10%, 20% and 40%. The value of the upper bound can also be considered as the fraction of outliers in all the samples.

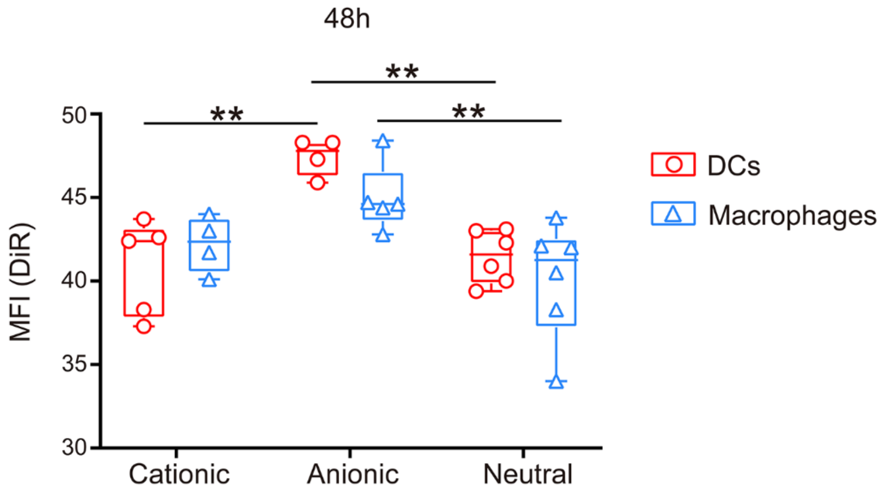

Supplementary Figure 13. Flow cytometry analysis of cellular uptake of LNPs (DiR) in DCs ($CD45^+CD11c^+MHCII^+$) and macrophages ($CD45^+CD11b^+F4/80^+$) from ex LNs at 48 h.

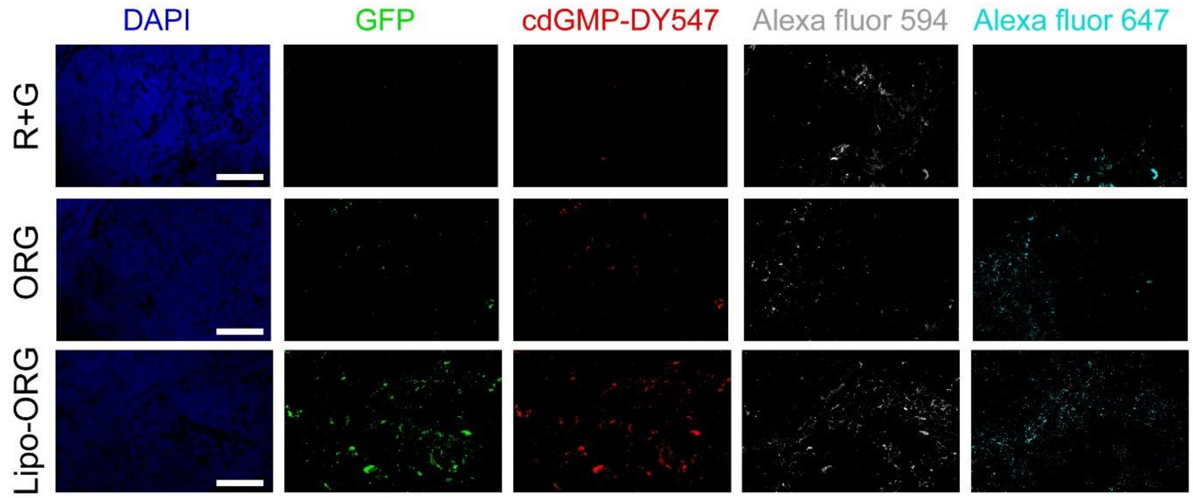

Supplementary Figure 14. Fluorescent images of transfected GFP proteins (green) and cdGMP-DY547 (red) in LN sections of mice after various treatments for 24 h. DCs were stained with CD11c-Alexa Fluor 594 (silver). Macrophages were stained by F4/80-Alexa Fluor 647 (wathet). Scale bars = 50 μm.

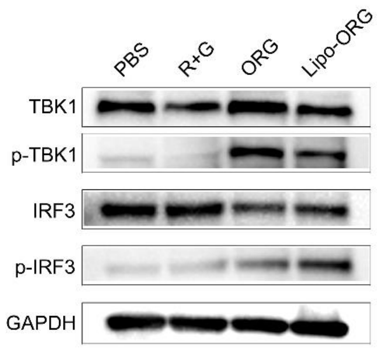

Supplementary Figure 15. Examination of key proteins including TBK1, phosphorylated TBK1 (p-TBK1), IRF3 and p-IRF3 in BMDCs with different treatments (0.5 μg/mL of mRNA, 0.5 μg/mL of cGAMP). GAPDH was set as control.

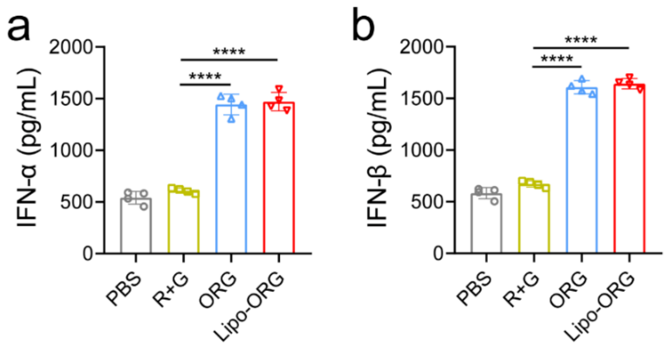

Supplementary Figure 16. Secretion levels of type I IFN in BMDCs after treated by different suspensions for 24 h. (a) IFN-α, (b) IFN-β in cell culture medium.

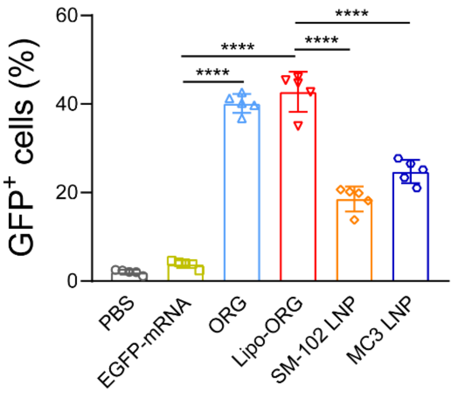

Supplementary Figure 17. Frequency of GFP^+ BMDCs after treated by PBS, EGFP-mRNA, ORG, Lipo-ORG, SM-102 LNP and DLin-MC3-DMA (MC3) LNP for 24 h, respectively. SM-102 and MC3 LNPs were prepared with commercial lipids as positive controls. The dosage of EGFP-mRNA was 0.5 μg/mL.

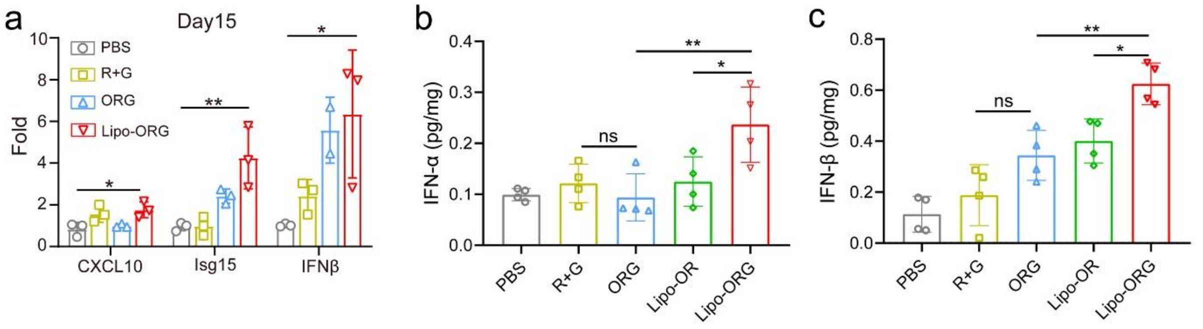

Supplementary Figure 18. (a) qPCR assay of STING-stimulated genes (CXCL10, ISG 15, and IFN-β) expression in LNs on day 15 after vaccination. (b) Normalized secretion levels of IFN-α, and (c) IFN-β in tumors (n = 4).

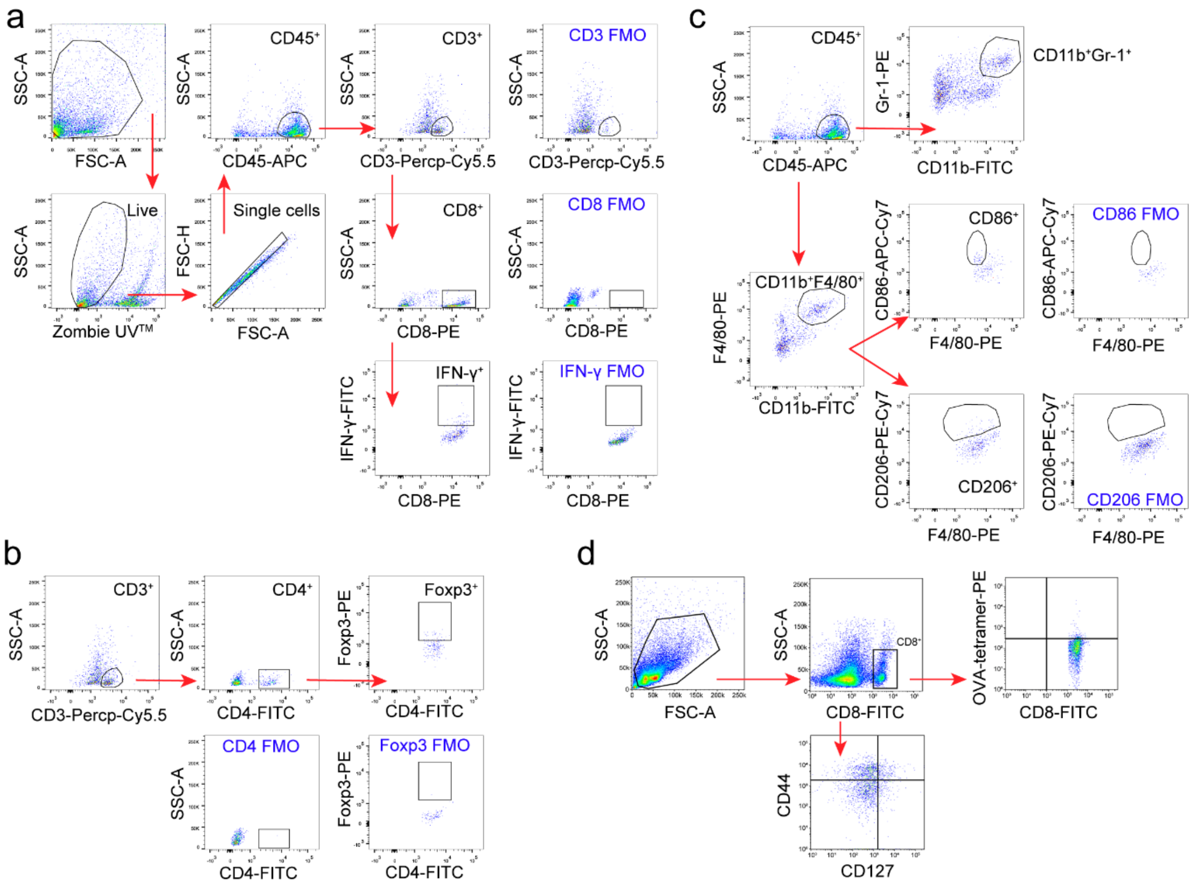

Supplementary Figure 19. Flow cytometry gating strategy for evaluating immune responses in vivo by Flowjo software. (a) Gating strategy for $CD8^+$ and IFN-γ^+CD8^+ T cells. (b) Gating strategy for $CD4^+$ T cells and Tregs. (c) Gating strategy for MDSCs, M1 and M2 macrophages. (d) Gating strategy of OVA-tetramer positive $CD8^+$ T cells and $CD8^+T_{CM}$ ($CD44^+CD127^+$).

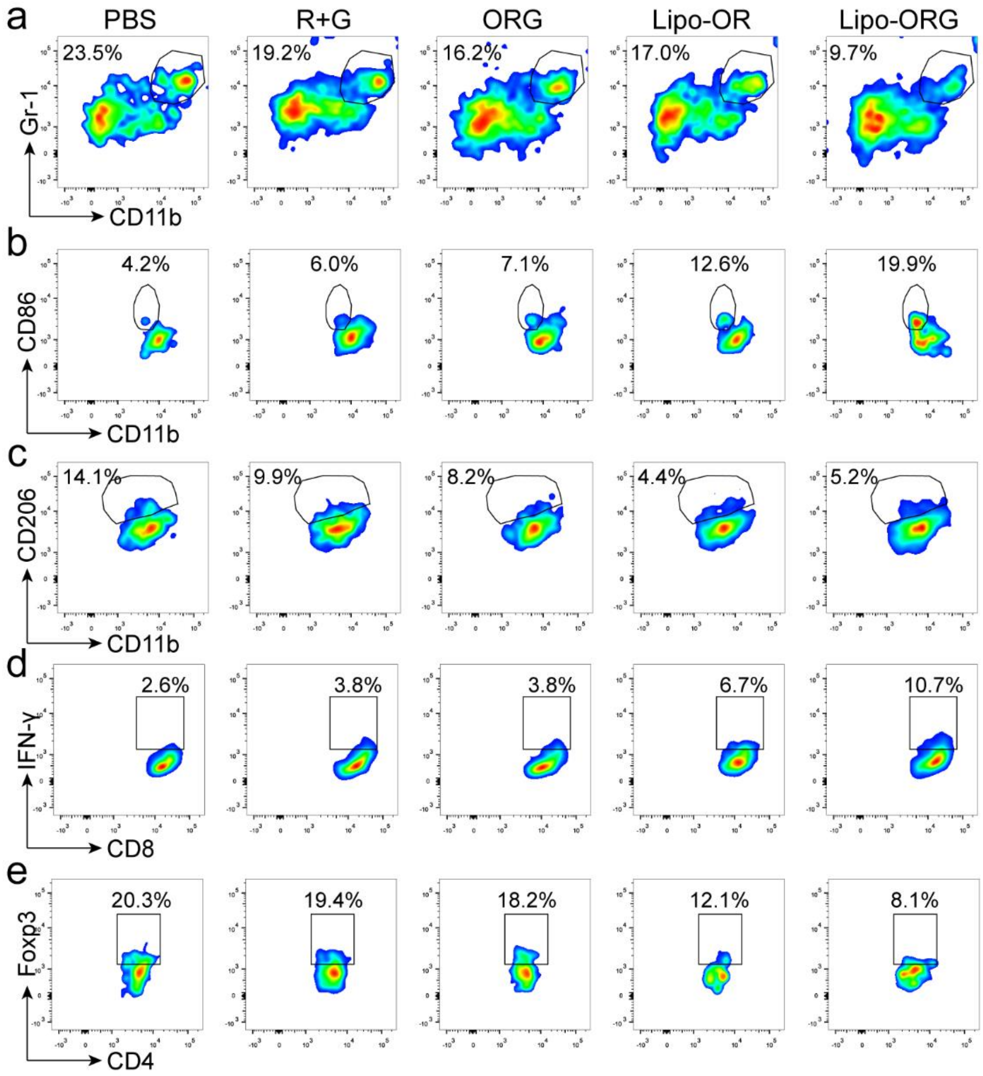

Supplementary Figure 20. Representative flow cytometry images for (a) MDSCs, (b) M1 macrophages, (c) M2 macrophages, (d) IFN-γ^+CD8$^+$ T cells and (e) Tregs in tumors.

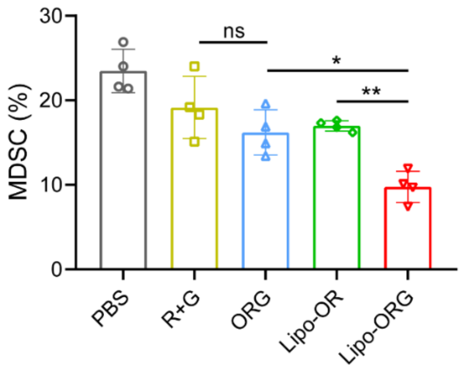

Supplementary Figure 21. Frequency of MDSCs (gated on CD45$^+$) in tumors of mice with various treatments (n = 4).

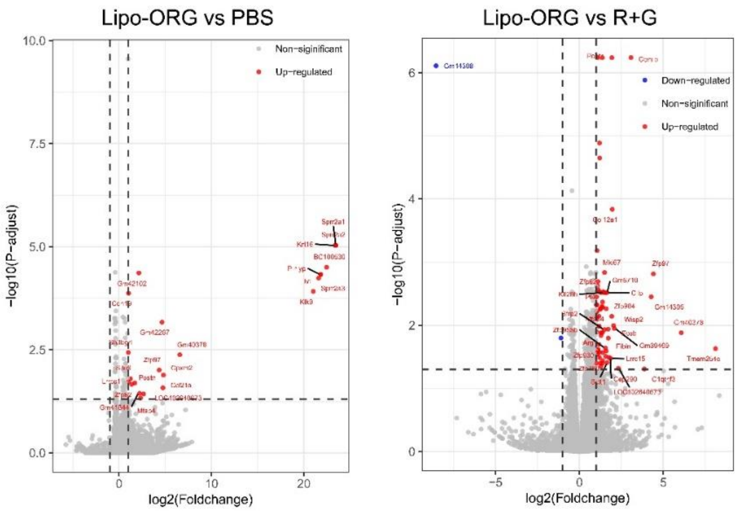

Supplementary Figure 22. Volcano maps for top 30 differential genes between versus groups. The names of differential genes were named in the maps. Lipo-ORG vs PBS: the expression of genes in PBS group was base line. Lipo-ORG vs R+G: the expression of genes in R+G group was base line.

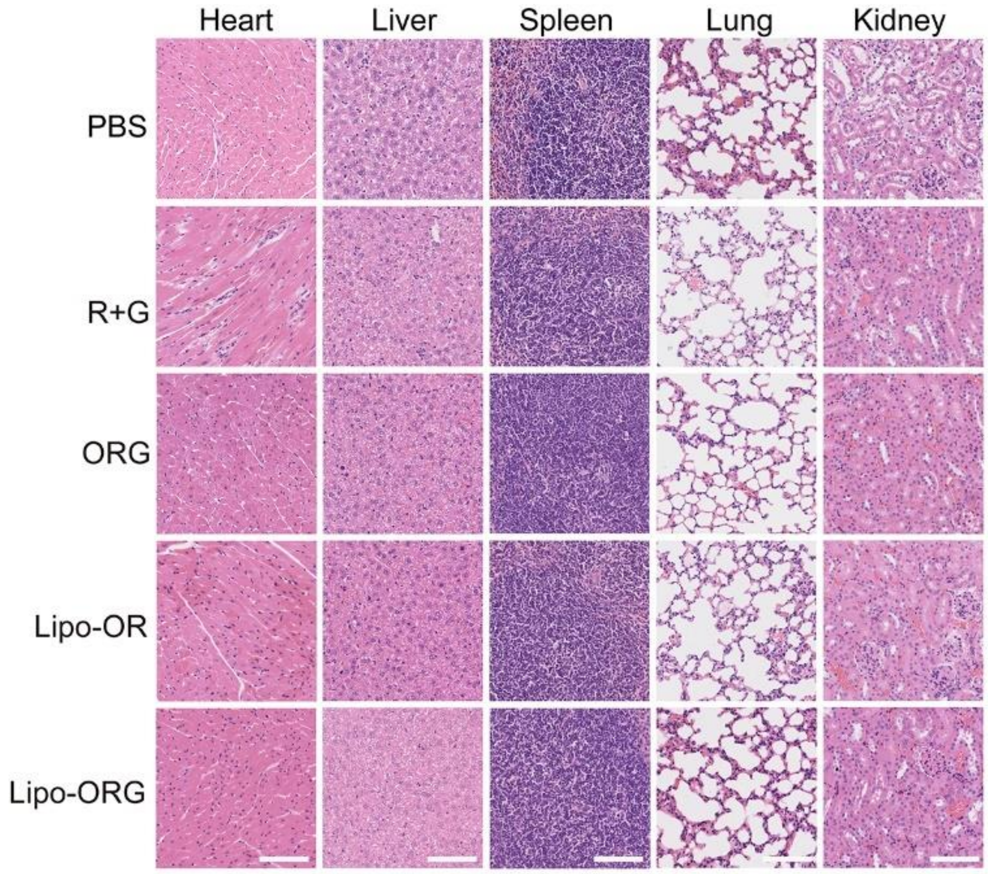

Supplementary Figure 23. H&E staining of major organs from mice following with indicated treatments. Scale bars, 100 μm.

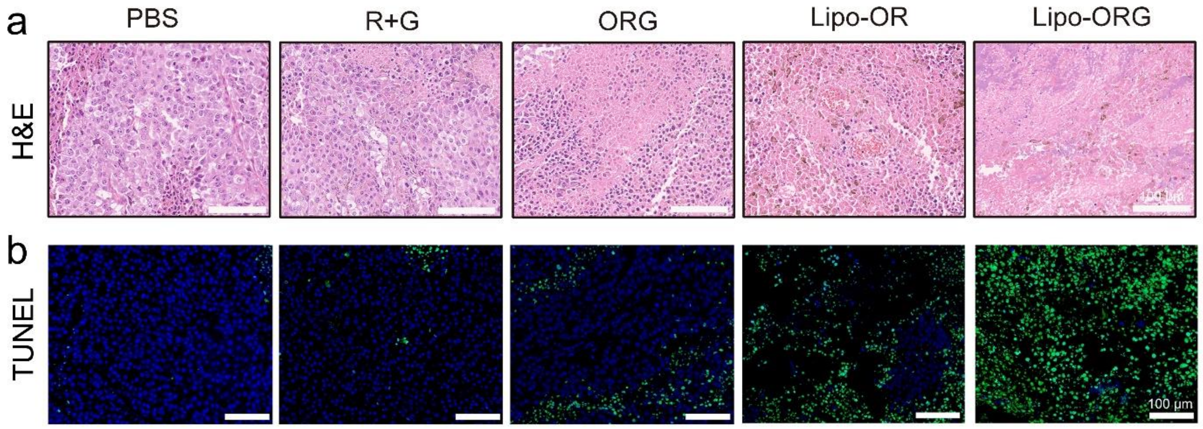

Supplementary Figure 24. (a) H&E, and (b) TUNEL staining of tumor sections at the end of antitumor study. Scale bars, 100 μm.

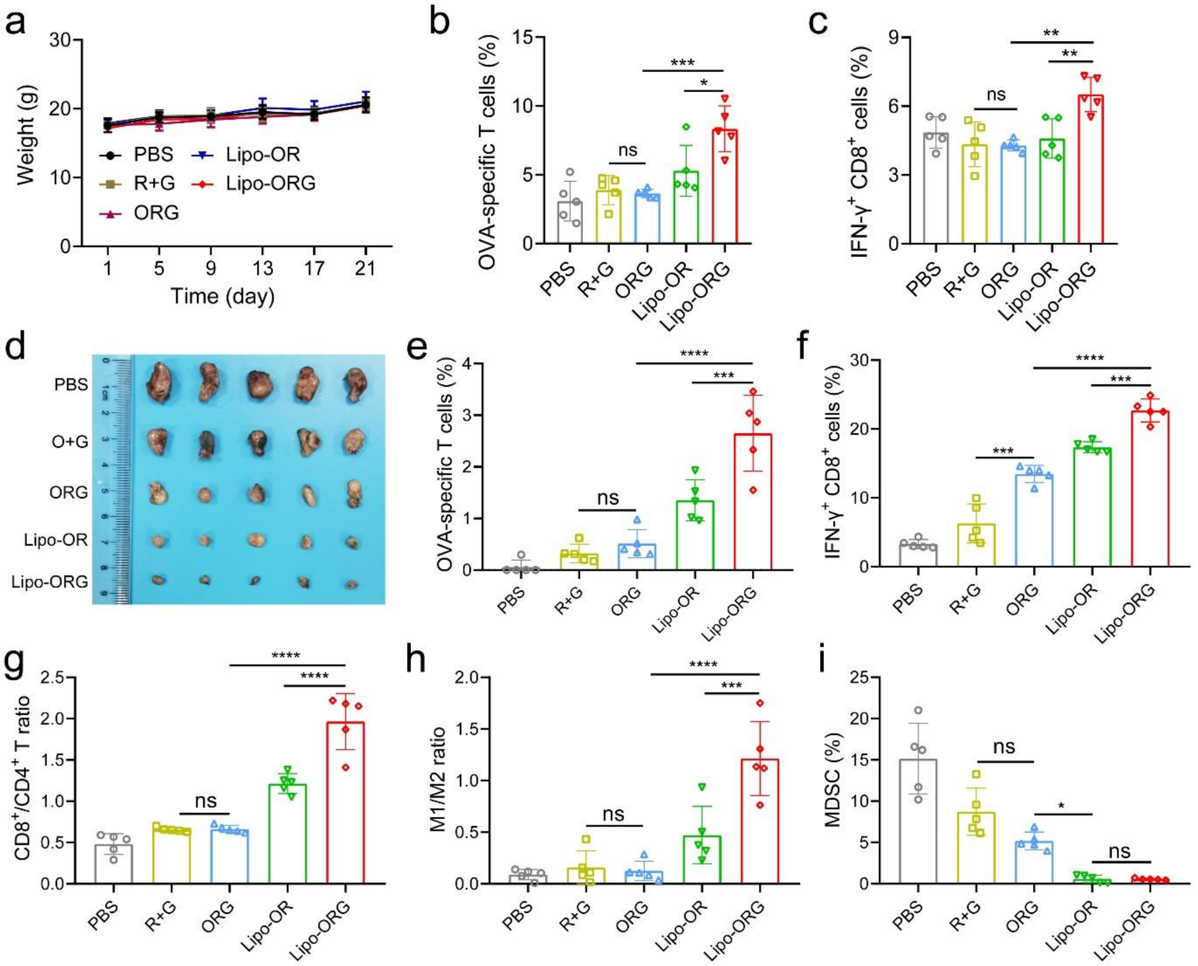

Supplementary Figure 25. (a) Body weight of B16-OVA lung metastatic mice with various treatments. (b) Populations of OVA-tetramer positive $CD8^+$ T cells, and (c) IFN-γ^+CD8^+ T cells in spleen of mice (gated on $CD8^+$ T cells). (d) Photographs of collected MC38-OVA

tumors at the end of antitumor study. (e) Population of OVA-epitope specific T cells in MC38-OVA tumors after treatments. (f) Population of IFN-γ^+ $CD8^+$ T cells in tumors. (g) $CD8^+$ to $CD4^+$ T cell ratios in tumors of control and treated groups. (h) M1 to M2 cell ratios in tumors. (i) Frequency of MDSCs in tumors after various treatments.

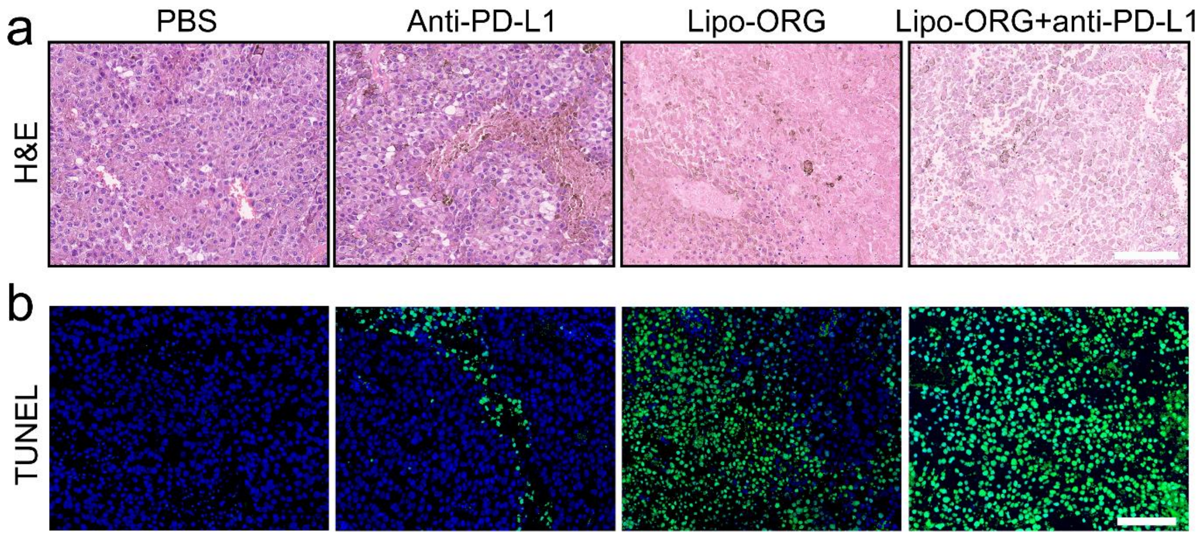

Supplementary Figure 26. (a) H&E, and (b) TUNEL staining of tumor sections at the end of antitumor study. Scale bars, 100 μm.

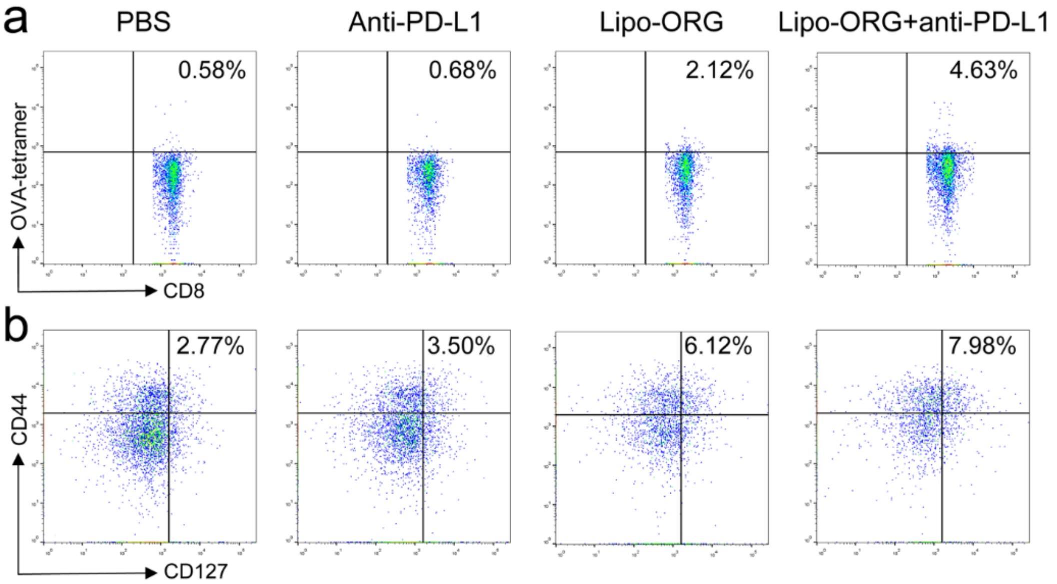

Supplementary Figure 27. Representative flow cytometry images for (a) OVA-tetramer positive $CD8^+$ T cells, and (b) $CD8^+T_{CM}$ ($CD44^+CD127^+$) in tumors.

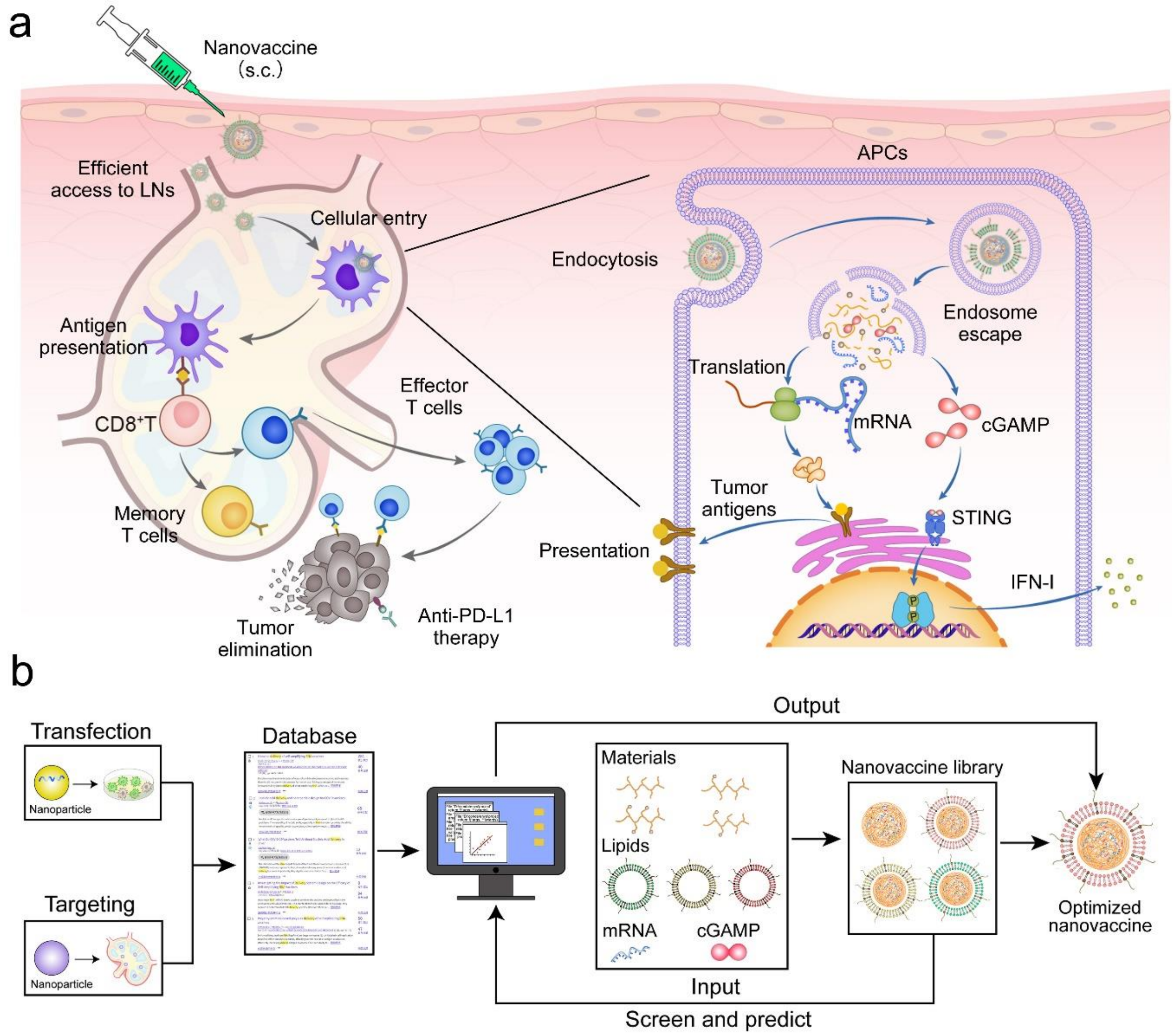

Supplementary Figure 28. Schematically illustrating the *in vivo* fate of nanovaccine for improving cancer immunotherapy. (a) Anionic Lipo-ORG enabled effective access to lymphatics and high accumulation in lymph nodes. Effective cellular entry and endosome escape of mRNA and cGAMP was achieved in DCs and potentiated antigen presentation to T cells. Tumor antigens were translated after the release of mRNA. cGAMP activated STING pathway to trigger IFN-I, which amplified the innate and adaptive immune responses. T cell inflammation and immune memory effect were initiated for tumor elimination, which was further improved when combined with anti-PD-L1 therapy. (b) A brief outlook upon further optimization of nanovaccines for LNs targeting and high transfection through machine learning techniques.

References

1. Lin YX, Wang Y, Ding J, Jiang A, Wang J, Yu M, *et al.* Reactivation of the tumor suppressor PTEN by mRNA nanoparticles enhances antitumor immunity in preclinical models. *Sci Transl Med* 2021, **13**(599).

2. Wang H, Wang Y, Wang Y, Hu J, Li T, Liu H, *et al.* Self-Assembled Fluorodendrimers Combine the Features of Lipid and Polymeric Vectors in Gene Delivery. *Angew Chem Int Ed Engl* 2015, **54**(40)**:** 11647-11651.

3. Zhang Y, Xie F, Yin Y, Zhang Q, Jin H, Wu Y, *et al.* Immunotherapy of Tumor RNA-Loaded Lipid Nanoparticles Against Hepatocellular Carcinoma. *Int J Nanomedicine* 2021, **16:** 1553-1564.

4. Sayour EJ, De Leon G, Pham C, Grippin A, Kemeny H, Chua J, *et al.* Systemic activation of antigen-presenting cells via RNA-loaded nanoparticles. *Oncoimmunology* 2017, **6**(1)**:** e1256527.

5. Hwang BJ, Jang Y, Kwon SB, Yu JE, Lim J, Roh YH, *et al.* RNA-assisted self-assembly of monomeric antigens into virus-like particles as a recombinant vaccine platform. *Biomaterials* 2021, **269:** 120650.

6. Blakney AK, Zhu Y, McKay PF, Bouton CR, Yeow J, Tang J, *et al.* Big Is Beautiful: Enhanced saRNA Delivery and Immunogenicity by a Higher Molecular Weight, Bioreducible, Cationic Polymer. *ACS Nano* 2020, **14**(5)**:** 5711-5727.

7. Perche F, Clemencon R, Schulze K, Ebensen T, Guzman CA, Pichon C. Neutral Lipopolyplexes for In Vivo Delivery of Conventional and Replicative RNA Vaccine. *Mol Ther Nucleic Acids* 2019, **17:** 767-775.

8. Englezou PC, Sapet C, Demoulins T, Milona P, Ebensen T, Schulze K, *et al.* Self-Amplifying Replicon RNA Delivery to Dendritic Cells by Cationic Lipids. *Mol Ther Nucleic Acids* 2018, **12:** 118-134.

9. Erasmus JH, Khandhar AP, Guderian J, Granger B, Archer J, Archer M, *et al.* A Nanostructured Lipid Carrier for Delivery of a Replicating Viral RNA Provides Single, Low-Dose Protection against Zika. *Mol Ther* 2018, **26**(10)**:** 2507-2522.

10. Anderluzzi G, Lou G, Gallorini S, Brazzoli M, Johnson R, O'Hagan DT, *et al.* Investigating the Impact of Delivery System Design on the Efficacy of Self-Amplifying RNA Vaccines. *Vaccines (Basel)* 2020, **8**(2).

11. Abeyratne E, Tharmarajah K, Freitas JR, Mostafavi H, Mahalingam S, Zaid A, *et al.* Liposomal Delivery of the RNA Genome of a Live-Attenuated Chikungunya Virus Vaccine Candidate Provides Local, but Not Systemic Protection After One Dose. *Front Immunol* 2020, **11:** 304.

12. Sun S, Li W, Sun Y, Pan Y, Li J. A new RNA vaccine platform based on MS2 virus-like particles produced in Saccharomyces cerevisiae. *Biochem Biophys Res Commun* 2011, **407**(1)**:** 124-128.

13. He W, Evans AC, Rasley A, Bourguet F, Peters S, Kamrud KI, *et al.* Cationic HDL mimetics enhance in vivo delivery of self-replicating mRNA. *Nanomedicine* 2020, **24:** 102154.

14. Blakney AK, McKay PF, Yus BI, Aldon Y, Shattock RJ. Inside out: optimization of lipid nanoparticle formulations for exterior complexation and in vivo delivery of saRNA. *Gene Ther* 2019, **26**(9)**:** 363-372.

15. Biddlecome A, Habte HH, McGrath KM, Sambanthamoorthy S, Wurm M, Sykora MM, *et al.* Delivery of self-amplifying RNA vaccines in in vitro reconstituted virus-like particles. *PLoS One* 2019, **14**(6)**:** e0215031.

16. Blakney AK, McKay PF, Christensen D, Yus BI, Aldon Y, Follmann F, *et al.* Effects of cationic adjuvant formulation particle type, fluidity and immunomodulators on delivery and immunogenicity of saRNA. *J Control Release* 2019, **304:** 65-74.

17. Brito LA, Chan M, Shaw CA, Hekele A, Carsillo T, Schaefer M, *et al.* A cationic nanoemulsion for the delivery of next-generation RNA vaccines. *Mol Ther* 2014, **22**(12)**:** 2118-2129.

18. Kranz LM, Diken M, Haas H, Kreiter S, Loquai C, Reuter KC, *et al.* Systemic RNA delivery to dendritic cells exploits antiviral defence for cancer immunotherapy. *Nature* 2016, **534**(7607)**:** 396-401.

19. Gurnani P, Blakney AK, Terracciano R, Petch JE, Blok AJ, Bouton CR, *et al.* The In Vitro, Ex Vivo, and In Vivo Effect of Polymer Hydrophobicity on Charge-Reversible Vectors for Self-Amplifying RNA. *Biomacromolecules* 2020, **21**(8)**:** 3242-3253.

20. McKay PF, Hu K, Blakney AK, Samnuan K, Brown JC, Penn R, *et al.* Self-amplifying RNA SARS-CoV-2 lipid nanoparticle vaccine candidate induces high neutralizing antibody titers in mice. *Nat Commun* 2020, **11**(1)**:** 3523.

21. Juanes M, Creese O, Fernandez-Trillo P, Montenegro J. Messenger RNA delivery by hydrazone-activated polymers. *Medchemcomm* 2019, **10**(7)**:** 1138-1144.

22. Miao L, Li L, Huang Y, Delcassian D, Chahal J, Han J, *et al.* Delivery of mRNA vaccines with heterocyclic lipids increases anti-tumor efficacy by STING-mediated immune cell activation. *Nat Biotechnol* 2019, **37**(10)**:** 1174-1185.

23. Sayour EJ, Grippin A, De Leon G, Stover B, Rahman M, Karachi A, *et al.* Personalized Tumor RNA Loaded Lipid-Nanoparticles Prime the Systemic and Intratumoral Milieu for Response to Cancer Immunotherapy. *Nano Lett* 2018, **18**(10)**:** 6195-6206.

24. Fan YN, Li M, Luo YL, Chen Q, Wang L, Zhang HB, *et al.* Cationic lipid-assisted nanoparticles for delivery of mRNA cancer vaccine. *Biomater Sci* 2018, **6**(11)**:** 3009-3018.

25. Blakney AK, Abdouni Y, Yilmaz G, Liu R, McKay PF, Bouton CR, *et al.* Mannosylated Poly(ethylene imine) Copolymers Enhance saRNA Uptake and Expression in Human Skin Explants. *Biomacromolecules* 2020, **21**(6)**:** 2482-2492.

26. Goswami R, Chatzikleanthous D, Lou G, Giusti F, Bonci A, Taccone M, *et al.* Mannosylation of LNP Results in Improved Potency for Self-Amplifying RNA (SAM) Vaccines. *ACS Infect Dis* 2019, **5**(9)**:** 1546-1558.

27. Lou B, De Beuckelaer A, Dakwar GR, Remaut K, Grooten J, Braeckmans K, *et al.* Post-PEGylated and crosslinked polymeric ssRNA nanocomplexes as adjuvants targeting lymph nodes with increased cytolytic T cell inducing properties. *J Control Release* 2018, **284:** 73-83.

28. McCullough KC, Bassi I, Milona P, Suter R, Thomann-Harwood L, Englezou P, *et al.* Self-replicating Replicon-RNA Delivery to Dendritic Cells by Chitosan-nanoparticles for Translation In Vitro and In Vivo. *Mol Ther Nucleic Acids* 2014, **3:** e173.

29. Su X, Fricke J, Kavanagh DG, Irvine DJ. In vitro and in vivo mRNA delivery using lipid-enveloped pH-responsive polymer nanoparticles. *Mol Pharm* 2011, **8**(3)**:** 774-787.

30. Perche F, Benvegnu T, Berchel M, Lebegue L, Pichon C, Jaffres PA, *et al.* Enhancement of dendritic cells transfection in vivo and of vaccination against B16F10 melanoma with mannosylated histidylated lipopolyplexes loaded with tumor antigen messenger RNA. *Nanomedicine* 2011, **7**(4)**:** 445-453.

31. Zhao M, Li M, Zhang Z, Gong T, Sun X. Induction of HIV-1 gag specific immune responses by cationic micelles mediated delivery of gag mRNA. *Drug Deliv* 2016, **23**(7)**:** 2596-2607.

32. Tan L, Zheng T, Li M, Zhong X, Tang Y, Qin M, *et al.* Optimization of an mRNA vaccine assisted with cyclodextrin-polyethyleneimine conjugates. *Drug Deliv Transl Res* 2020, **10**(3)**:** 678-689.

33. Zhang W, Liu Y, Min Chin J, Phua KKL. Sustained release of PKR inhibitor C16 from mesoporous silica nanoparticles significantly enhances mRNA translation and anti-tumor vaccination. *Eur J Pharm Biopharm* 2021, **163:** 179-187.

34. Paul AM, Shi Y, Acharya D, Douglas JR, Cooley A, Anderson JF, *et al.* Delivery of antiviral small interfering RNA with gold nanoparticles inhibits dengue virus infection in vitro. *J Gen Virol* 2014, **95**(Pt 8)**:** 1712-1722.

35. Coolen AL, Lacroix C, Mercier-Gouy P, Delaune E, Monge C, Exposito JY, *et al.* Poly(lactic acid) nanoparticles and cell-penetrating peptide potentiate mRNA-based vaccine expression in dendritic cells triggering their activation. *Biomaterials* 2019, **195:** 23-37.

36. Lou G, Anderluzzi G, Schmidt ST, Woods S, Gallorini S, Brazzoli M, *et al.* Delivery of self-amplifying mRNA vaccines by cationic lipid nanoparticles: The impact of cationic lipid selection. *J Control Release* 2020, **325:** 370-379.

37. Zhang H, You X, Wang X, Cui L, Wang Z, Xu F, *et al.* Delivery of mRNA vaccine with a lipid-like material potentiates antitumor efficacy through Toll-like receptor 4 signaling. *Proc Natl Acad Sci U S A* 2021, **118**(6).

38. Eygeris Y, Patel S, Jozic A, Sahay G. Deconvoluting Lipid Nanoparticle Structure for Messenger RNA Delivery. *Nano Lett* 2020, **20**(6)**:** 4543-4549.

39. Liu DQ, Lu S, Zhang LX, Ji M, Liu SY, Wang SW, *et al.* An indoleamine 2, 3-dioxygenase siRNA nanoparticle-coated and Trp2-displayed recombinant yeast vaccine inhibits melanoma tumor growth in mice. *J Control Release* 2018, **273:** 1-12.

40. Sharifnia Z, Bandehpour M, Hamishehkar H, Mosaffa N, Kazemi B, Zarghami N. In-vitro Transcribed mRNA Delivery Using PLGA/PEI Nanoparticles into Human Monocyte-derived Dendritic Cells. *Iran J Pharm Res* 2019, **18**(4)**:** 1659-1675.

41. Patel S, Ashwanikumar N, Robinson E, DuRoss A, Sun C, Murphy-Benenato KE, *et al.* Boosting Intracellular Delivery of Lipid Nanoparticle-Encapsulated mRNA. *Nano Lett* 2017, **17**(9)**:** 5711-5718.

42. Wang C, Shi X, Song H, Zhang C, Wang X, Huang P, *et al.* Polymer-lipid hybrid nanovesicle-enabled combination of immunogenic chemotherapy and RNAi-mediated PD-L1 knockdown elicits antitumor immunity against melanoma. *Biomaterials* 2021, **268:** 120579.

43. Warashina S, Nakamura T, Sato Y, Fujiwara Y, Hyodo M, Hatakeyama H, *et al.* A lipid nanoparticle for the efficient delivery of siRNA to dendritic cells. *J Control Release* 2016, **225:** 183-191.

44. Siewert CD, Haas H, Cornet V, Nogueira SS, Nawroth T, Uebbing L, *et al.* Hybrid Biopolymer and Lipid Nanoparticles with Improved Transfection Efficacy for mRNA. *Cells* 2020, **9**(9).

45. Li L, Lin SL, Deng L, Liu ZG. Potential use of chitosan nanoparticles for oral delivery of DNA vaccine in black seabream Acanthopagrus schlegelii Bleeker to protect from Vibrio parahaemolyticus. *J Fish Dis* 2013, **36**(12)**:** 987-995.

46. Yang DC, Eldredge AC, Hickey JC, Muradyan H, Guan Z. Multivalent Peptide-Functionalized Bioreducible Polymers for Cellular Delivery of Various RNAs. *Biomacromolecules* 2020, **21**(4)**:** 1613-1624.

47. Heo MB, Cho MY, Lim YT. Polymer nanoparticles for enhanced immune response: combined delivery of tumor antigen and small interference RNA for immunosuppressive gene to dendritic cells. *Acta Biomater* 2014, **10**(5)**:** 2169-2176.

48. Kim M, Jeong M, Hur S, Cho Y, Park J, Jung H, *et al.* Engineered ionizable lipid nanoparticles for targeted delivery of RNA therapeutics into different types of cells in the liver. *Sci Adv* 2021, **7**(9)**:** abf4398.

49. Le Moignic A, Malard V, Benvegnu T, Lemiegre L, Berchel M, Jaffres PA, *et al.* Preclinical evaluation of mRNA trimannosylated lipopolyplexes as therapeutic cancer vaccines targeting dendritic cells. *J Control Release* 2018, **278:** 110-121.

50. Gomez-Aguado I, Rodriguez-Castejon J, Vicente-Pascual M, Rodriguez-Gascon A, Pozo-Rodriguez AD, Solinis Aspiazu MA. Nucleic Acid Delivery by Solid Lipid Nanoparticles Containing Switchable Lipids: Plasmid DNA vs. Messenger RNA. *Molecules* 2020, **25**(24)**:** 5995.

51. Han SW, Lee JB. Self-assembly of tumor-targeting RNA nanoball for carrier-free delivery of therapeutic RNA. *Journal of Industrial and Engineering Chemistry* 2018, **64:** 90-96.

52. Lee JB, Hong J, Bonner DK, Poon Z, Hammond PT. Self-assembled RNA interference microsponges for efficient siRNA delivery. *Nat Mater* 2012, **11**(4)**:** 316-322.

53. Moon JH, Mendez E, Kim Y, Kaur A. Conjugated polymer nanoparticles for small interfering RNA delivery. *Chem Commun (Camb)* 2011, **47**(29)**:** 8370-8372.

54. Jeon H, Han S, Kim H, Lee JB. Surface modification of RNA nanoparticles by ionic interaction for efficient cellular uptake. *Journal of Industrial and Engineering Chemistry* 2019, **70:** 87-93.

55. Yin Y, Li X, Ma H, Zhang J, Yu D, Zhao R, *et al.* In Situ Transforming RNA Nanovaccines from Polyethylenimine Functionalized Graphene Oxide Hydrogel for Durable Cancer Immunotherapy. *Nano Lett* 2021, **21**(5)**:** 2224-2231.

56. Kim ST, Chompoosor A, Yeh YC, Agasti SS, Solfiell DJ, Rotello VM. Dendronized gold nanoparticles for siRNA delivery. *Small* 2012, **8**(21)**:** 3253-3256.

57. Tseng SJ, Zeng YF, Deng YF, Yang PC, Liu JR, Kempson IM. Switchable delivery of small interfering RNA using a negatively charged pH-responsive polyethylenimine-based polyelectrolyte complex. *Chem Commun (Camb)* 2013, **49**(26)**:** 2670-2672.

58. Shi J, Xiao Z, Votruba AR, Vilos C, Farokhzad OC. Differentially charged hollow core/shell lipid-polymer-lipid hybrid nanoparticles for small interfering RNA delivery. *Angew Chem Int Ed Engl* 2011, **50**(31)**:** 7027-7031.

59. Jiang Z, Cui W, Mager J, Thayumanavan S. Postfunctionalization of Noncationic RNA–Polymer Complexes for RNA Delivery. *Ind Eng Chem Res* 2019, **58**(17)**:** 6982-6991.

60. Dorasamy S, Narainpersad N, Singh M, Ariatti M. Novel targeted liposomes deliver sirna to hepatocellular carcinoma cells in vitro. *Chem Biol Drug Des* 2012, **80**(5)**:** 647-656.

61. Yang M, Jin H, Chen J, Ding L, Ng KK, Lin Q, *et al.* Efficient cytosolic delivery of siRNA using HDL-mimicking nanoparticles. *Small* 2011, **7**(5)**:** 568-573.

62. Chen M, Zhang X-Q, Man HB, Lam R, Chow EK, Ho D. Nanodiamond Vectors Functionalized with Polyethylenimine for siRNA Delivery. *The Journal of Physical Chemistry Letters* 2010, **1**(21)**:** 3167-3171.

63. Kim SW, Kim NY, Choi YB, Park SH, Yang JM, Shin S. RNA interference in vitro and in vivo using an arginine peptide/siRNA complex system. *J Control Release* 2010, **143**(3)**:** 335-343.

64. Fenton OS, Kauffman KJ, McClellan RL, Appel EA, Dorkin JR, Tibbitt MW, *et al.* Bioinspired Alkenyl Amino Alcohol Ionizable Lipid Materials for Highly Potent In Vivo mRNA Delivery. *Advanced Materials* 2016, **28**(15)**:** 2939-2943.

65. Huang X, Lai Y, Braun GB, Reich NO. Modularized Gold Nanocarriers for TAT-Mediated Delivery of siRNA. *Small* 2017, **13**(8)**:** 1602473.

66. McMahon KM, Plebanek MP, Thaxton CS. Properties of Native High-Density Lipoproteins Inspire Synthesis of Actively Targeted In Vivo siRNA Delivery Vehicles. *Adv Funct Mater* 2016, **26**(43)**:** 7824-7835.

67. Jang M, Kim JH, Nam HY, Kwon IC, Ahn HJ. Design of a platform technology for systemic delivery of siRNA to tumours using rolling circle transcription. *Nat Commun* 2015, **6:** 7930.

68. Karlsson J, Tzeng SY, Hemmati S, Luly KM, Choi O, Rui Y, *et al.* Photocrosslinked Bioreducible Polymeric Nanoparticles for Enhanced Systemic siRNA Delivery as Cancer Therapy. *Adv Funct Mater* 2021, **31**(17)**:** 2009768.

69. Kim H, Lee JS, Lee JB. Generation of siRNA Nanosheets for Efficient RNA Interference. *Sci Rep* 2016, **6:** 25146.

70. Sajeesh S, Lee TY, Kim JK, Son DS, Hong SW, Kim S, *et al.* Efficient intracellular delivery and multiple-target gene silencing triggered by tripodal RNA based nanoparticles: a promising approach in liver-specific RNAi delivery. *J Control Release* 2014, **196:** 28-36.

71. Grabowska M, Grzeskowiak BF, Szutkowski K, Wawrzyniak D, Glodowicz P, Barciszewski J, *et al.* Nano-mediated delivery of double-stranded RNA for gene therapy of glioblastoma multiforme. *PLoS One* 2019, **14**(3)**:** e0213852.

72. Park Y, Kim H, Lee JB. Self-assembled DNA-Guided RNA Nanovector via Step-wise Dual Enzyme Polymerization (SDEP) for Carrier-free siRNA Delivery. *Acs Biomater Sci Eng* 2016, **2**(4)**:** 616-624.

73. Tang Q, Cao B, Cheng G. Co-delivery of small interfering RNA using a camptothecin prodrug as the carrier. *Chem Commun (Camb)* 2014, **50**(11)**:** 1323-1325.

74. Liu C, Wen J, Meng Y, Zhang K, Zhu J, Ren Y, *et al.* Efficient delivery of therapeutic miRNA nanocapsules for tumor suppression. *Adv Mater* 2015, **27**(2)**:** 292-297.

75. Li B, Luo X, Deng B, Giancola JB, McComb DW, Schmittgen TD, *et al.* Effects of local structural transformation of lipid-like compounds on delivery of messenger RNA. *Sci Rep* 2016, **6:** 22137.

76. Zhang Y, Pelet JM, Heller DA, Dong Y, Chen D, Gu Z, *et al.* Lipid-modified aminoglycoside derivatives for in vivo siRNA delivery. *Adv Mater* 2013, **25**(33)**:** 4641-4645.

77. Fenton OS, Kauffman KJ, McClellan RL, Kaczmarek JC, Zeng MD, Andresen JL, *et al.* Customizable Lipid Nanoparticle Materials for the Delivery of siRNAs and mRNAs. *Angew Chem Int Ed Engl* 2018, **57**(41)**:** 13582-13586.

78. Hom C, Lu J, Liong M, Luo H, Li Z, Zink JI, *et al.* Mesoporous silica nanoparticles facilitate delivery of siRNA to shutdown signaling pathways in mammalian cells. *Small* 2010, **6**(11)**:** 1185-1190.

79. Wang F, Zhang W, Shen Y, Huang Q, Zhou D, Guo S. Efficient RNA delivery by integrin-targeted glutathione responsive polyethyleneimine capped gold nanorods. *Acta Biomater* 2015, **23:** 136-146.

80. Deshayes S, Konate K, Rydstrom A, Crombez L, Godefroy C, Milhiet PE, *et al.* Self-assembling peptide-based nanoparticles for siRNA delivery in primary cell lines. *Small* 2012, **8**(14)**:** 2184-2188.

81. Zhou K, Kos P, Yan Y, Xiong H, Min YL, Kinghorn KA, *et al.* Intercalation-mediated nucleic acid nanoparticles for siRNA delivery. *Chem Commun (Camb)* 2016, **52**(82)**:** 12155-12158.

82. Khan OF, Zaia EW, Jhunjhunwala S, Xue W, Cai W, Yun DS, *et al.* Dendrimer-Inspired Nanomaterials for the in Vivo Delivery of siRNA to Lung Vasculature. *Nano Lett* 2015, **15**(5)**:** 3008-3016.

83. Na HK, Kim MH, Park K, Ryoo SR, Lee KE, Jeon H, *et al.* Efficient functional delivery of siRNA using mesoporous silica nanoparticles with ultralarge pores. *Small* 2012, **8**(11)**:** 1752-1761.

84. Yu B, Hsu SH, Zhou C, Wang X, Terp MC, Wu Y, *et al.* Lipid nanoparticles for hepatic delivery of small interfering RNA. *Biomaterials* 2012, **33**(25)**:** 5924-5934.

85. Shim MS, Bhang SH, Yoon K, Choi K, Xia Y. A bioreducible polymer for efficient delivery of Fas-silencing siRNA into stem cell spheroids and enhanced therapeutic angiogenesis. *Angew Chem Int Ed Engl* 2012, **51**(47)**:** 11899-11903.

86. Chen L, She X, Wang T, Shigdar S, Duan W, Kong L. Mesoporous silica nanorods toward efficient loading and intracellular delivery of siRNA. *J Nanopart Res* 2018, **20**(2)**:** 37.

87. Choi KY, Silvestre OF, Huang X, Hida N, Liu G, Ho DN, *et al.* A nanoparticle formula for delivering siRNA or miRNAs to tumor cells in cell culture and in vivo. *Nat Protoc* 2014, **9**(8)**:** 1900-1915.

88. Qi L, Wu L, Zheng S, Wang Y, Fu H, Cui D. Cell-penetrating magnetic nanoparticles for highly efficient delivery and intracellular imaging of siRNA. *Biomacromolecules* 2012, **13**(9)**:** 2723-2730.

89. McCaskill J, Singhania R, Burgess M, Allavena R, Wu S, Blumenthal A, *et al.* Efficient Biodistribution and Gene Silencing in the Lung epithelium via Intravenous Liposomal Delivery of siRNA. *Mol Ther Nucleic Acids* 2013, **2:** e96.

90. Miller JB, Kos P, Tieu V, Zhou K, Siegwart DJ. Development of Cationic Quaternary Ammonium Sulfonamide Amino Lipids for Nucleic Acid Delivery. *ACS Appl Mater Interfaces* 2018, **10**(3)**:** 2302-2311.

91. Brissault B, Leborgne C, Scherman D, Guis C, Kichler A. Synthesis of poly(propylene glycol)-block-polyethylenimine triblock copolymers for the delivery of nucleic acids. *Macromol Biosci* 2011, **11**(5)**:** 652-661.

92. Li H-J, Wang H-X, Sun C-Y, Du J-Z, Wang J. Shell-detachable nanoparticles based on a light-responsive amphiphile for enhanced siRNA delivery. *RSC Adv* 2014, **4**(4)**:** 1961-1964.

93. Ewe A, Noske S, Karimov M, Aigner A. Polymeric Nanoparticles Based on Tyrosine-Modified, Low Molecular Weight Polyethylenimines for siRNA Delivery. *Pharmaceutics* 2019, **11**(11)**:** 600.

94. Kang SH, Cho HJ, Shim GY, Lee SB, Kim SH, Choi HG, *et al*. Cationic Liposomal Co-delivery of Small Interfering RNA and a MEK Inhibitor for Enhanced Anticancer Efficacy. *Pharm Res* 2011, **28:** 3069-3078.

95. Choi KY, Silvestre OF, Huang X, Min KH, Howard GP, Hida N, *et al.* Versatile RNA interference nanoplatform for systemic delivery of RNAs. *ACS Nano* 2014, **8**(5)**:** 4559-4570.

96. Luo X, Wang W, Dorkin JR, Veiseh O, Chang PH, Abutbul-Ionita I, *et al.* Poly(glycoamidoamine) brush nanomaterials for systemic siRNA delivery in vivo. *Biomater Sci* 2016, **5**(1)**:** 38-40.

97. Lu JM, Liang Z, Wang X, Gu J, Yao Q, Chen C. New polymer of lactic-co-glycolic acid-modified polyethylenimine for nucleic acid delivery. *Nanomedicine (Lond)* 2016, **11**(15)**:** 1971-1991.

98. Gilleron J, Querbes W, Zeigerer A, Borodovsky A, Marsico G, Schubert U, *et al.* Image-based analysis of lipid nanoparticle-mediated siRNA delivery, intracellular trafficking and endosomal escape. *Nat Biotechnol* 2013, **31**(7)**:** 638-646.

99. Song WJ, Du JZ, Sun TM, Zhang PZ, Wang J. Gold nanoparticles capped with polyethyleneimine for enhanced siRNA delivery. *Small* 2010, **6**(2)**:** 239-246.

100. Wu C, Li J, Wang W, Hammond PT. Rationally Designed Polycationic Carriers for Potent Polymeric siRNA-Mediated Gene Silencing. *ACS Nano* 2018, **12**(7)**:** 6504-6514.

101. Baigude H, Su J, McCarroll J, Rana TM. In Vivo Delivery of RNAi by Reducible Interfering Nanoparticles (iNOPs). *ACS Med Chem Lett* 2013, **4**(8)**:** 720-723.

102. Yang XZ, Du JZ, Dou S, Mao CQ, Long HY, Wang J. Sheddable ternary nanoparticles for tumor acidity-targeted siRNA delivery. *ACS Nano* 2012, **6**(1)**:** 771-781.

103. Cai X, Zhu H, Zhang Y, Gu Z. Highly Efficient and Safe Delivery of VEGF siRNA by Bioreducible Fluorinated Peptide Dendrimers for Cancer Therapy. *ACS Appl Mater Interfaces* 2017, **9**(11)**:** 9402-9415.

104. Zeng H, Schlesener C, Cromwell O, Hellmund M, Haag R, Guan Z. Amino Acid-Functionalized Dendritic Polyglycerol for Safe and Effective siRNA Delivery. *Biomacromolecules* 2015, **16**(12)**:** 3869-3877.

105. Tieu T, Dhawan S, Haridas V, Butler LM, Thissen H, Cifuentes-Rius A, *et al.* Maximizing RNA Loading for Gene Silencing Using Porous Silicon Nanoparticles. *ACS Appl Mater Interfaces* 2019, **11**(26)**:** 22993-23005.

106. Chen G, Wang K, Wu P, Wang Y, Zhou Z, Yin L, *et al.* Development of fluorinated polyplex nanoemulsions for improved small interfering RNA delivery and cancer therapy. *Nano Res* 2018, **11**(7)**:** 3746-3761.

107. Palanca-Wessels MC, Booth GC, Convertine AJ, Lundy BB, Berguig GY, Press MF, *et al.* Antibody targeting facilitates effective intratumoral siRNA nanoparticle delivery to HER2-overexpressing cancer cells. *Oncotarget* 2016, **7**(8)**:** 9561-9575.

108. Wang X, Sun Q, Cui C, Li J, Wang Y. Anti-HER2 functionalized graphene oxide as survivin-siRNA delivery carrier inhibits breast carcinoma growth in vitro and in vivo. *Drug Des Devel Ther* 2018, **12:** 2841-2855.

109. Islam MA, Xu Y, Tao W, Ubellacker JM, Lim M, Aum D, *et al.* Restoration of tumour-growth suppression in vivo via systemic nanoparticle-mediated delivery of PTEN mRNA. *Nat Biomed Eng* 2018, **2**(11)**:** 850-864.

110. Kong N, Tao W, Ling X, Wang J, Xiao Y, Shi S, *et al.* Synthetic mRNA nanoparticle-mediated restoration of p53 tumor suppressor sensitizes p53-deficient cancers to mTOR inhibition. *Sci Transl Med* 2019, **11**(523)**:** aaw1565.

111. Wang D, Wang T, Liu J, Yu H, Jiao S, Feng B, *et al.* Acid-Activatable Versatile Micelleplexes for PD-L1 Blockade-Enhanced Cancer Photodynamic Immunotherapy. *Nano Lett* 2016, **16**(9)**:** 5503-5513.

112. Tseng YC, Xu Z, Guley K, Yuan H, Huang L. Lipid-calcium phosphate nanoparticles for delivery to the lymphatic system and SPECT/CT imaging of lymph node metastases. *Biomaterials* 2014, **35**(16)**:** 4688-4698.

113. Zhang YN, Lazarovits J, Poon W, Ouyang B, Nguyen LNM, Kingston BR, *et al.* Nanoparticle Size Influences Antigen Retention and Presentation in Lymph Node Follicles for Humoral Immunity. *Nano Lett* 2019, **19**(10)**:** 7226-7235.

114. Lee IH, Kwon HK, An S, Kim D, Kim S, Yu MK, *et al.* Imageable antigen-presenting gold nanoparticle vaccines for effective cancer immunotherapy in vivo. *Angew Chem Int Ed Engl* 2012, **51**(35)**:** 8800-8805.

115. Mohammad AK, Amayreh LK, Mazzara JM, Reineke JJ. Rapid lymph accumulation of polystyrene nanoparticles following pulmonary administration. *Pharm Res* 2013, **30**(2)**:** 424-434.

116. Li N, Peng LH, Chen X, Zhang TY, Shao GF, Liang WQ*, et al.* Antigen-loaded nanocarriers enhance the migration of stimulated Langerhans cells to draining lymph nodes and induce effective transcutaneous immunization. *Nanomedicine* 2014, **10**(1)**:** 215-223.

117. Mottas I, Bekdemir A, Cereghetti A, Spagnuolo L, Yang YS, Muller M*, et al.* Amphiphilic nanoparticle delivery enhances the anticancer efficacy of a TLR7 ligand via local immune activation. *Biomaterials* 2019, **190-191:** 111-120.

118. Ye T, Xu W, Shi T, Yang R, Yang X, Wang S*, et al.* Targeted delivery of docetaxel to the metastatic lymph nodes: A comparison study between nanoliposomes and activated carbon nanoparticles. *Asian Journal of Pharmaceutical Sciences* 2015, **10**(1)**:** 64-72.

119. Mueller SN, Tian S, DeSimone JM. Rapid and Persistent Delivery of Antigen by Lymph Node Targeting PRINT Nanoparticle Vaccine Carrier To Promote Humoral Immunity. *Mol Pharm* 2015, **12**(5)**:** 1356-1365.

120. Wu X, Li Y, Chen X, Zhou Z, Pang J, Luo X*, et al.* A surface charge dependent enhanced Th1 antigen-specific immune response in lymph nodes by transfersome-based nanovaccine-loaded dissolving microneedle-assisted transdermal immunization. *J Mater Chem B* 2019, **7**(31)**:** 4854-4866.

121. An M, Li M, Xi J, Liu H. Silica Nanoparticle as a Lymph Node Targeting Platform for Vaccine Delivery. *ACS Appl Mater Interfaces* 2017, **9**(28)**:** 23466-23475.

122. Cabral H, Makino J, Matsumoto Y, Mi P, Wu H, Nomoto T*, et al.* Systemic Targeting of Lymph Node Metastasis through the Blood Vascular System by Using Size-Controlled Nanocarriers. *ACS Nano* 2015, **9**(5)**:** 4957-4967.

123. Khullar OV, Griset AP, Gibbs-Strauss SL, Chirieac LR, Zubris KA, Frangioni JV*, et al.* Nanoparticle migration and delivery of Paclitaxel to regional lymph nodes in a large animal model. *J Am Coll Surg* 2012, **214**(3)**:** 328-337.

124. Sestito LF, Thomas SN. Lymph-directed nitric oxide increases immune cell access to lymph-borne nanoscale solutes. *Biomaterials* 2021, **265:** 120411.

125. Xiang J, Xu L, Gong H, Zhu W, Wang C, Xu J*, et al.* Antigen-Loaded Upconversion Nanoparticles for Dendritic Cell Stimulation, Tracking, and Vaccination in Dendritic Cell-Based Immunotherapy. *ACS Nano* 2015, **9**(6)**:** 6401-6411.

126. Kim D, Wu Y, Shim G, Oh YK. Lipid Nanoparticle-Mediated Lymphatic Delivery of Immunostimulatory Nucleic Acids. *Pharmaceutics* 2021, **13**(4)**:** 490.

127. Qian Y, Jin H, Qiao S, Dai Y, Huang C, Lu L*, et al.* Targeting dendritic cells in lymph node with an antigen peptide-based nanovaccine for cancer immunotherapy. *Biomaterials* 2016, **98:** 171-183.

128. Bumb A, Regino CA, Egen JG, Bernardo M, Dobson PJ, Germain RN*, et al.* Trafficking of a dual-modality magnetic resonance and fluorescence imaging superparamagnetic iron oxide-based nanoprobe to lymph nodes. *Mol Imaging Biol* 2011, **13**(6)**:** 1163-1172.

129. Muraoka D, Harada N, Hayashi T, Tahara Y, Momose F, Sawada S*, et al.* Nanogel-based immunologically stealth vaccine targets macrophages in the medulla of lymph node and induces potent antitumor immunity. *ACS Nano* 2014, **8**(9)**:** 9209-9218.

130. Liu J, Li HJ, Luo YL, Xu CF, Du XJ, Du JZ, *et al.* Enhanced Primary Tumor Penetration Facilitates Nanoparticle Draining into Lymph Nodes after Systemic Injection for Tumor Metastasis Inhibition. *ACS Nano* 2019, **13**(8)**:** 8648-8658.

131. Wang C, Sun B, Bao H, Wang T, Xu W, Sun P, *et al.* NIR-II probe modified by poly(L-lysine) with efficient ovalbumin delivery for dendritic cell tracking. *Science China Chemistry* 2020, **63**(9)**:** 1272-1280.

132. Song T, Xia Y, Du Y, Chen MW, Qing H, Ma G. Engineering the Deformability of Albumin-Stabilized Emulsions for Lymph-Node Vaccine Delivery. *Adv Mater* 2021, **33**(26)**:** e2100106.

133. Nuhn L, Vanparijs N, De Beuckelaer A, Lybaert L, Verstraete G, Deswarte K, *et al.* pH-degradable imidazoquinoline-ligated nanogels for lymph node-focused immune activation. *Proc Natl Acad Sci U S A* 2016, **113**(29)**:** 8098-8103.

134. Park J, Ramanathan R, Pham L, Woodrow KA. Chitosan enhances nanoparticle delivery from the reproductive tract to target draining lymphoid organs. *Nanomedicine* 2017, **13**(6)**:** 2015-2025.

135. Da Silva CG, Camps MGM, Li T, Chan AB, Ossendorp F, Cruz LJ. Co-delivery of immunomodulators in biodegradable nanoparticles improves therapeutic efficacy of cancer vaccines. *Biomaterials* 2019, **220:** 119417.

136. Widmer J, Thauvin C, Mottas I, Nguyen VN, Delie F, Allemann E, *et al.* Polymer-based nanoparticles loaded with a TLR7 ligand to target the lymph node for immunostimulation. *Int J Pharm* 2018, **535**(1-2)**:** 444-451.

137. Mei L, Rao J, Liu Y, Li M, Zhang Z, He Q. Effective treatment of the primary tumor and lymph node metastasis by polymeric micelles with variable particle sizes. *J Control Release* 2018, **292:** 67-77.

138. Luo G, Yu X, Jin C, Yang F, Fu D, Long J, *et al.* LyP-1-conjugated nanoparticles for targeting drug delivery to lymphatic metastatic tumors. *Int J Pharm* 2010, **385**(1-2)**:** 150-156.

139. Zhou Q, Zhang Y, Du J, Li Y, Zhou Y, Fu Q, *et al.* Different-Sized Gold Nanoparticle Activator/Antigen Increases Dendritic Cells Accumulation in Liver-Draining Lymph Nodes and CD8+ T Cell Responses. *ACS Nano* 2016, **10**(2)**:** 2678-2692.

140. Bahmani B, Uehara M, Jiang L, Ordikhani F, Banouni N, Ichimura T, *et al.* Targeted delivery of immune therapeutics to lymph nodes prolongs cardiac allograft survival. *J Clin Invest* 2018, **128**(11)**:** 4770-4786.

141. Nakamura T, Kawai M, Sato Y, Maeki M, Tokeshi M, Harashima H. The Effect of Size and Charge of Lipid Nanoparticles Prepared by Microfluidic Mixing on Their Lymph Node Transitivity and Distribution. *Mol Pharm* 2020, **17**(3)**:** 944-953.

142. Howard GP, Verma G, Ke X, Thayer WM, Hamerly T, Baxter VK, *et al.* Critical Size Limit of Biodegradable Nanoparticles for Enhanced Lymph Node Trafficking and Paracortex Penetration. *Nano Res* 2019, **12**(4)**:** 837-844.

143. Kang S, Ahn S, Lee J, Kim JY, Choi M, Gujrati V, *et al.* Effects of gold nanoparticle-based vaccine size on lymph node delivery and cytotoxic T-lymphocyte responses. *J Control Release* 2017, **256:** 56-67.

144. Molino NM, Neek M, Tucker JA, Nelson EL, Wang SW. Display of DNA on Nanoparticles for Targeting Antigen Presenting Cells. *Acs Biomater Sci Eng* 2017, **3**(4)**:** 496-501.

145. Rahimian S, Kleinovink JW, Fransen MF, Mezzanotte L, Gold H, Wisse P, *et al.* Near-infrared labeled, ovalbumin loaded polymeric nanoparticles based on a hydrophilic polyester as model vaccine: In vivo tracking and evaluation of antigen-specific CD8(+) T cell immune response. *Biomaterials* 2015, **37:** 469-477.

146. Song C, Phuengkham H, Kim SY, Lee MS, Jeong JH, Shin SJ, *et al.* Aminated nanomicelles as a designer vaccine adjuvant to trigger inflammasomes and multiple arms of the innate immune response in lymph nodes. *Int J Nanomedicine* 2017, **12:** 7501-7517.

147. Nuhn L, De Koker S, Van Lint S, Zhong Z, Catani JP, Combes F, *et al.* Nanoparticle-Conjugate TLR7/8 Agonist Localized Immunotherapy Provokes Safe Antitumoral Responses. *Adv Mater* 2018, **30**(45)**:** e1803397.

148. Subramanian S, Pandey U, Gugulothu D, Patravale V, Samuel G. Modification of PLGA nanoparticles for improved properties as a 99mTc-labeled agent in sentinel lymph node detection. *Cancer Biother Radiopharm* 2013, **28**(8)**:** 598-606.

149. Wang W, Liu Z, Zhou X, Guo Z, Zhang J, Zhu P, *et al.* Ferritin nanoparticle-based SpyTag/SpyCatcher-enabled click vaccine for tumor immunotherapy. *Nanomedicine* 2019, **16:** 69-78.

150. Cho NH, Cheong TC, Min JH, Wu JH, Lee SJ, Kim D, *et al.* A multifunctional core-shell nanoparticle for dendritic cell-based cancer immunotherapy. *Nat Nanotechnol* 2011, **6**(10)**:** 675-682.

151. Yang YS, Atukorale PU, Moynihan KD, Bekdemir A, Rakhra K, Tang L, *et al.* High-throughput quantitation of inorganic nanoparticle biodistribution at the single-cell level using mass cytometry. *Nat Commun* 2017, **8:** 14069.

152. Cobaleda-Siles M, Henriksen-Lacey M, Ruiz de Angulo A, Bernecker A, Gomez Vallejo V, Szczupak B, *et al.* An iron oxide nanocarrier for dsRNA to target lymph nodes and strongly activate cells of the immune system. *Small* 2014, **10**(24)**:** 5054-5067.

153. Deng C, Chen Y, Zhang L, Wu Y, Li H, Wu Y, *et al.* Delivery of FK506-loaded PLGA nanoparticles prolongs cardiac allograft survival. *Int J Pharm* 2020, **575:** 118951.

154. Zeng Q, Li H, Jiang H, Yu J, Wang Y, Ke H, *et al.* Tailoring polymeric hybrid micelles with lymph node targeting ability to improve the potency of cancer vaccines. *Biomaterials* 2017, **122:** 105-113.

155. Masoudi A, Madaah Hosseini HR, Shokrgozar MA, Ahmadi R, Oghabian MA. The effect of poly(ethylene glycol) coating on colloidal stability of superparamagnetic iron oxide nanoparticles as potential MRI contrast agent. *Int J Pharm* 2012, **433**(1-2)**:** 129-141.

156. Sloat BR, Sandoval MA, Hau AM, He Y, Cui Z. Strong antibody responses induced by protein antigens conjugated onto the surface of lecithin-based nanoparticles. *J Control Release* 2010, **141**(1)**:** 93-100.

157. Ruiz-de-Angulo A, Zabaleta A, Gomez-Vallejo V, Llop J, Mareque-Rivas JC. Microdosed Lipid-Coated (67)Ga-Magnetite Enhances Antigen-Specific Immunity by Image Tracked Delivery of Antigen and CpG to Lymph Nodes. *ACS Nano* 2016, **10**(1)**:** 1602-1618.

158. Li P, Shi G, Zhang X, Song H, Zhang C, Wang W, *et al.* Guanidinylated cationic nanoparticles as robust protein antigen delivery systems and adjuvants for promoting antigen-specific immune responses in vivo. *J Mater Chem B* 2016, **4**(33)**:** 5608-5620.

159. Jiang H, Wang Q, Li L, Zeng Q, Li H, Gong T, *et al.* Turning the Old Adjuvant from Gel to Nanoparticles to Amplify CD8(+) T Cell Responses. *Adv Sci (Weinh)* 2018, **5**(1)**:** 1700426.

160. Yu X, Dai Y, Zhao Y, Qi S, Liu L, Lu L, *et al.* Melittin-lipid nanoparticles target to lymph nodes and elicit a systemic anti-tumor immune response. *Nat Commun* 2020, **11**(1)**:** 1110.

161. Jin H, Qian Y, Dai Y, Qiao S, Huang C, Lu L, *et al.* Magnetic Enrichment of Dendritic Cell Vaccine in Lymph Node with Fluorescent-Magnetic Nanoparticles Enhanced Cancer Immunotherapy. *Theranostics* 2016, **6**(11)**:** 2000-2014.

162. Sanz-Ortega L, Rojas JM, Marcos A, Portilla Y, Stein JV, Barber DF. T cells loaded with magnetic nanoparticles are retained in peripheral lymph nodes by the application of a magnetic field. *J Nanobiotechnology* 2019, **17**(1)**:** 14.

163. Sano K, Iwamiya Y, Kurosaki T, Ogawa M, Magata Y, Sasaki H, *et al.* Radiolabeled gamma-polyglutamic acid complex as a nano-platform for sentinel lymph node imaging. *J Control Release* 2014, **194:** 310-315.

164. Miura R, Tahara Y, Sawada SI, Sasaki Y, Akiyoshi K. Structural effects and lymphocyte activation properties of self-assembled polysaccharide nanogels for effective antigen delivery. *Sci Rep* 2018, **8**(1)**:** 16464.

165. Li C, Zhang X, Chen Q, Zhang J, Li W, Hu H, *et al.* Synthetic Polymeric Mixed Micelles Targeting Lymph Nodes Trigger Enhanced Cellular and Humoral Immune Responses. *ACS Appl Mater Interfaces* 2018, **10**(3)**:** 2874-2889.

166. Eby JK, Dane KY, O'Neil CP, Hirosue S, Swartz MA, Hubbell JA. Polymer micelles with pyridyl disulfide-coupled antigen travel through lymphatics and show enhanced cellular responses following immunization. *Acta Biomater* 2012, **8**(9)**:** 3210-3217.

167. Yang Z, Tian R, Wu J, Fan Q, Yung BC, Niu G, *et al.* Impact of Semiconducting Perylene Diimide Nanoparticle Size on Lymph Node Mapping and Cancer Imaging. *ACS Nano* 2017, **11**(4)**:** 4247-4255.

168. Singh RK, Malosse C, Davies J, Malissen B, Kochba E, Levin Y, *et al.* Using gold nanoparticles for enhanced intradermal delivery of poorly soluble auto-antigenic peptides. *Nanomedicine* 2021, **32:** 102321.

169. Qiao D, Liu L, Chen Y, Xue C, Gao Q, Mao HQ, *et al.* Potency of a Scalable Nanoparticulate Subunit Vaccine. *Nano Lett* 2018, **18**(5)**:** 3007-3016.

170. Kuai R, Sun X, Yuan W, Xu Y, Schwendeman A, Moon JJ. Subcutaneous Nanodisc Vaccination with Neoantigens for Combination Cancer Immunotherapy. *Bioconjug Chem* 2018, **29**(3)**:** 771-775.

171. Wu L, Cai X, Nelson K, Xing W, Xia J, Zhang R, *et al.* A Green Synthesis of Carbon Nanoparticle from Honey for Real-Time Photoacoustic Imaging. *Nano Res* 2013, **6**(5)**:** 312-325.

172. Son S, Nam J, Zenkov I, Ochyl LJ, Xu Y, Scheetz L, *et al.* Sugar-Nanocapsules Imprinted with Microbial Molecular Patterns for mRNA Vaccination. *Nano Lett* 2020, **20**(3)**:** 1499-1509.

173. Li H, Li Y, Wang X, Hou Y, Hong X, Gong T, *et al.* Rational design of Polymeric Hybrid Micelles to Overcome Lymphatic and Intracellular Delivery Barriers in Cancer Immunotherapy. *Theranostics* 2017, **7**(18)**:** 4383-4398.

174. Zhou S, Huang Y, Chen Y, Liu S, Xu M, Jiang T, *et al.* Engineering ApoE3-incorporated biomimetic nanoparticle for efficient vaccine delivery to dendritic cells via macropinocytosis to enhance cancer immunotherapy. *Biomaterials* 2020, **235:** 119795.

175. Hirosue S, Kourtis IC, van der Vlies AJ, Hubbell JA, Swartz MA. Antigen delivery to dendritic cells by poly(propylene sulfide) nanoparticles with disulfide conjugated peptides: Cross-presentation and T cell activation. *Vaccine* 2010, **28**(50)**:** 7897-7906.

176. Gao W, Fang RH, Thamphiwatana S, Luk BT, Li J, Angsantikul P, *et al.* Modulating antibacterial immunity via bacterial membrane-coated nanoparticles. *Nano Lett* 2015, **15**(2)**:** 1403-1409.

177. Chua BY, Al Kobaisi M, Zeng W, Mainwaring D, Jackson DC. Chitosan microparticles and nanoparticles as biocompatible delivery vehicles for peptide and protein-based immunocontraceptive vaccines. *Mol Pharm* 2012, **9**(1)**:** 81-90.

178. Cruz LJ, Tacken PJ, Zeelenberg IS, Srinivas M, Bonetto F, Weigelin B, *et al.* Tracking targeted bimodal nanovaccines: immune responses and routing in cells, tissue, and whole organism. *Mol Pharm* 2014, **11**(12)**:** 4299-4313.

179. Zhang Q, Huang W, Yuan M, Li W, Hua L, Yang Z, *et al.* Employing ATP as a New Adjuvant Promotes the Induction of Robust Antitumor Cellular Immunity by a PLGA Nanoparticle Vaccine. *ACS Appl Mater Interfaces* 2020, **12**(49)**:** 54399-54414.

180. Li S, Feng X, Wang J, Xu W, Islam MA, Sun T, *et al.* Multiantigenic Nanoformulations Activate Anticancer Immunity Depending on Size. *Adv Funct Mater* 2019, **29**(49)**:** 1903391.

181. Wagner J, Gossl D, Ustyanovska N, Xiong M, Hauser D, Zhuzhgova O, *et al.* Mesoporous Silica Nanoparticles as pH-Responsive Carrier for the Immune-Activating Drug Resiquimod Enhance the Local Immune Response in Mice. *ACS Nano* 2021, **15**(3)**:** 4450-4466.

182. Luo M, Wang H, Wang Z, Cai H, Lu Z, Li Y, *et al.* A STING-activating nanovaccine for cancer immunotherapy. *Nat Nanotechnol* 2017, **12**(7)**:** 648-654.

183. Zhu D, Hu C, Fan F, Qin Y, Huang C, Zhang Z, *et al.* Co-delivery of antigen and dual agonists by programmed mannose-targeted cationic lipid-hybrid polymersomes for enhanced vaccination. *Biomaterials* 2019, **206:** 25-40.

184. Kim H, Niu L, Larson P, Kucaba TA, Murphy KA, James BR, *et al.* Polymeric nanoparticles encapsulating novel TLR7/8 agonists as immunostimulatory adjuvants for enhanced cancer immunotherapy. *Biomaterials* 2018, **164:** 38-53.

185. Xu Z, Ramishetti S, Tseng YC, Guo S, Wang Y, Huang L. Multifunctional nanoparticles co-delivering Trp2 peptide and CpG adjuvant induce potent cytotoxic T-lymphocyte response against melanoma and its lung metastasis. *J Control Release* 2013, **172**(1)**:** 259-265.

186. Zaric M, Lyubomska O, Touzelet O, Poux C, Al-Zahrani S, Fay F, *et al.* Skin dendritic cell targeting via microneedle arrays laden with antigen-encapsulated poly-D,L-lactide-co-glycolide nanoparticles induces efficient antitumor and antiviral immune responses. *ACS Nano* 2013, **7**(3)**:** 2042-2055.

187. Tsoras AN, Champion JA. Cross-Linked Peptide Nanoclusters for Delivery of Oncofetal Antigen as a Cancer Vaccine. *Bioconjug Chem* 2018, **29**(3)**:** 776-785.

188. Shirai S, Shibuya M, Kawai A, Tamiya S, Munakata L, Omata D, *et al.* Lipid Nanoparticles Potentiate CpG-Oligodeoxynucleotide-Based Vaccine for Influenza Virus. *Front Immunol* 2019, **10:** 3018.

189. Zhang C, Shi G, Zhang J, Song H, Niu J, Shi S, *et al.* Targeted antigen delivery to dendritic cell via functionalized alginate nanoparticles for cancer immunotherapy. *J Control Release* 2017, **256:** 170-181.

190. Gulla SK, Rao BR, Moku G, Jinka S, Nimmu NV, Khalid S, *et al.* In vivo targeting of DNA vaccines to dendritic cells using functionalized gold nanoparticles. *Biomater Sci* 2019, **7**(3)**:** 773-788.

191. Kim NW, Kim SY, Lee JE, Yin Y, Lee JH, Lim SY, *et al.* Enhanced Cancer Vaccination by In Situ Nanomicelle-Generating Dissolving Microneedles. *ACS Nano* 2018, **12**(10)**:** 9702-9713.

192. Shan W, Zheng H, Fu G, Liu C, Li Z, Ye Y, *et al.* Bioengineered Nanocage from HBc Protein for Combination Cancer Immunotherapy. *Nano Lett* 2019, **19**(3)**:** 1719-1727.

193. Liu S, Jiang Q, Zhao X, Zhao R, Wang Y, Wang Y, *et al.* A DNA nanodevice-based vaccine for cancer immunotherapy. *Nat Mater* 2020, **20:** 421-430.

194. Xu J, Lv J, Zhuang Q, Yang Z, Cao Z, Xu L, *et al.* A general strategy towards personalized nanovaccines based on fluoropolymers for post-surgical cancer immunotherapy. *Nat Nanotechnol* 2020, **15**(12)**:** 1043-1052.

195. Zhu G, Mei L, Vishwasrao HD, Jacobson O, Wang Z, Liu Y, *et al.* Intertwining DNA-RNA nanocapsules loaded with tumor neoantigens as synergistic nanovaccines for cancer immunotherapy. *Nat Commun* 2017, **8**(1)**:** 1482.

196. Zhou L, Hou B, Wang DG, Sun F, Song RD, Shao Q, *et al.* Engineering Polymeric Prodrug Nanoplatform for Vaccination Immunotherapy of Cancer. *Nano Letters* 2020, **20**(6)**:** 4393-4402.

197. Kuai R, Ochyl LJ, Bahjat KS, Schwendeman A, Moon JJ. Designer vaccine nanodiscs for personalized cancer immunotherapy. *Nat Mater* 2017, **16**(4)**:** 489-496.
