# Supplementary material for: STING agonist-boosted mRNA immunization via intelligent design of nanovaccines for enhancing cancer immunotherapy
Source: Natl Sci Rev. 2023 Aug 11;10(10):nwad214. doi: 10.1093/nsr/nwad214 (PMC10484175; doi:10.1093/nsr/nwad214)
Supplement: nwad214_Supplemental_File [file nwad214_supplemental_file.zip › Teaser text.docx]

This paper provides a rational design option to develop mRNA nanovaccine with machine learning technique, leading to effective stimulator of interferon genes (STING) agonist-synergized mRNA immunization.
